# Discovery of 2-Substituted 3-Arylquinoline Derivatives as Potential Anti-Inflammatory Agents Through Inhibition of LPS-Induced Inflammatory Responses in Macrophages

**DOI:** 10.3390/molecules24061162

**Published:** 2019-03-23

**Authors:** Cheng-Yao Yang, Yung-Li Hung, Kai-Wei Tang, Shu-Chi Wang, Chih-Hua Tseng, Cherng-Chyi Tzeng, Po-Len Liu, Chia-Yang Li, Yeh-Long Chen

**Affiliations:** 1 Department of Medicinal and Applied Chemistry, College of Life Science, Kaohsiung Medical University, Kaohsiung City 807, Taiwan; u102850002@kmu.edu.tw (C.-Y.Y.); tzengch@kmu.edu.tw (C.-C.T.); 2 Institute of Health and Sports & Medicine, Juntendo University, 1-1 Hirakagakuendai, Inzai, Chiba 270-1695, Japan; medislove@hotmail.com; 3 School of Pharmacy, College of Pharmacy, Kaohsiung Medical University, Kaohsiung City 807, Taiwan; dadaking1107@gmail.com (K.-W.T.); chihhua@kmu.edu.tw (C.-H.T.); 4 Department of Medical Laboratory Science and Biotechnology, Kaohsiung Medical University, Kaohsiung 807, Taiwan; shuchiwang@kmu.edu.tw; 5 Department of Fragrance & Cosmetic Science, College of Pharmacy, Kaohsiung Medical University, Kaohsiung City 807, Taiwan; 6 Department of Medical Research, Kaohsiung Medical University Hospital, Kaohsiung City 807, Taiwan; 7 Department of Respiratory Therapy, College of Medicine, Kaohsiung Medical University, Kaohsiung 807, Taiwan; kisa@kmu.edu.tw; 8 Graduate Institute of Medicine, College of Medicine, Kaohsiung Medical University, Kaohsiung 807, Taiwan

**Keywords:** anti-inflammatory agents, arylquinoline derivatives, nitric oxide (NO), tumor necrosis factor-á (TNF-á), interleukin-6 (IL-6)

## Abstract

We describe herein the preparation of certain 2-substituted 3-arylquinoline derivatives and the evaluation of their anti-inflammatory effects in LPS-activated murine J774A.1 macrophage cells. Among these newly synthesized 2-substituted 3-arylquinoline derivatives, 2-(4-methoxy- benzoyl)-3-(3,4,5-trimethoxyphenyl)quinoline (**18a**) and 2-(4-fluorobenzoyl)-3-(3,4,5-trimethoxy- phenyl)quinoline (**18b**) are two of the most active compounds which can inhibit the production of NO at non-cytotoxic concentrations. Our results have also indicated that compounds **18a** and **18b** significantly decrease the secretion of pro-inflammatory cytokines (TNF-á and IL-6), inhibit the expression of iNOS, suppress the phosphorylation of MAPKs, and attenuate the activity of NF-êB by LPS-activated macrophages. Through molecular docking analysis, we found that **18b** could fit into the middle of the TNF-á dimer and form hydrophobic interactions with Leu55, Leu57 chain A and B, Tyr59, Val123 chain B and D, Ile 155. These results suggest that both **18a** and **18b** are potential lead compounds in inhibiting LPS-induced inflammatory responses. Further structural optimization to discover novel anti-inflammatory agents is ongoing.

## 1. Introduction

Macrophages are the cornerstone of the innate immune system, which crucially deal with infection of microorganisms and repair of tissue damage by inflammatory responses. It has been considered that macrophages not only contribute to metabolic homeostasis in adipose tissue, liver and pancreas, but are also involved in the development of several diseases such as atherosclerosis, diabetes and cancers [1]. The inflammatory response contributes to antimicrobial defense, tissue repair and metabolism by activated macrophages. The pro-inflammatory mediators and cytokines are secreted when macrophages recognize components of pathogens and debris of dead cells [2]. Lipopolysaccharide (LPS), the Gram-negative bacteria membrane component, is recognized by Toll-like receptor 4 (TLR4) and triggers inflammatory responses via nuclear factor kappa light chain enhancer of activated B cells (NF-êB) and mitogen-activated protein kinases (MAPKs; extracellular signal-regulated kinase (ERK), c-Jun N-terminal kinase (JNK) and p38 mitogen-activated protein kinases (p38 MAPK)) signaling activation, resulting in the expression of inflammatory mediators in mammals such as nitric oxide (NO), tumor necrosis factor-α (TNF-α) and interleukin-6 (IL-6) on activated macrophages [3,4]. Uncontrolled inflammatory responses induced by LPS have been demonstrated to trigger pathogenesis of acute infection diseases such as sepsis and septic shock [5], and also contribute to the development of chronic inflammatory diseases such as type 2 diabetes and cardiovascular diseases [6].

Cyclooxygenase-2 (COX-2) is selectively induced by pro-inflammatory cytokines at the site of inflammation. The pro-inflammatory mediators, NO and cytokines such as TNF-α and IL-6, are generated by activated macrophages, which exert antimicrobial effects and connect to adaptive immunity system [7]. Noteworthy, uncontrolled pro-inflammatory mediator and cytokine production leads to tissue injury and inflammatory diseases [1]. Activated macrophages are major sources of NO production. NO reacts with superoxide O_2_^−^, and converts into cytotoxic molecule peroxynitrite anion (ONOO^−^). The immoderate production of ONOO^−^ causes tissue damage and dysfunction-+ [8]. The pro-inflammatory cytokines, TNF-α and IL-6, are critically contributed to cytokine cascade in sepsis [9] and development of chronic inflammation [10]. Therefore, attenuating the secretion of pro-inflammatory mediators and cytokines is a strategy to prevent inflammatory diseases induced by uncontrolled inflammatory response.

**Figure 1:**
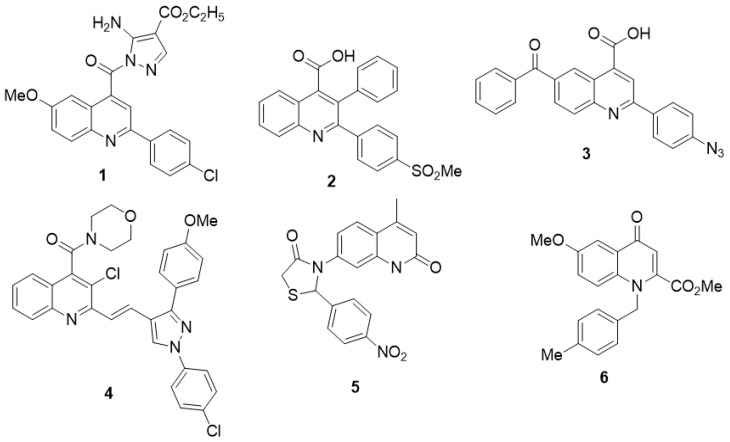
Some anti-inflammatory compounds containing quinoline moieties.

Certain quinoline derivatives (Figure 1) have been reported to possess anti-inflammatory activity. For example, ethyl 5-amino-1-[2-(4-chlorophenyl)-6-methoxyquinoline-4-carbonyl]-1*H*- pyrazole-4-carboxylate (**1**) [11] was found to inhibit COX-2 activity with an IC_50_ value of 0.26 μM. 2-[4-(Methylsulfonyl)phenyl]-3-phenylquinoline-4-carboxylic acid (**2**) [12] and 2-(4-azidophenyl)-6- benzoylquinoline-4-carboxylic acid (**3**) [13] were also proved to be potent COX-2 inhibitors with IC_50_ values of 0.07 and 0.077 μM, respectively. More recently, Chaaban et al. have revealed that the compound **4**, i.e. (*E*)-{3-chloro-2-{2-[1-(4-chlorophenyl)-3-(4-methoxyphenyl)-1*H*-pyrazol-4-yl]vinyl}- quinolin-4-yl} (morpholino)methanone [14] is a selective COX-2 inhibitor with an IC_50_ value of 0.10 μM. 3-(4-Methyl-2-oxo-1,2-dihydroquinolin-7-yl)-2-(4-nitrophenyl)thiazolidin-4-one (**5**) [15] was discovered to show potent anti-inflammatory activity in a carrageenan-induced paw edema model while methyl 6-methoxy-1-(4-methylbenzyl)-4-oxo-1,4-dihydroquinoline-2-carboxylate (**6**) [16] showed potent anti-inflammatory activity with a potency approximately equal to that of indomethacin. Much effort has been devoted to discover new and more effective quinoline-based therapeutics. Over the past few years, We have also synthesized certain polycyclic heterocycles such as furo[3,2:3,4]naphtha[1,2-*d*]imidazole, benzo[*f*]indole-4,9-dione, indeno[1,2-*c*]quinoline and pyrazolo[4,3-*c*]quinoline derivatives for the evaluation of their anti-inflammatory activities [17,18,19,20,21].

Recently, we have identified certain 2-aroyl-3-(4-alkoxyphenyl)quinoline derivatives as inhibitors of dengue virus replication [22]. In continuation of our efforts to explore novel anti-inflammatory agents, we describe herein the preparation of certain 2-substituted 3-arylquinoline derivatives including 2-substituted 3-(3,4,5-trimethoxyphenyl)quinoline derivatives and the evaluation of their anti-inflammatory effects in LPS-activated murine macrophage cell line J774A.1 cells.

We expected these newly synthesized 2-substituted 3-(3,4,5-trimethoxyphenyl)quinoline derivatives to possess potent anti-inflammatory activities on the grounds that 3,4,5-trimethoxyphenyl is a versatile fragment in certain biologically active compounds such as combretastatin A-4, colchicine, etoposide, and trimethoprim.

## 2. Results

### 2.1. Chemistry

#### Preparation of 2-Substituted 3-Arylquinoline Derivatives

**Scheme 1:**
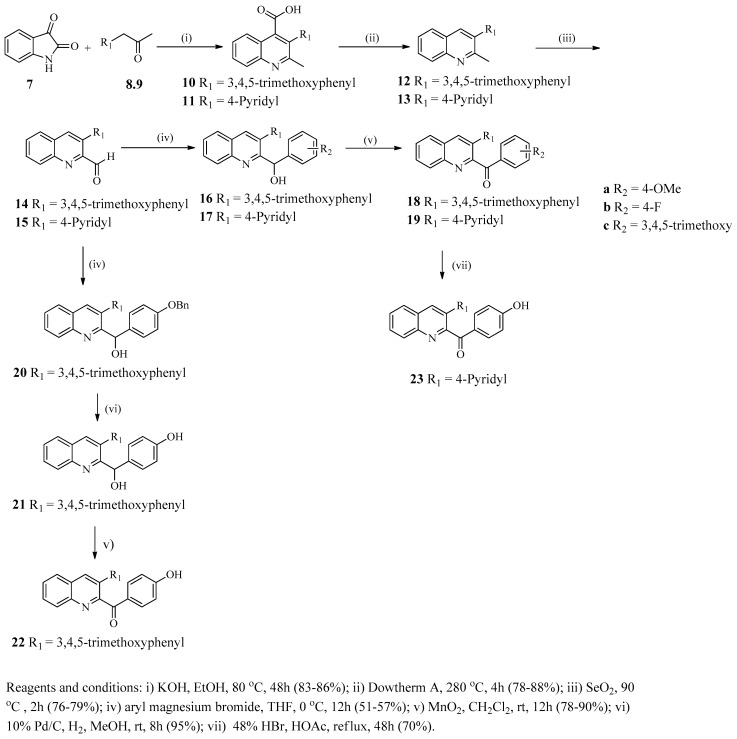
Synthesis of 2-substituted 3-arylquinoline derivatives **16a**–**23**.

The Pfitzinger reaction of indolin-2,3-dione (isatin, **7**) with 3,4,5-trimethoxyphenylacetone (**8**) and (4-pyridyl)acetone (**9**) under basic conditions gave 3-aryl-2-methylquinoline-4-carboxylic acids **10** and **11**, respectively [22,23,24]. Thermal decarboxylation of compounds **10** and **11** gave the 3-aryl-2-methylquinolines **12** and **13** respectively in a yield of 78–88%. Oxidation of compounds **12** and **13** with selenium dioxide afforded 3-arylquinoline-2-carbaldehydes **14** and **15**, respectively, in a yield of 76–79%. Treatment of **14** and **15** with different substituted Grignard reagents afforded 3-aryl-2-(hydroxyphenylmethyl)quinolines **16a**–**c** and **17a**–**c** which was then oxidized with MnO_2_ to give 3-arylquinolines, **18a**–**c** and **19a**–**c**, as described in Scheme 1. Grignard reaction of 3-(3,4,5-trimethoxyphenyl)quinoline-2-carbaldehyde (**14**) afforded compound **20** which was then hydrogenated with Pd/C to give compound **21** in a yield of 95%. Oxidation of compound **21** with MnO_2_ afforded 2-(4-hydroxybenzoyl)-3-(3,4,5-trimethoxyphenyl)quinoline (**22**). Demethylation of 2-(4-methoxybenzoyl)-3-(pyridin-4-yl)quinoline (**19a**) with 48% HBr gave 2-(4-methoxybenzoyl)-3- (pyridin-4-yl)quinoline (**23**). The structure of **10**–**23** was determined by NMR (^1^H and ^13^C) (spectra data can be found in Supplementary Materials) and further confirmed by elemental analysis.

### 2.2. Biological Activities

#### 2.2.1. Effect of 2-substituted 3-arylquinoline Derivatives on NO Production and Cell Survival in Macrophages

**Figure 2:**
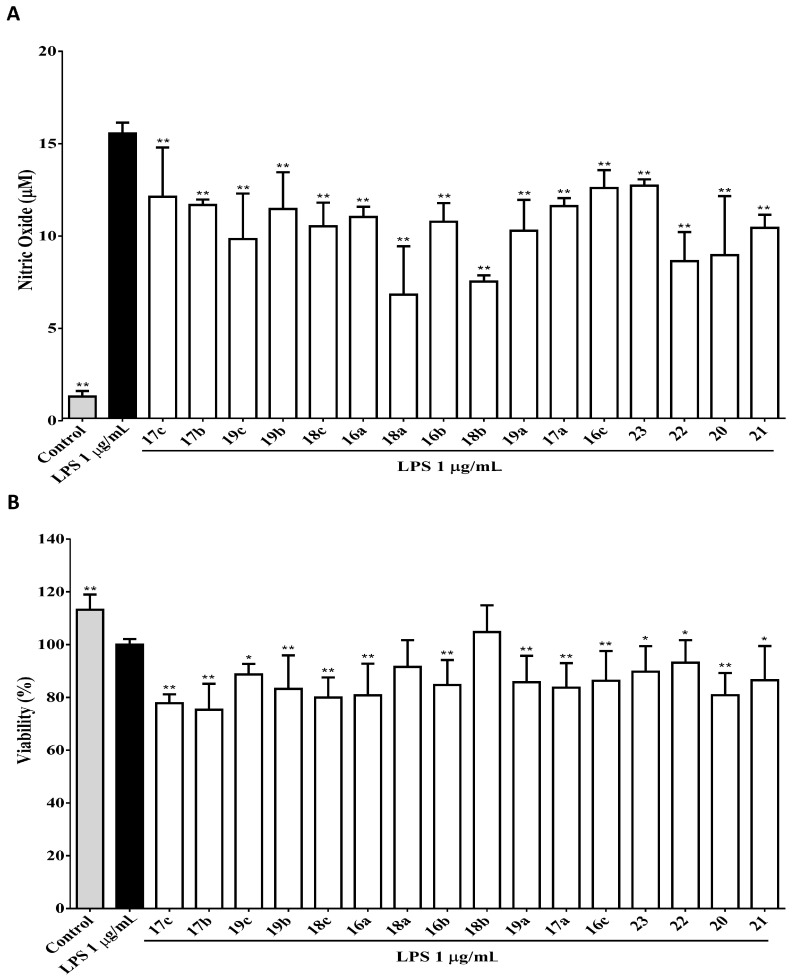
Effects of 2-substituted 3-arylquinoline derivatives on NO production and cell viability by LPS-activated J774A.1 cells. Cells were pre-treated with 10 ìÌ 2-substituted 3-arylquinoline derivatives for 1 h, and then incubated with LPS (1 μg/mL) for 24 h. (**A**) Cell culture supernatant was harvested and quantified the concentration of NO by the Griess reagent. (**B**) Cell viability was analyzed by the MTT assay. Data are presented as means ±SD of three independent experiments. Statistical significance was represented as follows: * *p* < 0.05 and ** *p* < 0.01 vs LPS alone.

NO plays an important role in mediating multiple aspects of inflammatory responses [25]. To examine the effects of 2-substituted 3-arylquinoline derivatives on NO production by LPS-activated macrophages, J774A.1 cells were pre-treated with series of 2-substituted 3-arylquinoline derivatives **16a**–**23** at concentration a 10 μM for 1 h, and were incubated with or without LPS (1 μg/mL) for 24 h. The cell culture supernatants were then harvested and quantified NO concentration using Griess’s method [26], and the cells were used to determine the cell survival by MTT assays. As shown in Table 1 and Figure 2A, LPS stimulation dramatically increased NO production to 15.43 μM compared to the negative control of 1.17 μM. In addition, our results showed that all 2-substituted 3-arylquinoline derivatives significantly reduced LPS-induced NO production by J774A.1 cells (Table 1 and Figure 2A). For the 3-(3,4,5-trimethoxy)phenyl derivatives **18a**–**c** and **22**, the inhibitory activity of NO production decreased in an order of **18a** (R_2_ = 4-OMe, 6.70) > **18b** (R_2_ = 4-F, 7.40) > **22** (R_2_ = 4-OH, 8.52) > **18c** (R_2_ = 3,4,5-tri-OMe, 10.40). These results indicated that the inhibitory activity of monosubstituted 4-OMe, 4-OH, or 4-F compounds are comparable and they are more active than a 3,4,5-trisubstituted group at R_2_ position. A similar trend was observed for compounds **16a**–**c** and **21**, in which the inhibitory activity in the inhibition of NO production of **16a** (R_2_ = 4-OMe, 10.90), **16b** (R_2_ = 4-F, 10.64), and **21** (R_2_ = 4-OH, 10.31) were comparable, while compound **16c** (R_2_ = 3,4,5-tri-OMe, 12.47) was much less active. 

Compound **20** (R_2_ = 4-OBn, 8.83) was especially active suggests that R_2_ is preferably a hydrophobic benzyloxy group. For the 2-substituted derivatives, the benzoyl derivatives, compounds **18a** (6.70), **18b** (7.40), and **18c** (10.4) were more active than their respective hydroxymethylphenyl counterparts, **16a** (10.90), **16b** (10.64), and **16c** (12.47). For the 3-substituted derivatives, the 3,4,5-trimethoxyphenyl derivatives, compounds **16a** (10.90) and **16b** (10.64) were more active than their respective pyridine-4-yl counterparts, **17a** (11.50) and **17b** (11.55). Accordingly, the 3,4,5-trimethoxyphenyl derivatives, compounds **18a** (6.70) and **18b** (7.40), were more active than their respective pyridine-4-yl counterparts, **19a** (10.16) and **19b** (11.34). Among these newly synthesized 2-substituted 3-arylquinoline derivatives, compounds **18a**, **18b**, **20**, and **22** were found to exhibit potent inhibitory activities. However, results of cell viability assay indicated that most of these 2-substituted 3-arylquinoline derivatives exhibited cytotoxicity (cell viability of less than 90%) with the exception of compound **18b** which is non-cytotoxic (Figure 2B). Thus, compounds **18a** (the most active) and **18b** (the least cytotoxic) were selected for further anti-inflammatory evaluations.

#### 2.2.2. Compounds **18a** and **18b** Significantly Suppresses the Production of TNF-α and IL-6 by LPS-Activated Macrophages

LPS stimulates the secretion of proinflammatory cytokines by macrophages such as TNF-á and IL-6 which are critical to the initiation of the inflammatory response; however, hypersecretion of proinflammatory cytokines ultimately result in inflammatory-related diseases or autoimmune diseases [27,28]. The above experiments indicated that compounds **18a** and **18b** were candidate anti-inflammatory compounds among the prepared 2-substituted 3-arylquinoline derivatives.

**Figure 3:**
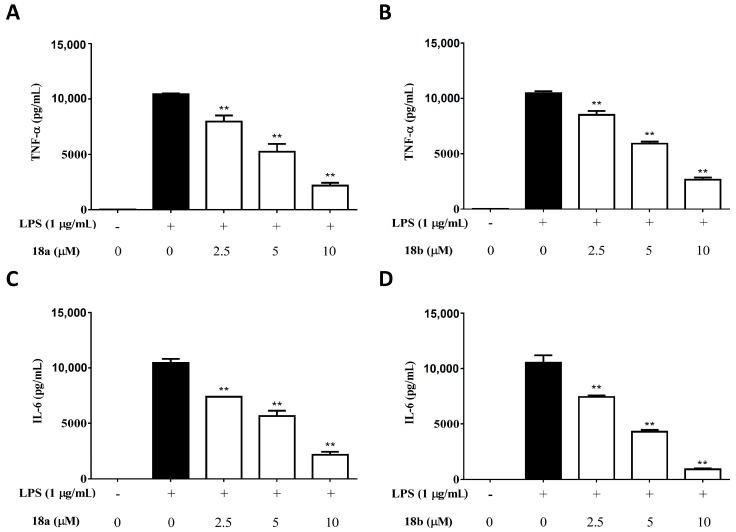
Effects of compouds **18a** and **18b** on the production of pro-inflammatory cytokines by LPS-activated J774A.1 cells. Cells were pre-treated with compounds **18a** and **18b** at different concentrations (0, 2.5, 5 and 10 μM) for 1 h, and then incubated with LPS (1 μg/mL) for 24 h. Cell culture supernatant was harvested and analyzed the concentration of (**A**,**B**) TNF-á and (**C**,**D**) IL-6 by ELISA. Data are presented as means ± SD of three independent experiments. “+”means treated with LPS; “−”means untreated. Statistical significance was represented as follows: * *p* < 0.05 and ** *p* < 0.01 vs LPS alone.

Thus, we investigated the effects of compounds **18a** and **18b** on LPS-induced TNF-α and IL-6 production by macrophages. J774A.1 cells were pre-treated with compounds **18a** or **18b** at different concentrations (0, 2.5, 5 and 10 μM) for 1 h, and were incubated with or without LPS (1 μg/mL) for 24 h. The cell culture supernatants were harvested and quantified TNF-α and IL-6 concentrations by ELISA. Our experimental results indicated that both compounds **18a** and **18b** significantly reduced the production of TNF-α and IL-6 by LPS-activated macrophages (Figure 3).

#### 2.2.3. Compounds 18a and 18b Significantly Attenuate the Activity of NF-êB by LPS-Activated Macrophages

**Figure 4:**
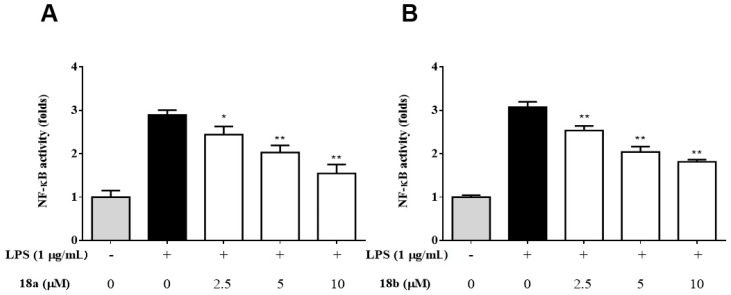
Effects of compounds **18a** (**A**) and **18b** (**B**) on the activity of NF-êB by LPS-activated J-blue cells. Cells were pre-treated with compounds **18a** and **18b** at different concentrations (0, 2.5, 5 and 10 μM) for 1 h, and then incubated with LPS (1 μg/mL) for 24 h. The activity of NF-êB was measured by NF-êB promoter reporter assay. Data are presented as means ± SD of three independent experiments. Statistical significance was represented as follows: * *p* < 0.05 and ** *p* < 0.01 vs LPS alone.

NF-êB is a downstream transcription factor of TLR4, which regulates multiple aspects of innate and adaptive immune functions and serves as a pivotal mediator of inflammatory responses [29]. We further examined whether compounds **18a** and **18b** affected the activity of NF-êB. J-blue cell, a NF-êB reporter J774A.1 macrophage cell line that stably expresses the gene for secreted embryonic alkaline phosphatase (SEAP) inducible by NF-ê B, which was used to detect the activity of NF-êB [30]. J-blue cells were pre-treated with compounds **18a** or **18b** at different concentrations (0, 2.5, 5 and 10 μM) for 1 h, and were incubated with or without LPS (1 μg/mL) for 24 h. The cell culture supernatants were harvested, and the activity of NF-êB was examined by SEAP assay. Our experimental results showed that both compounds **18a** and **18b** significantly attenuated the activity of NF-êB by LPS-activated macrophages (Figure 4).

#### 2.2.4. Compounds **18a** and **18b** Inhibit the Expression of iNOS and Suppress the Phosphorylation of MAPKs

**Figure 5:**
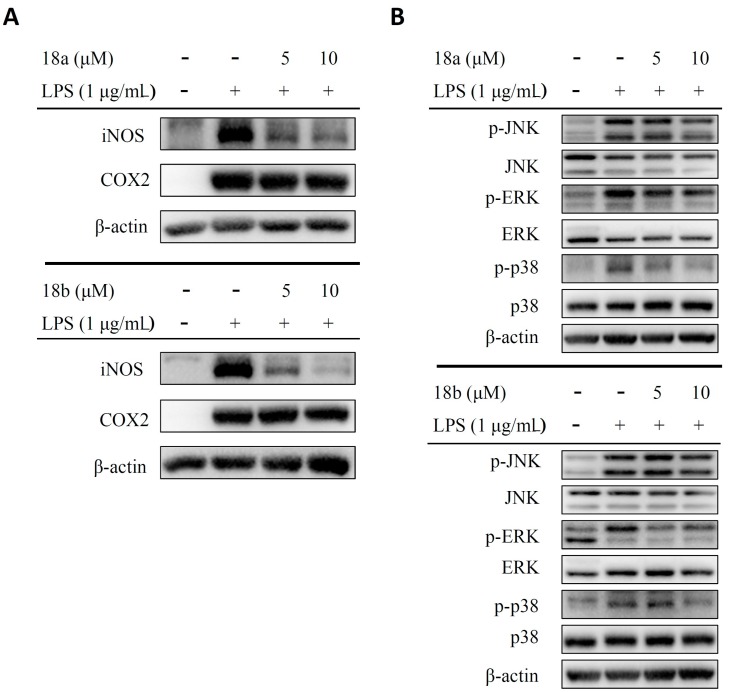
Effects of compounds **18a** and **18b** on the inducible nitric oxide synthase (iNOS), cyclooxygenase 2 (COX-2) and mitogen-activated protein kinases (MAPKs) expression by lipopolysaccharide (LPS)-activated J-blue cells. Cells were pre-treated with compounds **18a** and **18b** at different concentrations (0, 5 and 10 μM) for 1 h, and then incubated with LPS (1 μg/mL) for 2h (for MAPKs expression detection) or 24 h (for iNOS and COX-2 expression detection). (**A**) The expression of iNOS, and COX-2 was examined by Western blot. (**B**) The expression of phosphor- extracellular signal-regulated kinase (ERK), ERK, phosphor-N-terminal kinase (JNK), JNK, phosphor-p38 and p38 was measured by Western blot. Blots are representative of results obtained in three separate experiments. The expression of β-actin was used as an internal control.

To further examine whether compounds **18a** and **18b** affect iNOS, COX2 and MAPKs expression, J774A.1 cells were pre-treated with compound **18a** or **18b** at different concentrations (0, 5 and 10 μM) for 1 h, and then incubated with or without LPS (1 μg/mL) for 2h (for MAPKs expression detection) or 24 h (for iNOS and COX2 expression detection). The protein level of iNOS, COX2, phosphor-ERK, ERK, phosphor-JNK, JNK, phosphor-p38 and p38 was measured by western blot. As shown in Figure 5A, compounds **18a** and **18b** inhibit LPS-induced iNOS expression in J774A.1 cells, but not COX2. In addition, we have also found that compounds **18a** and **18b** suppress the phosphorylation of ERK, JNK and p38 by LPS-activated J774A.1 cells (Figure 5B).

#### 2.2.5. Molecular Docking Results of **18a**, **18b** and TNF-á

**Figure 6:**
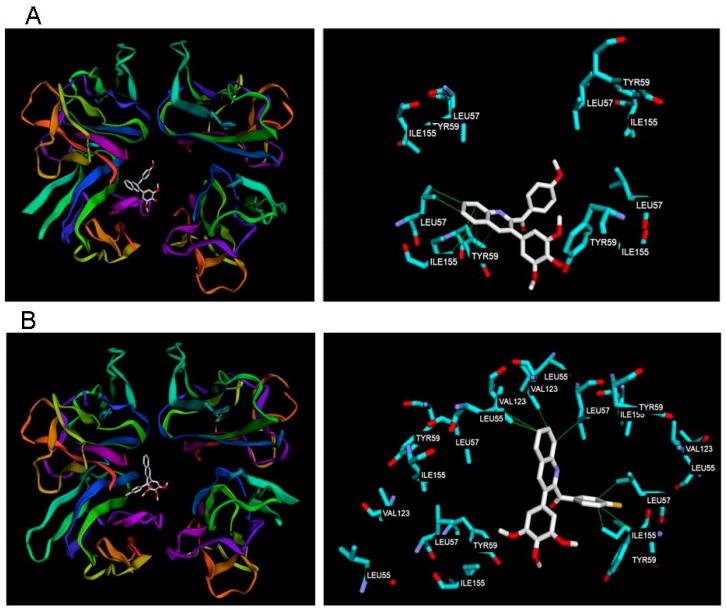
Docking poses of compound **18a** (**A**) and **18b** (**B**) at the TNF-á (PDB code 2AZ5). The interaction illustrated by green lines represents hydrophobic interaction.

To understand the interaction between our hit compounds **18a**–**b** and TNF-á (PDB code 2AZ5) [31,32], we performed a molecular docking study using the Achilles Blind Docking Server. The docking pose with the lowest binding energy of compounds **18a**–**b** was shown in Figure 6. The results showed that both compounds **18a** and **18b** could fit into the middle of TNF-á dimer. According to the docking results, compound **18a** formed hydrophobic interactions with Leu55, Tyr59, Ile 155 and pi-stacking interaction with Tyr59, while compound **18b** also formed hydrophobic interaction with Leu55, Leu57 chain A and B, Tyr59, Val123 chain B and D, Ile 155. The lowest binding energy score between compound **18a**, **18b** and TNF-á was-7.00 and −8.30 kcal/mol individually.

## 3. Discussion

The innate immune response constitutes the first line of defense against invading pathogens and plays an important role in inflammatory diseases [33]. Sepsis is a severe illness in which the bloodstream is overwhelmed with bacteria, resulting in inflammation throughout the body and causing more than 215,000 deaths annually just in the United States [34]. LPS is well known to trigger an inflammatory response leading to release of inflammatory mediators and occasionally sepsis, or even septic shock [4]. In the present study, we synthesized a total of sixteen 2-substituted 3-arylquinoline derivatives and evaluated the anti-inflammatory effect in LPS-activated murine macrophage cell line J774A.1 cells. We firstly examined the effect of 2-substituted 3-arylquinoline derivatives on the production of NO by LPS-activated macrophages. We found that compounds **18a** and **18b** significantly reduced the production of NO by LPS-activated macrophages at 10 ìM with no toxicity. In addition, compounds **18a** and **18b** also repressed the expression of iNOS by LPS-activated macrophages. NO has been regarded that is crucial in pathogenesis of autoimmune diseases such as rheumatoid arthritis and chronic inflammatory diseases such as atherosclerosis [35]. NO converts to ONOO^−^ by O_2_^−^, and ONOO^−^ exerts cytotoxicity effects. The overproduction of NO strongly contributes to tissue damage and augmentation of inflammation. Our results suggest that compounds **18a** and **18b** have potential in inhibiting the production of NO in LPS-induced sepsis.

Abnormal macrophage activity causes overproduction of pro-inflammatory mediators and cytokines, and triggers several inflammatory diseases [1]. To reduce production of pro- inflammatory mediators and cytokines by activated macrophages is a critical role in prevention of immoderate inflammation. TNF-α and IL-6 are major pro-inflammatory cytokines that are produced by LPS-activated macrophages [3], and critically augment inflammatory cascades [36]. The uncontrolled TNF-α and IL-6 production lead to metabolic disorders such as type 2 diabetes and atherosclerosis [10,37]. Thus, to inhibit the overproduction of pro-inflammatory mediators and cytokines is a key point for prevention of inflammatory diseases. We further investigated the effects of compounds **18a** and **18b** on LPS-induced TNF-α and IL-6 productions. Our experimental results indicated that compounds **18a** and **18b** exert inhibitory effects on LPS-induced TNF-α and IL-6 productions. These results suggest that compounds **18a** and **18b** might have benefit on the prevention of overactive inflammatory responses induced by microbe’s infection.

Activation of the transcription factor NF-êB and MAPKs is commonly thought to be critical to LPS-stimulated macrophage inflammatory mediator production including NO, TNF-α and IL-6 [4,5,6]. Therefore, we investigated whether compounds **18a** and **18b** decrease the production of NO, TNF-α and IL-6 through regulating NF-êB and MAPKs signaling pathways. Our experimental results demonstrated that compounds **18a** and **18b** attenuate the activity of NF-êB and suppress the phosphorylation of MAPKs in LPS-activated macrophages. These results suggest that compounds **18a** and **18b** inhibit the production of NO, TNF-α and IL-6 by LPS-activated macrophages through NF-êB and MAPKs signaling pathways.

In the molecular docking study, the scores of compound **18b** are better than those of compound **18a**. Moreover, compound **18b** showed an excellent lowest binding energy with no hydrogen bond interaction. It could be explained that our hit compounds **18a** and **18b**, were able to form a stable complex with TNF-α by docking into the middle pocket of dimer. The previous study showed that the complex retained the same basic structural subunit fold compared to native dimer, but the introduction of small molecular may cause the angle between the subunits slightly widened [38]. In our study, we suggested that both compound **18a** and **18b** may change the angle of TNF-α dimer between the subunits and lead to inactivation to affect the downstream inflammatory reaction.

## 4. Materials and Methods

### 4.1. General Information

Melting points were determined on an IA9100 melting point apparatus (Dubuque, IA, USA) and are uncorrected. Nuclear magnetic resonance (^1^H and ^13^C) spectra were recorded on a Gemini 200 or Unity-400 spectrometer (Varian, Palo Alto, CA, USA). Chemical shifts were expressed in parts per million (δ) with tetramethylsilane (TMS) as an internal standard. Analytical TLC was performed on Art. 5554 Kieselgel 60 GF254 produced by Merck (Darmstadt, Germany) and the spots of compounds were detected with UV light indicator irradiated at 254 and 366 nm. Art. 7734 Kieselgel 60 GF254 (70–400 mesh, Merck) was used for column chromatography. The elemental analyses were performed in the Instrument Center of National Science Council at National Cheng-Kung University and National Taiwan University using Heraeus CHN-O Rapid EA, and all values are within ± 0.4% of the theoretical compositions.

### 4.2. Chemistry

#### 4.2.1. General Procedure for the Preparation of 3-aryl-2-methylquinoline-4-carboxylic Acids **10** and **11**

A mixture of isatin **7** (40 mmol), phenylacetone **8** or **9** (48 mmol) and KOH (6.74 g, 120 mmol) in EtOH was heated at 80 °C for 48 h (TLC monitoring). After cooling, the solvent was removed in vacuo and the residue dissolved in H_2_O (50 mL), and the aqueous solution was washed twice with Et_2_O (30 mL). The ice-cold aqueous phase was acidified to pH 1 with 37% HCl, and the precipitate was collected by suction filtration, washed with H_2_O and recrystallized with EtOH to give 3-aryl-2-methylquinoline-4-carboxylic acids **10** and **11**.

*2-Methyl-3-(3,4,5-trimethoxyphenyl)quinoline-4-carboxylic acid* (**10**)

Yield 83% as a white solid. Mp 305–306 °C. ^1^H-NMR (400 MHz, DMSO-*d*_6_) ä 2.54 (s, 3H, 2-Me), 3.74 (s, 3H, OMe), 3.78 (s, 6H, OMe), 6.71 (s, 2H, Ar-H), 7.63–7.67 (m, 1H, 7-H), 7.78–7.82 (m, 2H, 5-H and 6-H), 8.04 (d, 1H, *J* = 8.4 Hz, 8-H), 13.62 (br s, 1H, -COOH). ^13^C-NMR (100 MHz, DMSO-*d*_6_) ä 24.6, 56.0 (2C), 60.1, 107.0 (2C), 121.7, 124.8, 127.1, 128.5, 129.9, 130.4, 132.1, 137.0, 140.0, 146.2, 152.6 (2C), 157.7, 168.3. Anal. calcd for C_20_H_19_NO_5_ 0.7 HCl: C 63.39, H 5.24, N 3.70; found: C 63.28, H 5.64, N 3.65.

*2-Methyl-3-(pyridin-4-yl)quinoline-4-carboxylic acid* (**11**)

Yield 86% as a white solid. Mp 336–337 °C. ^1^H-NMR (400 MHz, DMSO) ä 2.58 (s, 3H, 2-Me), 7.81–7.85 (m, 1H, 7-H), 7.99–8.03 (m, 1H, 5-H and 6-H), 8.09–8.11 (m, 2H, pyridinyl-H), 8.31 (d, 1H, *J* = 9.2 Hz, 8-H), 9.06-9.08 (m, 2H, pyridinyl-H). ^13^C-NMR (100 MHz, DMSO) ä 23.1, 121.6, 125.7, 126.0, 127.5 (2C), 128.7, 132.4, 142.0, 143.5 (2C), 143.9, 152.1, 156.1, 166.6. Anal. calcd for C_16_H_12_N_2_O_2_ 0.5 H_2_O 0.3 HCl: C 67.60, H 4.73, N 9.86; found: C 67.49, H 4.85, N 9.93.

#### 4.2.2. General Procedure for the Decarboxylation of Acids **12** and **13**

The suspension of quinoline-4-carboxylic acid **10** or **11** (5.0 mmol) in 10 mL Dowtherm was heated to 280 °C for 4 h (TLC monitoring). After cooling, the reaction mixture was added *n*-hexane (50 mL) and the precipitate was collected by suction filtration and washed with *n*-hexane. The crude product was purified by flash chromatography on silica gel (hexane/CH_2_Cl_2_ = 1/1) and recrystallized from EtOH to give compounds **12** and **13**.

*2-Methyl-3-(3,4,5-trimethoxyphenyl)quinoline* (**12**)

Yield 88% as a yellow liquid. ^1^H-NMR (400 MHz, CDCl_3_) ä 2.71 (s, 3H, 2-Me), 3.90 (s, 6H, OMe), 3.94 (s, 3H, OMe), 6.60 (s, 2H, Ar-H), 7.48–7.52 (m, 1H, 7-H), 7.67–7.72 (m, 1H, 6-H), 7.78–7.80 (m, 1H, 5-H), 7.97 (s, 1H, 4-H), 8.07 (d, 1H, *J* = 8.4 Hz, 8-H). ^13^C-NMR (100 MHz, CDCl_3_) ä 24.5, 56.1 (2C), 60.8, 106.3 (2C), 126.0, 126.5, 127.3, 128.2, 129.3, 135.4, 135.6, 135.7, 137.3, 146.9, 153.0 (2C), 157.2. Anal. calcd for C_19_H_19_NO_3_ 0.3H_2_O: C 72.50, H 6.28, N 4.45; found: C 72.24, H 6.25, N 4.37.

*2-Methyl-3-(pyridin-4-yl)quinoline* (**13**)

Yield 78% as a brown solid. Mp 59–60 °C. ^1^H-NMR (400 MHz, CDCl_3_) ä 2.68 (s, 3H, 2-Me), 7.37–7.38 (m, 2H, pyridinyl-H), 7.52–7.56 (m, 1H, 7-H), 7.72–7.76 (m, 1H, 6-H), 7.81–7.83 (m, 1H, 5-H), 7.98 (s, 1H, 4-H), 8.08 (d, 1H, *J* = 8.4 Hz, 8-H), 8.75 (br s, 2H, pyridinyl-H). ^13^C-NMR (100 MHz, CDCl_3_) ä 24.4, 124.2, 126.5, 126.5, 127.5 (2C), 128.5, 130.1, 132.9, 136.2, 147.4, 147.8, 149.9 (2C), 156.1. Anal. calcd for C_15_H_12_N_2_ 1.2 H_2_O: C 74.48, H 6.00, N 11.58; found: C 74.66, H 6.06, N 11.92.

#### 4.2.3. General Procedure for the Preparation of quinoline-2-carbaldehydes **14** and **15**

A mixture **12** or **13** (3.0 mmol) and selenium dioxide (0.66 g, 6.0 mmol) in 1,4-dioxane (50 mL) was heated to 100 °C for 2 h (TLC monitoring). The mixture was diluted with saturated aqueous NaHCO_3_ (50 mL) and extracted with CH_2_Cl_2_ (100 mL × 3). The combined organic layers were washed with H_2_O followed by brine, dried with MgSO_4_ and the solvent was removed in vacuo. The residue was recrystallized with EtOH to give quinoline-2-carbaldehydes **14** and **15**.

*3-(3,4,5-Trimethoxyphenyl)quinoline-2-carbaldehyde* (**14**)

Yield 79% as a yellow solid. Mp 140–141 °C. ^1^H NMR (400 MHz, CDCl_3_) ä 3.90 (s, 6H, OMe), 3.95 (s, 3H, OMe), 6.64 (s, 2H, Ar-H), 7.70–7.74 (m, 1H, 7-H), 7.82–7.86 (m, 1H, 6-H), 7.93 (d, 1H, *J* = 8.4 Hz, 5-H), 8.25 (s, 1H, 4-H), 8.34 (d, 1H, *J* = 8.4 Hz, 8-H), 10.28 (s, 1H, CHO). ^13^C-NMR (100 MHz, CDCl_3_) ä 56.2 (2C), 61.0, 106.9 (2C), 127.5, 128.8, 129.5, 130.6 (2C), 132.4, 135.9, 138.1 (2C), 138.3, 147.1, 149.8, 153.2, 192.3 (CHO). Anal. calcd for C_19_H_17_NO_4_: C 70.58, H 5.30, N 4.33; found: C 70.24, H 5.20, N 4.24.

*3-(Pyridin-4-yl)quinoline-2-carbaldehyde* (**15**)

Yield 76% a yellow solid. Mp 185–186 °C. ^1^H-NMR (400 MHz, CDCl_3_) ä 7.34–7.35 (m, 2H, pyridinyl-H), 7.74–7.78 (m, 1H, 7-H), 7.87–7.95 (m, 1H, 6-H), 7.94 (d, 1H, *J* = 8.0 Hz, 5-H), 8.16 (s, 1H, 4-H), 8.33 (d, 1H, *J* = 8.4 Hz, 8-H), 8.71–8.73 (m, 2H, pyridinyl-H), 10.26 (s, 1H, CHO). ^13^C-NMR (100 MHz, CDCl_3_) ä 124.1 (2C), 124.4, 127.8, 130.0, 130.3, 131.1, 132.2, 138.7, 145.8, 147.4, 149.3, 149.6 (2C), 192.6 (CHO). Anal. calcd for C_15_H_10_N_2_O 0.7 H_2_O: C 72.98, H 4.65, N 11.35; found: C 72.88, H 4.33, N 11.08.

#### 4.2.4. General Procedure for the Preparation of 2-(hydroxyphenylmethyl)-3-phenylquinolines **16a**–**c** and **17a**–**c**

A mixture **14** or **15** (1.0 mmol), aryl magnesium bromide (3 mmol, 3 mL of a 1 M solution in THF), and THF (30 mL) was stirred at 0 °C for 12 h (TLC monitoring). The reaction was quenched by addition of water (3 mL) and partitioned between H_2_O (50 mL) and CH_2_Cl_2_ (50 mL). The organic layer was washed with brine, dried over MgSO_4_ and the solvent was removed in vacuo. The residue was purified by flash chromatography on silica gel (*n*-hexane/CH_2_Cl_2_ = 3/2) and recrystallized from EtOH to give compounds **16a**–**c** and **17a**–**c**.

*2-[1-(4-Methoxyphenyl)-1-hydroxylmethyl]-3-(3,4,5trimethoxyphenyl)quinoline* (**16a**)

Yield 57% as a white solid. Mp 115–116 °C. ^1^H-NMR (400 MHz, CDCl_3_) ä 3.68 (s, 6H, OMe), 3.70 (s, 3H, OMe), 3.91 (s, 3H, OMe), 5.87 (s, 1H, 2-CH), 6.14 (s, 2H, Ar-H), 6.26 (br s, 1H, OH), 6.63 (d, 2H, *J* = 8.8 Hz, Ar-H), 6.74 (d, 2H, *J* = 8.8 Hz, Ar-H), 7.58–7.62 (m, 1H, 7-H), 7.77–7.86 (m, 2H, 5-H and 6-H), 7.95 (s, 1H, 4-H), 8.21 (d, 1H, *J* = 8.4 Hz, 8-H). ^13^C-NMR (100 MHz, CDCl_3_) ä 55.2, 55.9, 56.0, 61.0, 72.5, 106.5 (2C), 113.5 (2C), 127.0, 127.4, 127.5, 128.7, 129.1 (2C), 129.9, 133.3, 134.2, 134.9, 137.1, 137.6, 145.2, 152.9 (2C), 158.9, 158.9. Anal. calcd for C_26_H_25_NO_5_: C 72.37, H 5.84, N 3.25; found: C 72.10, H 5.87, N 3.19.

*2-[1-(4-Fluorophenyl)-1-hydroxylmethyl]-3-(3,4,5-trimethoxyphenyl)quinoline* (**16b**)

Yield 52% as a white solid. Mp 118–119 °C. ^1^H-NMR (400 MHz, CDCl_3_) ä 3.69 (s, 6H, OMe), 3.91 (s, 3H, OMe), 5.95 (s, 1H, 2-CH), 6.16 (s, 2H, Ar-H), 6.76–6.78 (m, 4H, Ar-H), 7.58–7.62 (m, 1H, 7-H), 7.77–7.86 (m, 2H, 5-H and 6-H), 7.97 (s, 1H, 4-H), 8.22 (d, 1H, *J* = 8.4 Hz, 8-H). ^13^C-NMR (100 MHz, CDCl_3_) ä 55.9 (2C), 60.9, 72.2, 106.4 (2C), 114.7 (2C, *J* = 21.2 Hz), 127.1, 127.3, 127.4, 128.5, 129.4 (2C, *J* = 8.3 Hz), 130.0, 133.0, 134.0, 137.2, 137.7, 138.4 (*J* = 3.0 Hz), 145.0, 153.0 (2C), 158.3, 161.9 (*J* = 244.0 Hz). Anal. calcd for C_25_H_22_FNO_4_: C 71.59, H 5.29, N 3.34; found: C 71.43, H 5.21, N 3.34.

*2-[1-(3,4,5-Trimethoxyphenyl)-1-hydroxylmethyl]-3-(3,4,5-trimethoxyphenyl)quinoline* (**16c**)

Yield 54% as a white solid. Mp 126–127 °C. ^1^H-NMR (400 MHz, CDCl_3_) ä 3.64 (s, 6H, OMe), 3.72 (s, 6H, OMe),3.74 (s, 3H, OMe), 3.90 (s, 3H, OMe), 5.89 (s, 1H, 2-CH), 6.09 (s, 2H, Ar-H), 6.25 (s, 2H, Ar-H), 6.31 (br s, 1H, OH), 7.60–7.64 (m, 1H, 7-H), 7.79–7.87 (m, 2H, 5-H and 6-H), 7.98 (s, 1H, 4-H), 8.23 (d, 1H, *J* = 8.4 Hz, 8-H). ^13^C-NMR (100 MHz, CDCl_3_) ä 55.9 (2C), 56.1 (2C), 60.6, 60.9, 73.1, 104.8 (2C), 106.7 (2C), 127.1, 127.4, 127.5, 128.7, 130.0 (2C), 133.2, 134.1, 137.4 (2C), 137.6, 138.1, 145.2, 152.9 (2C), 153.0, 158.4. Anal. calcd for C_28_H_29_NO_7_: C 68.42, H 5.95, N 2.85; found: C 68.76, H 5.91, N 2.81.

*2-[1-(4-Methoxyphenyl)-1-hydroxylmethyl]-3-(pyridin-4-yl)quinoline* (**17a**)

Yield 53% as a yellow solid. Mp 114–115 °C. ^1^H-NMR (400 MHz, CDCl_3_) ä 3.71 (s, 3H, OMe), 5.86 (s, 1H, 2-CH), 6.21 (br s, 1H, OH), 6.58–6.62 (m, 2H, Ar-H), 6.66–6.70 (m, 2H, Ar-H), 6.97–6.98 (m, 2H, pyridinyl-H), 7.62–7.66 (m, 1H, 7-H), 7.82–7.87 (m, 2H, 5-H and 6-H), 7.93 (s, 1H, 4-H), 8.24 (d, 1H, *J* = 8.4 Hz, 8-H), 8.57 (br s, 2H, pyridinyl-H). ^13^C-NMR (100 MHz, CDCl_3_) ä 55.2, 72.5, 113.7 (2C), 124.3, 127.1, 127.4, 127.6, 128.8, 129.1 (2C), 130.5, 131.5, 134.0, 137.3 (2C), 145.4, 146.3, 149.5 (2C), 157.9, 159.0. Anal. calcd for C_22_H_18_N_2_O_2_ 0.1 H_2_O: C 76.77, H 5.33, N 8.14; found: C 76.60, H 5.62, N 7.72.

*2-[1-(4-Fluorophenyl)-1-hydroxylmethyl]-3-(pyridin-4-yl)quinoline* (**17b**)

Yield 51% as a white solid. Mp 162–163 °C. ^1^H-NMR (400 MHz, CDCl_3_) ä 5.91 (d, 1H, *J* = 5.6 Hz, 2-CH), 6.31 (d, 1H, *J* = 5.6 Hz, OH), 6.72–6.75 (m, 4H, Ar-H), 6.96–6.98 (m, 2H, pyridinyl-H), 7.63–7.67 (m, 1H, 7-H), 7.83–7.88 (m, 2H, 5-H and 6-H), 7.95 (s, 1H, 4-H), 8.24 (d, 1H, *J* = 8.4 Hz, 8-H), 8.57–8.58 (m, 2H, pyridinyl-H). ^13^C-NMR (100 MHz, CDCl_3_) ä 72.2, 115.3 (2C, *J* = 21.2 Hz), 124.1 (2C), 127.2, 127.5, 127.6, 128.7, 129.5 (2C, *J* = 8.4 Hz), 130.7, 131.4, 137.5, 137.6 (*J* = 3.0 Hz), 145.4, 146.0, 149.8 (2C), 157.3, 162.2 (*J* = 244.8 Hz). Anal. calcd for C_21_H_15_FN_2_O 0.1 H_2_O: C 75.94, H 4.61, N 8.43; found: C 75.91, H 4.48, N 8.42.

*2-[1-(3,4,5-Trimethoxyphenyl)-1-hydroxylmethyl]-3-(pyridin-4-yl)quinoline* (**17c**)

Yield 55% as a yellow liquid. ^1^H-NMR (400 MHz, CDCl_3_) ä 3.57 (s, 6H, OMe), 3.74 (s, 3H, OMe), 5.86 (s, 1H, 2-CH), 5.98 (s, 2H, Ar-H), 6.38 (br s, 1H, OH), 7.03–7.05 (m, 2H, pyridinyl-H), 7.62–7.66 (m, 1H, 7-H), 7.82–7.88 (m, 2H, 5-H and 6-H), 7.95 (s, 1H, 4-H), 8.23 (d, 1H, *J* = 8.4 Hz, 8-H), 8.59–8.60 (m, 2H, pyridinyl-H). ^13^C-NMR (100 MHz, CDCl_3_) ä 55.7 (2C), 60.6, 73.0, 104.6 (2C), 124.3 (2C), 127.0, 127.35, 127.6, 128.6, 130.5, 131.3, 137.1, 137.3, 137.4, 145.3, 146.1, 149.5 (2C), 152.8 (2C), 157.26.

#### 4.2.5. General Procedure for Preparation of 2-benzoyl-3-phenylquinolines **18a**–**c** and **19a**–**c**

A mixture of **16a**–**c** or **17a**–**c** (1.0 mmol) and MnO_2_ (10 mmol) in CH_2_Cl_2_ (20 mL) was stirred at room temperature for 12 h (TLC monitoring). The reaction mixture was partitioned between H_2_O (50 mL) and CH_2_Cl_2_ (50 mL). The organic layer was washed with brine, dried over MgSO_4_ and the solvent was removed in vacuo. The residue was recrystallized from MeOH to give compounds **18a**–**c** and **19a**–**c**.

*2-(4-Methoxybenzoyl)-3-(3,4,5-trimethoxyphenyl)quinoline* (**18a**)

Yield 85% as a white solid. Mp 150–151 °C. ^1^H-NMR (400 MHz, CDCl_3_) ä 3.69 (s, 6H, OMe), 3.82 (s, 3H, OMe), 3.85 (s, 3H, OMe), 6.62 (s, 2H, Ar-H), 6.87-6.91 (m, 2H, Ar-H), 7.63–7.67 (m, 1H, 7-H), 7.75–7.79 (m, 1H, 6-H) 7.85–7.90 (m, 2H, Ar-H), 7.92–7.95 (m, 1H, 5-H), 8.17 (d, 1H, *J* = 8.4 Hz, 8-H), 8.28 (s, 1H, 4-H). ^13^C-NMR (100 MHz, CDCl_3_) ä 55.5, 55.9 (2C), 60.8, 106.3 (2C), 113.8 (2C), 127.7, 127.8, 127.9, 129.4, 129.5, 130.1, 132.7 (2C), 133.1, 133.6, 136.5, 137.8, 146.0, 153.2 (2C), 156.9, 164.0, 194.0 (C=O). Anal. calcd for C_26_H_23_NO_5_ 0.1 H_2_O: C 72.41, H 5.42, N 3.25; found: C 72.21, H 5.43, N 3.16.

*2-(4-Fluorobenzoyl)-3-(3,4,5-trimethoxyphenyl)quinoline* (**18b**)

Yield 81% a white solid. Mp 147–148 °C. ^1^H-NMR (400 MHz, CDCl_3_) ä 3.70 (s, 6H, OMe), 3.83 (s, 3H, OMe), 6.57 (s, 2H, Ar-H), 7.07–7.11 (m, 2H, Ar-H), 7.65–7.69 (m, 1H, 7-H), 7.77–7.82 (m, 1H, 6-H), 7.91–7.95 (m, 3H, Ar-H and 5-H), 8.18 (d, 1H, *J* = 8.4 Hz, 8-H), 8.30 (s, 1H, 4-H). ^13^C-NMR (100 MHz, CDCl_3_) ä 56.0 (2C), 60.9, 106.4 (2C), 115.8 (2C, *J* = 21.2 Hz), 127.7, 128.0, 128.1, 129.5, 130.3, 132.7 (*J* = 3.0 Hz), 133.0 (2C, *J* = 9.9 Hz), 133.7, 136.8, 137.9, 146.0, 153.3 (2C), 156.1, 166.0 (*J* = 254.7 Hz), 193.7 (C=O). Anal. calcd for C_25_H_20_FNO_4_: C 71.93, H 4.83, N 3.36; found: C 71.57, H 4.93, N 3.28.

*2-(3,4,5-Trimethoxybenzoyl)-3-(3,4,5-trimethoxyphenyl)quinoline* (**18c**)

Yield 83% as a white solid. Mp 139–140 °C. ^1^H-NMR (400 MHz, CDCl_3_) ä 3.71 (s, 6H, OMe), 3.80 (s, 6H, OMe), 3.84 (s, 3H, OMe), 3.91 (s, 3H, OMe), 6.62 (s, 2H, Ar-H), 7.18 (s, 2H, Ar-H), 7.65–7.69 (m, 1H, 7-H), 7.77–7.81 (m, 1H, 6-H), 7.95 (d, 1H, *J* = 8.4 Hz, 5-H), 8.19 (d, 1H, *J* = 8.4 Hz, 8-H), 8.30 (s, 1H, 4-H). ^13^C-NMR (100 MHz, CDCl_3_) ä 55.9 (2C), 56.3 (2C), 60.9, 61.0, 106.3 (2C), 107.9 (2C), 127.7, 128.0, 128.0, 129.6, 130.2, 131.3, 133.1, 133.7, 136.6, 137.8, 143.2, 146.0, 153.0 (2C), 153.3 (2C), 156.4, 194.1 (C=O). Anal. calcd for C_28_H_27_NO_7_: C 68.70, H 5.56, N 2.86; found: C 68.44, H 5.59, N 2.81.

*2-(4-Methoxybenzoyl)-3-(pyridin-4-yl)quinoline* (**19a**)

Yield 78% a white solid. Mp 152–153 °C. ^1^H-NMR (400 MHz, CDCl_3_) ä 3.88 (s, 3H, OMe), 6.93 (d, 2H, *J* = 8.8 Hz, Ar-H), 7.35–7.36 (m, 2H, pyridinyl-H), 7.68–7.72 (m, 1H, 7-H), 7.82–7.86 (m, 1H, 6-H), 7.91 (d, 2H, *J* = 8.8 Hz, Ar-H), 7.96 (d, 1H, *J* = 8.4 Hz, 5-H), 8.21 (d, 1H, *J* = 8.4 Hz, 8-H), 8.29 (s, 1H, 4-H), 8.58–8.59 (m, 2H, pyridinyl-H). ^13^C-NMR (100 MHz, CDCl_3_) ä 55.6, 114.0 (2C), 123.8, 127.7, 127.9, 128.4, 129.0, 129.8, 130.9, 131.4, 133.0 (2C), 137.5 (2C), 146.3, 146.4, 149.6 (2C), 155.8, 164.3, 192.9 (C=O). Anal. calcd for C_22_H_16_N_2_O_2_: C 77.63, H 4.74, N 8.23; found: C 77.23, H 4.90, N 8.19.

*2-(4-Fluorobenzoyl)-3-(pyridin-4-yl)quinoline* (**19b**)

Yield 83% as a white solid. Mp 139–140 °C. ^1^H-NMR (400 MHz, CDCl_3_) ä 7.10–7.16 (m, 2H, Ar-H), 7.30–7.32 (m, 2H, pyridinyl-H), 7.58–7.73 (m, 1H, 7-H), 7.81–7.85 (m, 1H, 6-H), 7.95–8.01 (m, 3H, Ar-H and 5-H), 8.19 (d, 1H, *J* = 8.4 Hz, 8-H), 8.30 (s, 1H, 4-H), 8.58–8.60 (m, 2H, pyridinyl-H). ^13^C-NMR (100 MHz, CDCl_3_) ä 115.8 (2C, *J* = 22.0 Hz), 123.6, 127.8, 127.9, 128.6, 129.7, 131.0, 131.6, 132.3 (*J* = 3.0 Hz), 133.3 (2C, *J* = 9.8 Hz), 137.6 (2C), 145.7, 146.3, 149.9 (2C), 154.9, 166.2 (*J* = 255.4 Hz), 192.5 (C=O). Anal. calcd for C_21_H_13_FN_2_O 0.1 H_2_O: C 76.40, H 4.03, N 8.49; found: C 76.22, H 4.12, N 8.40.

*2-(3,4,5-Trimethoxybenzoyl)-3-(pyridin-4-yl)quinoline* (**19c**)

Yield 85% as a white solid. Mp 152–153 °C. ^1^H-NMR (400 MHz, CDCl_3_) ä 3.82 (s, 6H, OMe), 3.95 (s, 3H, OMe), 7.24 (s, 2H, Ar-H), 7.33–7.35 (m, 2H, pyridinyl-H), 7.69–7.73 (m, 1H, 7-H), 7.84 (d, 1H, *J* = 8.4 Hz, 6-H), 7.96–7.99 (m, 1H, 5-H), 8.21 (d, 1H, *J* = 8.4 Hz, 8-H), 8.32 (s, 1H, 4-H), 8.61–8.62 (m, 2H, pyridinyl-H). ^13^C-NMR (100 MHz, CDCl_3_) ä 56.3 (2C), 61.0, 108.3 (2C), 123.6, 127.8, 127.9, 128.6, 129.7, 130.8, 131.0, 131.8, 137.6 (2C), 143.5, 145.9, 146.3, 150.0 (2C), 153.0 (2C), 155.2, 192.9 (C=O). Anal. calcd for C_24_H_20_N_2_O_4_ 0.1 H_2_O: C 71.67, H 5.06, N 6.96; found: C 71.47, H 5.07, N 6.89.

#### 4.2.6. Preparation of 2-{1-[(4-Benzyloxy)phenyl]-1-hydroxylmethyl}-3-(3,4,5trimethoxyphenyl)- quinoline (**20**)

A mixture of **14** (1.0 mmol), [4-(benzyloxy)phenyl]magnesium bromide (3 mmol, 3 mL of a 1 M solution in THF), and THF (30 mL) was stirded at 0 °C for 12 h (TLC monitoring). The reaction was quenched by addition of water (3 mL) and partitioned between H_2_O (50 mL) and CH_2_Cl_2_ (50 mL). The organic layer was washed with brine, dried over MgSO_4_ and the solvent was removed in vacuo. The residue was purified by flash chromatography on silica gel (*n*-hexane/CH_2_Cl_2_ = 3/2) and recrystallized from EtOH to give compound **20**. Yield 54% as a yellow solid. Mp 121–122 °C. ^1^H-NMR (400 MHz, CDCl_3_) ä 3.66 (s, 6H, OMe), 3.90 (s, 3H, OMe), 4.96 (s, 2H, OCH_2_), 5.87 (s, 1H, 2-CH), 6.15 (s, 2H, Ar-H), 6.25 (br s, 1H, OH), 6.69–6.76 (m, 4H, Ar-H), 7.28–7.37 (m, 5H, Ar-H), 7.58–7.62 (m, 1H, 7-H), 7.77–7.85 (m, 2H, 5-H and 6-H), 7.96 (s, 1H, 4-H), 8.21 (d, 1H, *J* = 8.8 Hz, 8-H). ^13^C-NMR (100 MHz, CDCl_3_) ä 55.9 (2C), 61.0, 69.9, 72.5, 106.5 (2C), 114.4 (2C), 127.0, 127.3 (2C), 127.4, 127.5, 127.9, 128.5, 128.7, 129.1 (2C), 129.9, 133.2, 134.2, 135.2, 136.9, 137.1 (2C), 137.6, 145.2, 152.9 (2C), 158.1, 158.9. Anal. calcd for C_32_H_29_NO_5_ 0.2 H_2_O: C 75.19, H 5.80, N 2.74; found: C 74.90, H 5.82, N 2.72.

#### 4.2.7. Preparation of 2-(4-Hydroxyphenyl-1-hydroxylmethyl)-3-(3,4,5-trimethoxyphenyl)quinoline (**21**)

To a solution of **20** (1.0 mmol), in ethyl acetate (30 mL), Pd/C (100 mg) was added under hydrogen atmosphere and stirred at room temperature for 12 h (TLC monitoring). After completion of the reaction, filtered the reaction mass through the celite and washed with ethyl acetate (20 mL). The solvent was evaporated and the residue was purified by flash chromatography on silica gel (EtOAc/*n*-hexane = 1/2) as to give **21**. Yield 68% as a yellow solid. Mp 98–99 °C. ^1^H-NMR (400 MHz, CDCl_3_) ä 3.66 (s, 6H, OMe), 3.89 (s, 3H, OMe), 5.84 (s, 1H, 2-CH), 6.13 (s, 2H, Ar-H), 6.47 (d, 2H, *J* = 8.8 Hz, Ar-H), 6.61 (d, 2H, *J* = 8.4 Hz, Ar-H), 7.58–7.62 (m, 1H, 7-H), 7.77–7.85 (m, 2H, 5-H and 6-H), 7.96 (s, 1H, 4-H), 8.20 (d, 1H, *J* = 8.4 Hz, 8-H). ^13^C-NMR (100 MHz, CDCl_3_) ä 55.9 (2C), 61.0, 72.6, 106.2 (2C), 115.1 (2C), 127.0, 127.3, 127.5, 128.6, 129.3 (2C), 129.9, 133.2, 134.1, 134.1, 137.2, 137.4, 145.0, 152.9 (2C), 155.6, 158.7. Anal. calcd for C_25_H_23_NO_5_ 0.4 H_2_O: C 70.71, H 5.65, N 3.30; found: C 70.58, H 5.92, N 3.16.

#### 4.2.8. Preparation of 2-(4-Hydroxybenzoyl)-3-(3,4,5-trimethoxyphenyl)quinoline (**22**)

A mixture of **21** (1.0 mmol) and MnO_2_ (10 mmol) in CH_2_Cl_2_ (20 mL) was stirred at room temperature for 12 h (TLC monitoring). The reaction mixture was partitioned between H_2_O (50 mL) and CH_2_Cl_2_ (50 mL). The organic layer was washed with brine, dried over MgSO_4_ and the solvent was removed in vacuo. The residue was recrystallized from MeOH to give **22**. Yield 81% as a white solid. Mp 110–111 °C. ^1^H-NMR (400 MHz, DMSO) ä 3.59 (s, 6H, OMe), 3.65 (s, 3H, OMe), 6.71(s, 2H, Ar-H), 6.88 (d, 2H, *J* = 8.8 Hz, Ar-H), 7.72–7.76 (m, 3H, 7-H and Ar-H), 7.83–7.87 (m, 1H, 6-H), 8.07 (d, 1H, *J* = 8.4 Hz, 5-H), 8.13 (d, 1H, *J* = 7.6 Hz, 8-H), 8.63 (s, 1H, 4-H), 10.63 (br s, 1H, OH). ^13^C-NMR (100 MHz, DMSO) ä 55.5 (2C), 60.0, 106.3 (2C), 115.6 (2C), 127.6, 127.7, 127.9, 128.2, 128.7, 130.4, 132.7 (2C), 132.7, 132.8, 136.9, 137.1, 145.2, 152.8 (2C), 156.9, 162.9, 193.1 (C=O). Anal. calcd for C_25_H_21_NO_5_ 0.6 H_2_O: C 70.44, H 5.25, N 3.29; found: C 70.28, H 5.23, N 3.22.

#### 4.2.9. Preparation of 2-(4-Hydroxybenzoyl)-3-(pyridin-4-yl)quinoline (**23**)

A solution of **19a** (1.0 mmol) in 48% HBr (5 mL) was heated at reflux for 48 h (TLC monitoring). The mixture was cooled and evaporated in vacuo to give a residue, which was treated with H_2_O (50 mL). The precipitate was collected by suction filtration, washed with H_2_O and recrystallized from MeOH to give **23**. Yield 70% as a brown solid. Mp 256–257 °C. ^1^H-NMR (400 MHz, CDCl_3_) ä 6.87 (d, 2H, *J* = 8.4 Hz, Ar-H), 7.42–7.43 (m, 2H, pyridinyl-H), 7.71–7.80 (m, 3H, Ar-H and 7-H), 7.89–7.93 (m, 1H, 6-H), 8.11 (d, 1H, *J* = 8.4 Hz, 5-H), 8.18 (d, 1H, *J* = 7.6 Hz, 8-H), 8.58–8.60 (m, 2H, pyridinyl-H), 8.69 (s, 1H, 4-H), 10.67 (br s, 1H, OH). ^13^C-NMR (100 MHz, CDCl_3_) ä 115.6 (2C), 123.6, 127.3, 127.4, 128.3, 128.5, 128.8, 130.6, 131.1, 133.0 (2C), 138.0 (2C), 145.3, 145.6, 149.7 (2C), 155.9, 163.1, 192.5 (C=O). Anal. calcd for C_21_H_14_N_2_O_2_ 1.5 HBr 2.0 H_2_O: C 52.14, H 3.75, N 5.79; found: C 52.24, H 3.52, N 5.80.

### 4.3. Cell Viability and Anti-Inflammatory Activity Assays

#### 4.3.1. Reagents

RPMI-1640 medium, fetal bovine serum (FBS) and antibiotics were obtained from Gibco-BRL (Life Technologies, Grand Island, NY, USA). Dulbecco’s phosphate buffered saline (D-PBS), LPS (from *Escherichia coli* O111:B4), Griess reagent and 3-(4,5-dimethylthiazol-2-yl)-2, 5-diphenyl tetrazolium bromide (MTT), RIPA buffer, protease inhibitors and phosphatase inhibitors were obtained from Sigma Aldrich (St. Louis, MO, USA). TNF-α and IL-6 enzyme-linked immunosorbent assay (ELISA) kits were purchased from eBioscience (San Diego, CA, USA). BCA protein assay kit and ECL chemiluminescence substrate were obtained from Thermo Scientific (Waltham, MA, USA). Rabbit antibodies against mouse iNOS, COX-2 and â-actin and secondary antibodies were purchased from Santa Cruz Biotechnology (Santa Cruz, CA, USA). Rabbit antibodies against mouse phosphor-JNK, JNK, phosphor-ERK, ERK, phosphor-p38 MAPK, p38 MAPK were obtained from Cell Signaling (Farmingdale, NY, USA).

#### 4.3.2. Cell Culture

Murine macrophage cell line J774A.1 cells were obtained from Bioresource Collection and Research Center (Food Industry Research and Development Institute, Hsinchu, Taiwan), and were cultured in RPMI-1640 medium supplemented with 10% (*v*/*v*) FBS (Gibco-BRL, Life Technologies, Grand Island, NY, USA) and antibiotics (100 U/mL penicillin and 100 U/mL streptomycin). Cells were passaged every other day to maintain growth at 37 °C in a humidified incubator containing 5% CO_2_.

#### 4.3.3. NO Determination

J774A.1 cells were seeded in 96-well plates at a concentration of 1 × 10^6^ cells/mL. The cells were treated with 10 ìM 2-aroyl-3-arylquinoline derivatives for 1 h prior to treatment of LPS (1 ìg/mL). After 24 h, the cell culture supernatants were harvested and the concentration of NO was measured using the Griess reagent according to the manufacturer’s instructions (Sigma Aldrich).

#### 4.3.4. Cell Viability

J774A.1 cells were seeded in 96-well plates at a concentration of 1 × 10^6^ cells/mL. The cells were treated with 10 ìM 2-aroyl-3-arylquinoline derivatives for 1 h prior to treatment of LPS (1 ìg/mL). After 24 h, the cell culture supernatants were harvested. The cells were incubated in RPMI-1640 medium that containing 10% (*v*/*v*) MTT for 4 h at 37 °C, and then lysed with isopropanol. The cell viability was quantified by measuring the OD at 570 nm using a microplate reader.

#### 4.3.5. Cytokine Measurement

J774A.1 cells were seeded in 96-well plates at a concentration of 1 × 10^6^ cells/mL. The cells were treated with compounds **18a** and **18b** at different concentrations (0, 2.5, 5 and 10 ìM) for 1 h prior to treatment of LPS (1 ìg/mL) for 24 h. The cell culture supernatant was used for determination of TNF-α and IL-6 concentrations by ELISA according to the manufacturer’s protocols (eBioscience).

#### 4.3.6. NF-êB Promoter Reporter Assay

J-blue cell, a NF-êB reporter J774A.1 macrophage cell line, was obtained from Prof. Kuo-Feng Hua (National Ilan University, Taiwan) [16]. Cells were maintained in RPMI-1640 medium supplemented with Zeocin (200 ìg/mL) (InvivoGen, San Diego, CA, USA). A total of 1 × 10^6^ cells were seeded in a 96-well plate and grown overnight in a 5% CO_2_ incubator at 37 °C. And then, cells were pre-treated with various concentrations of compounds **18a** and **18b** (0, 2.5, 5 and 10 ìM) for 1 h prior to treatment of LPS (1 ìg/mL) for 24 h. The culture medium was harvested and mixed with QUANTI-Blue medium in a 1:10 ratio (20 ìL cell culture supernatant to 200 ìL QUANTI-Blue medium) (InvivoGen, San Diego, CA, USA) in 96-well plates and incubated at 37 °C for 45 min. The activity of SEAP was examined by analyzing the optical density at 655 nm using a microplate reader.

#### 4.3.7. Western Blot

Cells were lysed by RIPA buffer containing protease inhibitors and phosphatase inhibitors, and the concentration of protein was measured by BCA protein assay kit according to the manufacturer’s instructions (Thermo Scientific). Equal amounts of cellular protein extracts were separated by SDS-PAGE. Afterwards, proteins were electrophoretically transferred to PVDF membranes. The membranes were incubated with blocking solution for 1 h at room temperature, followed by incubation overnight with primary antibodies at 4 °C. After washing three times with Tris-buffered saline/Tween 20 (TBST), the blots were hybridized with secondary antibody conjugated with horseradish peroxidase for 1 h at room temperature. Thereafter, blots were washed three times with TBST and developed using an ECL chemiluminescence substrate (Thermo Scientific). The signals were captured using Bio-Rad ChemiDOC XRS^+^ system (Bio-Rad Laboratories, Inc., Hercules, CA, USA).

#### 4.3.8. Molecular Docking Study

The crystal structure of TNF-á (PDB ID: 2AZ5) was acquired from the RCSB Protein Date Bank. The 3D conformation of target compound **18a** and **18b** was produced by ChemBio 3D Ultra 14.0. The molecular docking was performed by Achilles Blind Docking Server (http://bio-hpc.ucam.edu/achilles/). The “blind docking” approach was used for the docking of the small molecule to the targets, which was done without a priori knowledge of the location of the binding site by the system [39]. Visual representation of molecules was created with 3Dmol by Nicholas Rego and David Koes [40].

#### 4.3.9. Statistical Analysis

All data are presented as means ± SD. Each value is the mean of three independent experiments. Statistical analysis was assessed via one-way ANOVA followed by Tukey post-hoc test by GraphPad Prism 5 (San Diego, CA, USA), and the significant difference was set at *: *p* < 0.05; **: *p* < 0.01.

## 5. Conclusions

We have synthesized a total of sixteen 2-substituted 3-arylquinoline derivatives and evaluated their anti-inflammatory effect in LPS-activated murine macrophage cell line J774A.1 cells. Among these 2-substituted 3-arylquinoline derivatives, we found that compounds **18a** and **18b** have significant anti-inflammatory activities on LPS-activated macrophages through inhibition of the production of NO, TNF-α and IL-6 and attenuating the activity of NF-êB, repressing the expression of iNOS, and suppressing the phosphorylation of MAPKs. Compounds **18a** and **18b** might have potential starting points for the development of anti-inflammatory and immunosuppressive drugs in the treatment of sepsis and septic shock.

## Figures and Tables

**Table 1:** Effects of 2-substituted 3-arylquinoline derivatives on NO production by LPS-activated J774A.1 cells.

Compounds	Nitric Oxide (μM)
Control	1.17 ± 0.30
LPS	15.43 ± 0.58
**17c**	12.00 ± 2.67
**17b**	11.55 ± 0.30
**19c**	9.70 ± 2.46
**19b**	11.34 ± 1.99
**18c**	10.40 ± 1.29
**16a**	10.90 ± 0.56
**18a**	6.70 ± 2.62
**16b**	10.64 ± 1.01
**18b**	7.41 ± 0.34
**19a**	10.16 ± 1.67
**17a**	11.5 ± 0.43
**16c**	12.47 ± 0.97
**23**	12.59 ± 0.35
**22**	8.52 ± 1.57
**20**	8.83 ± 3.20
**21**	10.31 ± 0.72

## Supplementary Materials

The following are available online at https://www.mdpi.com/1420-3049/24/6/1162/s1.

## References

[1] Wynn T.A., Chawla A., Pollard J.W. (2013). Macrophage biology in development, homeostasis and disease. Nature.

[2] Murray P.J., Wynn T.A. (2011). Protective and pathogenic functions of macrophage subsets. Nat. Rev. Immunol..

[3] Guha M., Mackman N. (2001). LPS induction of gene expression in human monocytes. Cell. Signal..

[4] Hung Y.L., Fang S.H., Wang S.C., Cheng W.C., Liu P.L., Su C.C., Chen C.S., Huang M.Y., Hua K.F., Shen K.H. (2017). Corylin protects LPS-induced sepsis and attenuates LPS-induced inflammatory response. Sci. Rep..

[5] Raymond S.L., Holden D.C., Mira J.C., Stortz J.A., Loftus T.J., Mohr A.M., Moldawer L.L., Moore F.A., Larson S.D., Efron P.A. (2017). Microbial recognition and danger signals in sepsis and trauma. Biochim. Biophys. Acta.

[6] Boulange C.L., Neves A.L., Chilloux J., Nicholson J.K., Dumas M.E. (2016). Impact of the gut microbiota on inflammation, obesity, and metabolic disease. Genome Med..

[7] Medzhitov R. (2008). Origin and physiological roles of inflammation. Nature.

[8] Szabo C., Modis K. (2010). Pathophysiological roles of peroxynitrite in circulatory shock. Shock.

[9] Chousterman B.G., Swirski F.K., Weber G.F. (2017). Cytokine storm and sepsis disease pathogenesis. Semin. Immunopathol..

[10] Dandona P., Aljada A., Bandyopadhyay A. (2004). Inflammation: the link between insulin resistance, obesity and diabetes. Trends Immunol..

[11] El-Feky S.A.H., Abd El-Samii Z.K., Osman N.A., Lashine J., Kamel M.A., Thabet H.K. (2015). Synthesis, molecular docking and anti-inflammatory screening of novel quinoline incorporated pyrazole derivatives using the Pfitzinger reaction II. Bioorg. Chem..

[12] Ghodsi R., Zarghi A., Daraei B., Hedayati M. (2010). Design, synthesis and biological evaluation of new 2,3-diarylquinoline derivatives as selective cyclooxygenase-2 inhibitors. Bioorg. Med. Chem..

[13] Suthar S.K., Jaiswal V., Lohan S., Bansal S., Chaudhary A., Tiwari A., Alex A.T., Joesph A. (2013). Novel quinolone substituted thiazolidin-4-ones as anti-inflammatory, anticancer agents: design, synthesis and biological screening. Eur. J. Med. Chem..

[14] Chaaban I., Rizk O.H., Ibrahim T.M., Henen S.S., El-Khawass E.M., Bayad A.E., El-Ashmawy I.M., Nematalla H.A. (2018). Synthesis, anti-inflammatory screening, molecular docking, and COX-1,2/-5-LOX inhibition profile of some novel quinoline derivatives. Bioorg. Chem..

[15] Zarghi A., Ghodsi R. (2010). Design, synthesis, and biological evaluation of ketoprofen analogs as potent cyclooxygenase-2 inhibitors. Bioorg. Med. Chem..

[16] Mazzoni O., Esposito G., Diurno M.V., Brancaccio D., Carotenuto A., Grieco P., Novellino E., Filippelli W. (2010). Synthesis and pharmacological evaluation of some 4-oxo-quinoline-2-carboxylic acid derivatives as anti-inflammatory and analgesic agents. Arch. Pharm. Chem. Life Sci..

[17] Tseng C.H., Lin C.S., Shih P.K., Tsao L.T., Wang J.P., Cheng C.M., Tzeng C.C., Chen Y.L. (2009). Furo[3, 2:3,4]naphtho[1,2-*d*]imidazole derivatives as potential inhibitors of inflammatory factors in sepsis. Bioorg. Med. Chem..

[18] Tseng C.H., Tzeng C.C., Shih P.K., Yang C.N., Chuang Y.C., Peng S.I., Lin C.S., Wang J.P., Cheng C.M., Chen Y.L. (2012). Identification of furo[3’,2’:3,4]naphtho[1,2-*d*]imidazole derivatives as orally active and selective inhibitors of microsomal prostaglandin E(2) synthase-1 (mPGES-1). Mol. Divers..

[19] Chen Y.R., Tseng C.H., Chen Y.L., Hwang T.L., Tzeng C.C. (2015). Discovery of benzo[*f*]indole-4,9-dione derivatives as new type of anti-inflammatory agents. Int. J. Mol. Sci..

[20] Tseng C.H., Tung C.W., Wu C.H., Tzeng C.C., Chen Y.H., Hwang T.L., Chen Y.L. (2017). Discovery of indeno[1,2-*c*]quinoline derivatives as potent dual antituberculosis and anti-Inflammatory agents. Molecules.

[21] Tseng C.H., Tung C.W., Peng S.I., Chen Y.L., Tzeng C.C., Cheng C.M. (2018). Discovery of pyrazolo[4,3-*c*]quinolines derivatives as potential anti-inflammatory agents through inhibiting of NO production. Molecules.

[22] Tseng C.H., Lin C.K., Chen Y.L., Hsu C.Y., Wu H.N., Tseng C.K., Lee J.C. (2014). Synthesis, antiproliferative and anti-dengue virus evaluations of 2-aroyl-3-arylquinoline derivatives. Eur. J. Med. Chem..

[23] Tseng C.H., Chen Y.L., Hsu C.Y., Chen T.C., Cheng C.M., Tso H.C., Lu Y.J., Tzeng C.C. (2013). Synthesis and antiproliferative evaluation of 3-phenylquinolinylchalcone derivatives against non-small cell lung cancers and breast cancers. Eur. J. Med. Chem..

[24] Palmer M.H., McIntyre P.S. (1969). The Pfitzinger reaction with unsymmetrical ketones. J. Chem. Soc. B.

[25] Wallace J.L. (2005). Nitric oxide as a regulator of inflammatory processes. Mem. Inst. Oswaldo. Cruz..

[26] Tsikas D. (2007). Analysis of nitrite and nitrate in biological fluids by assays based on the Griess reaction: appraisal of the Griess reaction in the L-arginine/nitric oxide area of research. J. Chromatogr. B Analyt. Technol. Biomed. Life Sci..

[27] Tanaka T., Narazaki M., Kishimoto T. (2014). IL-6 in inflammation, immunity, and disease. Cold Spring Harb Perspect. Biol..

[28] Turner M.D., Nedjai B., Hurst T., Pennington D.J. (2014). Cytokines and chemokines: At the crossroads of cell signalling and inflammatory disease. Biochim. Biophys. Acta.

[29] Liu T., Zhang L., Joo D., Sun S.C. (2017). NF-kappaB signaling in inflammation. Signal. Transduct. Target Ther..

[30] Hua K.F., Yang F.L., Chiu H.W., Chou J.C., Dong W.C., Lin C.N., Lin C.Y., Wang J.T., Li L.H., Chiu H.W. (2015). Capsular Polysaccharide Is Involved in NLRP3 Inflammasome Activation by Klebsiella pneumoniae Serotype K1. Infect. Immun..

[31] Singh P., Kaur S., Sharma A., Kaur G., Bhatti R. (2017). TNF-α and IL-6 inhibitors: Conjugates of N-substituted indole and aminophenylmorpholin-3-one as anti-inflammatory agents. Eur. J. Med. Chem..

[32] Tang K.W., Lin Z.C., Chen Y.L., Tzeng C.C., Fang J.Y., Tseng C.H. (2018). Synthesis and biological evaluation of thalidomide derivatives as potential anti-psoriasis agents. Int. J. Mol. Sci..

[33] Riera Romo M., Perez-Martinez D., Castillo Ferrer C. (2016). Innate immunity in vertebrates: An overview. Immunology.

[34] Moore J.X., Donnelly J.P., Griffin R., Howard G., Safford M.M., Wang H.E. (2016). Defining sepsis mortality clusters in the United States. Crit. Care Med..

[35] Zamora R., Vodovotz Y., Billiar T.R. (2000). Inducible nitric oxide synthase and inflammatory diseases. Mol. Med..

[36] Schulte W., Bernhagen J., Bucala R. (2013). Cytokines in sepsis: Potent immunoregulators and potential therapeutic targets--an updated view. Mediat. Inflamm..

[37] Hotamisligil G.S. (2006). Inflammation and metabolic disorders. Nature.

[38] He M.M., Smith A.S., Oslob J.D., Flanagan W.M., Braisted A.C., Whitty A., Cancilla M.T., Wang J., Lugovskoy A.A., Yoburn J.C. (2005). Small-molecule inhibition of TNF-α. Science.

[39] Sánchez-Linares I., Pérez-Sánchez H., Cecilia J.M., García J.M. (2012). High-throughput parallel blind virtual screening using BINDSURF. BMC Bioinform..

[40] Rego N., Koes D. (2015). 3Dmol.js: Molecular visualization with WebGL. Bioinformatics.

