# Supplementary material for: Discovery of 2-Substituted 3-Arylquinoline Derivatives as Potential Anti-Inflammatory Agents Through Inhibition of LPS-Induced Inflammatory Responses in Macrophages
Source: Molecules. 2019 Mar 23;24(6):1162. doi: 10.3390/molecules24061162 (PMC6472047; doi:10.3390/molecules24061162)

YCY-6301

Pulse Sequence: s2pul
Mercury-400BB "MerPlus400"
Date: Dec 20 2018
Solvent: dmso
Ambient temperature
Total 32 repetitions

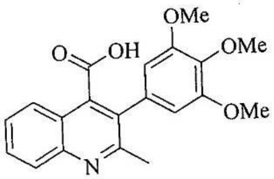

10

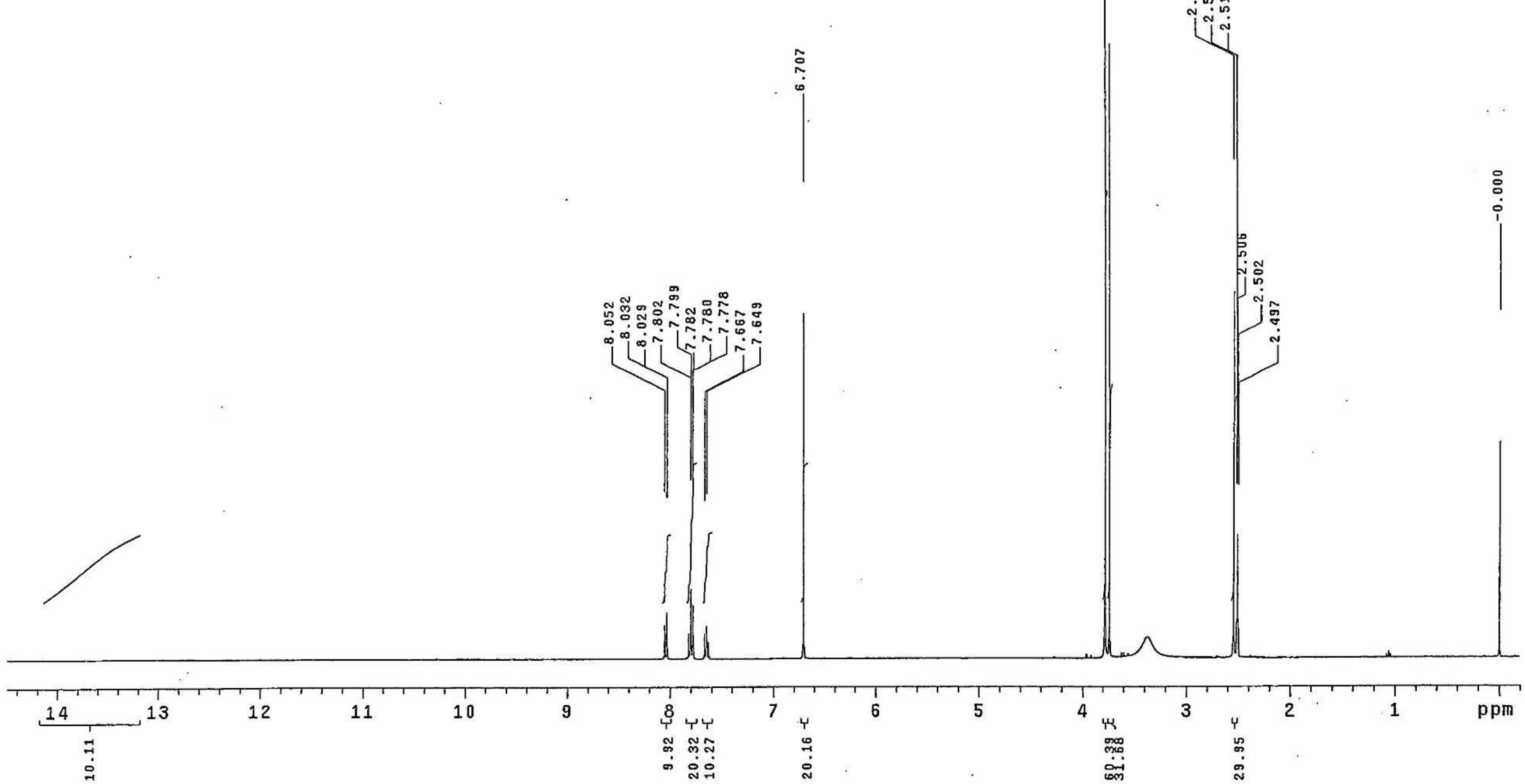

YCY-6301

Pulse Sequence: s2pul
Mercury-400BB "MerPlus400"
Date: Dec 20 2018
Solvent: dmso
Ambient temperature
Total 3312 repetitions

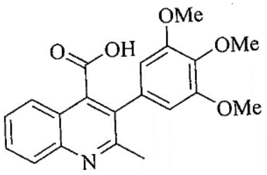

10

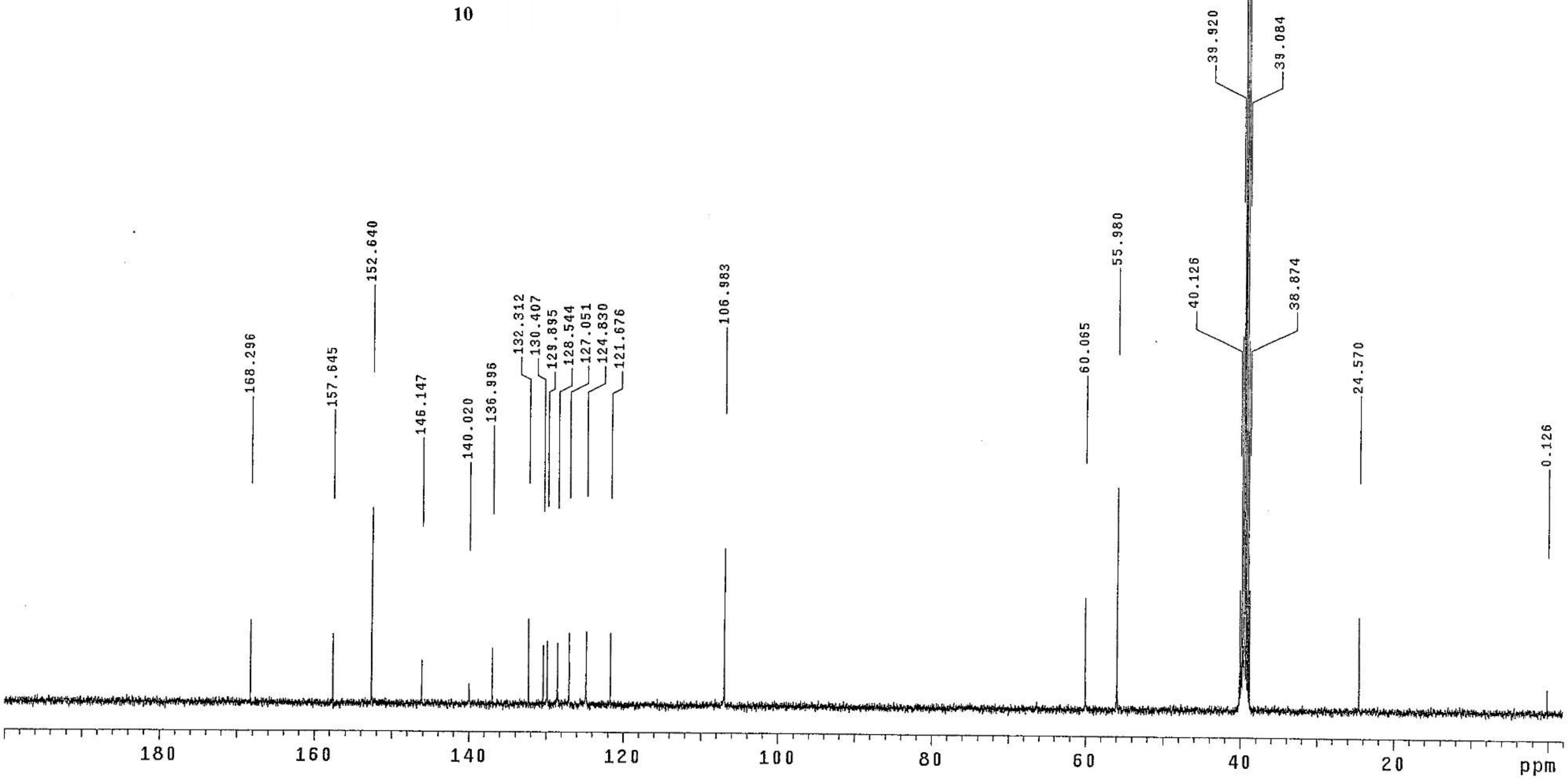

3-pyr-Cl
Pulse Sequence: s2pul
INOVA-400 "unityplus400"
Date: Dec 22 2010
Solvent: DMSO
Ambient temperature
Total 64 repetitions

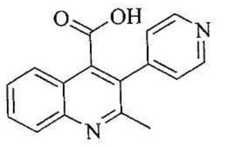

11

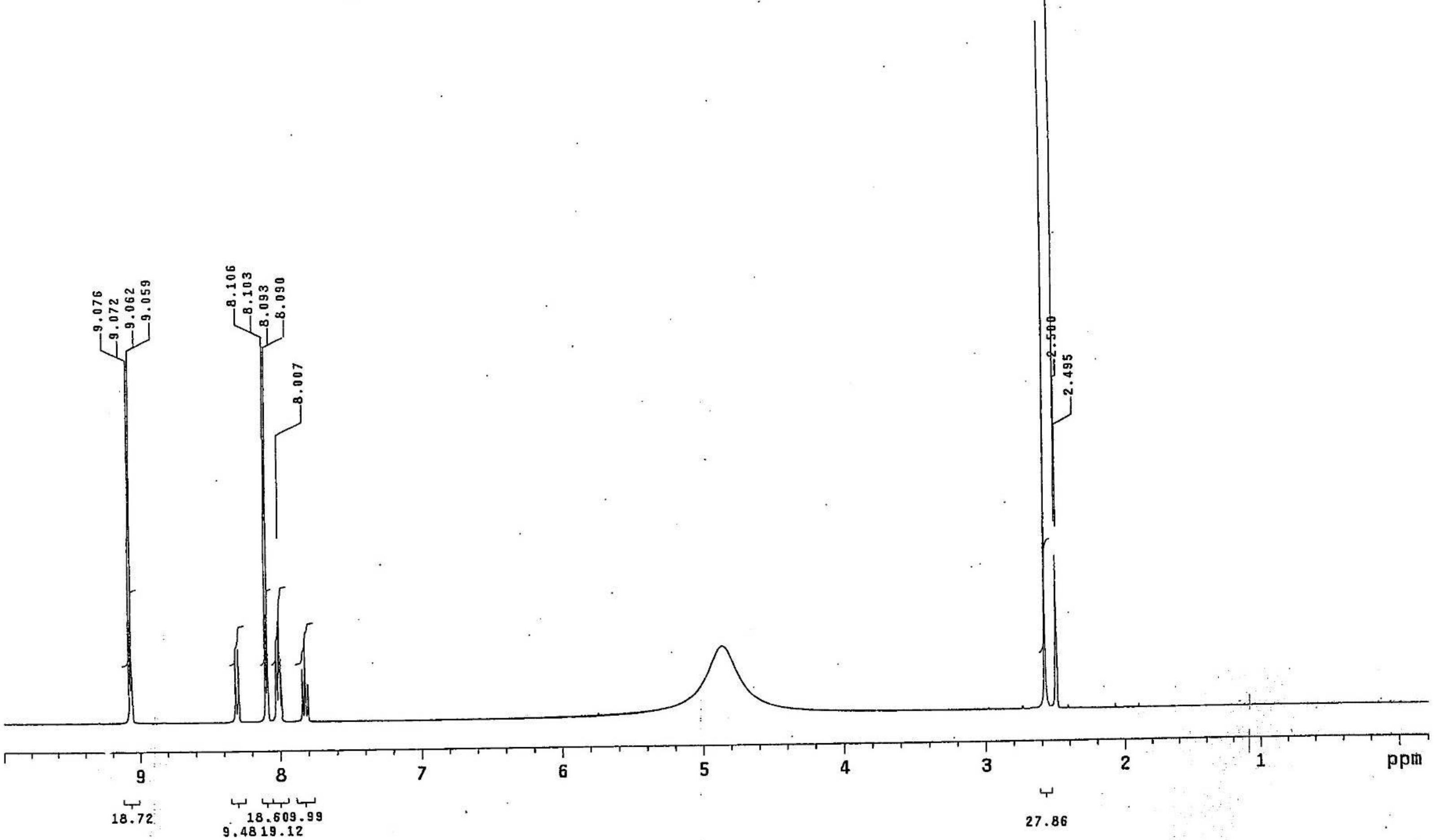

3-pyr-Cl

Pulse Sequence: s2pul

INOVA-400 "unityplus400"
Date: Dec 22 2010
Solvent: DMSO
Ambient temperature
Total 7216 repetitions

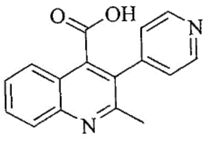

11

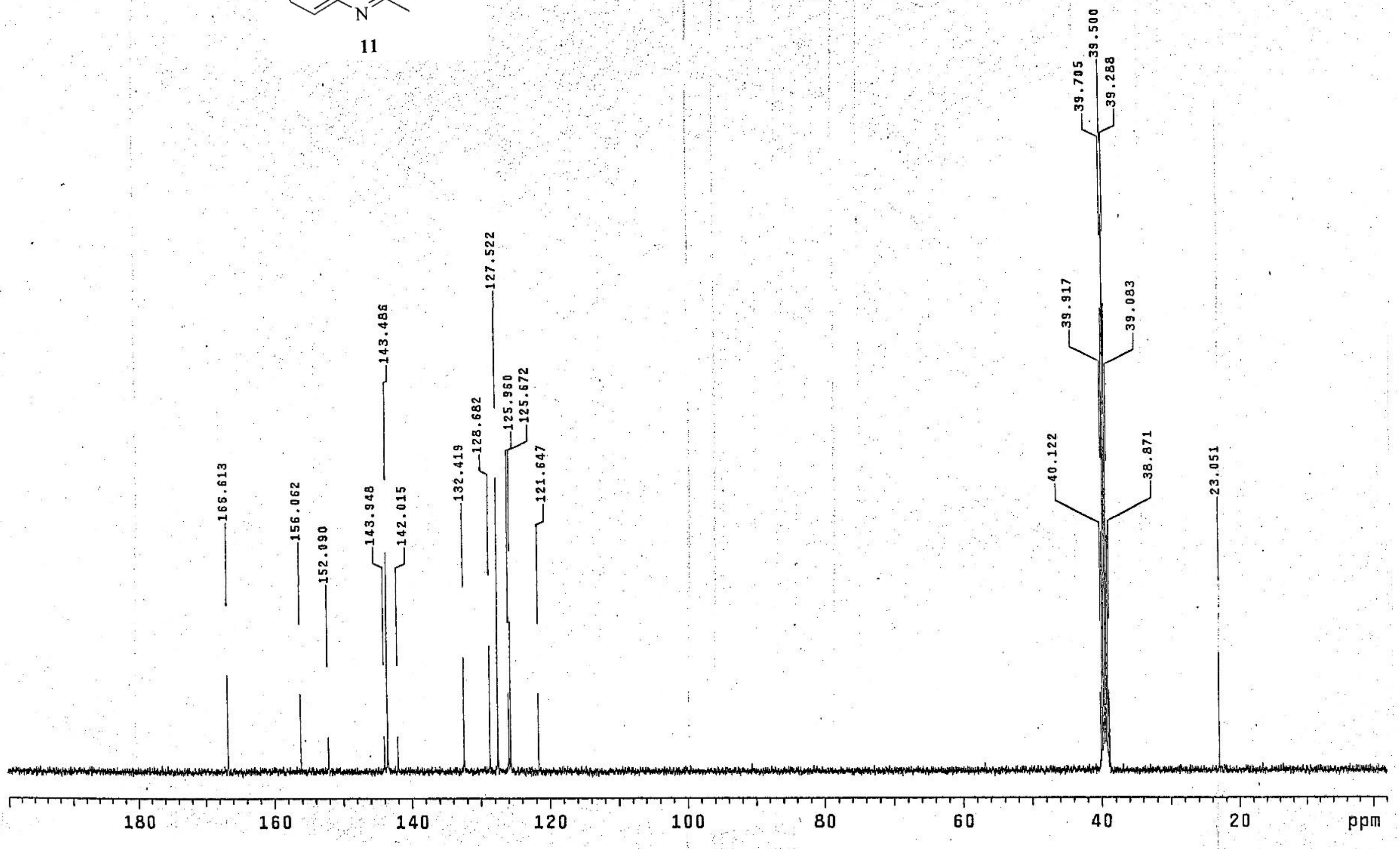

YCY-6302

Pulse Sequence: s2pul
Mercury-400BB "MerPlus400"
Date: Dec 20 2018
Solvent: cdcl3
Ambient temperature
Total 32 repetitions

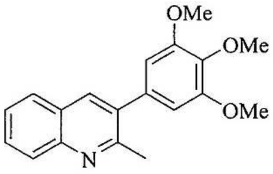

12

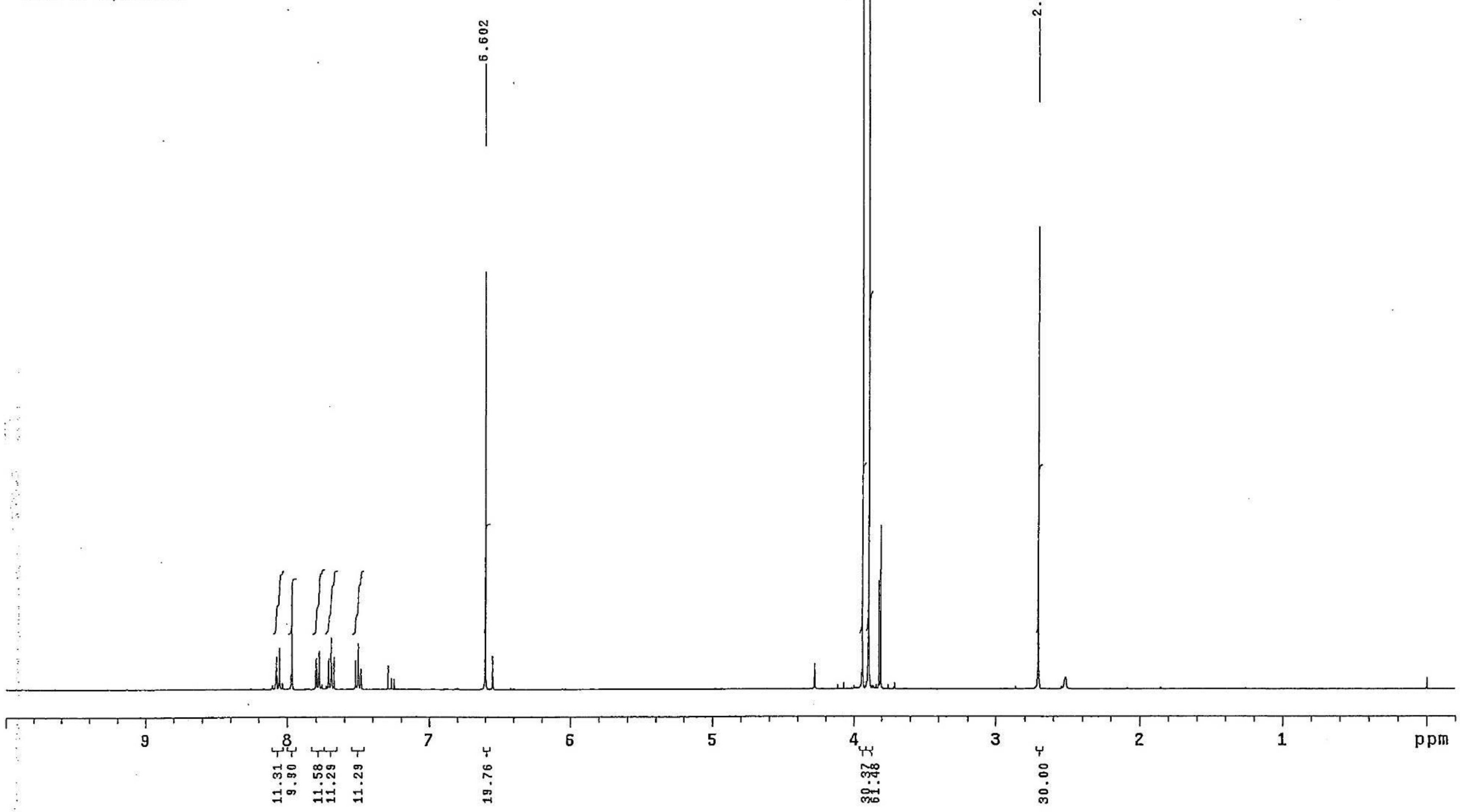

YCY-6302

Pulse Sequence: s2pul
Mercury-400BB "MerPlus400"
Date: Dec 20 2018
Solvent: cdcl3
Ambient temperature
Total 1328 repetitions

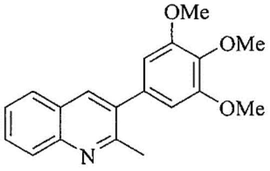

12

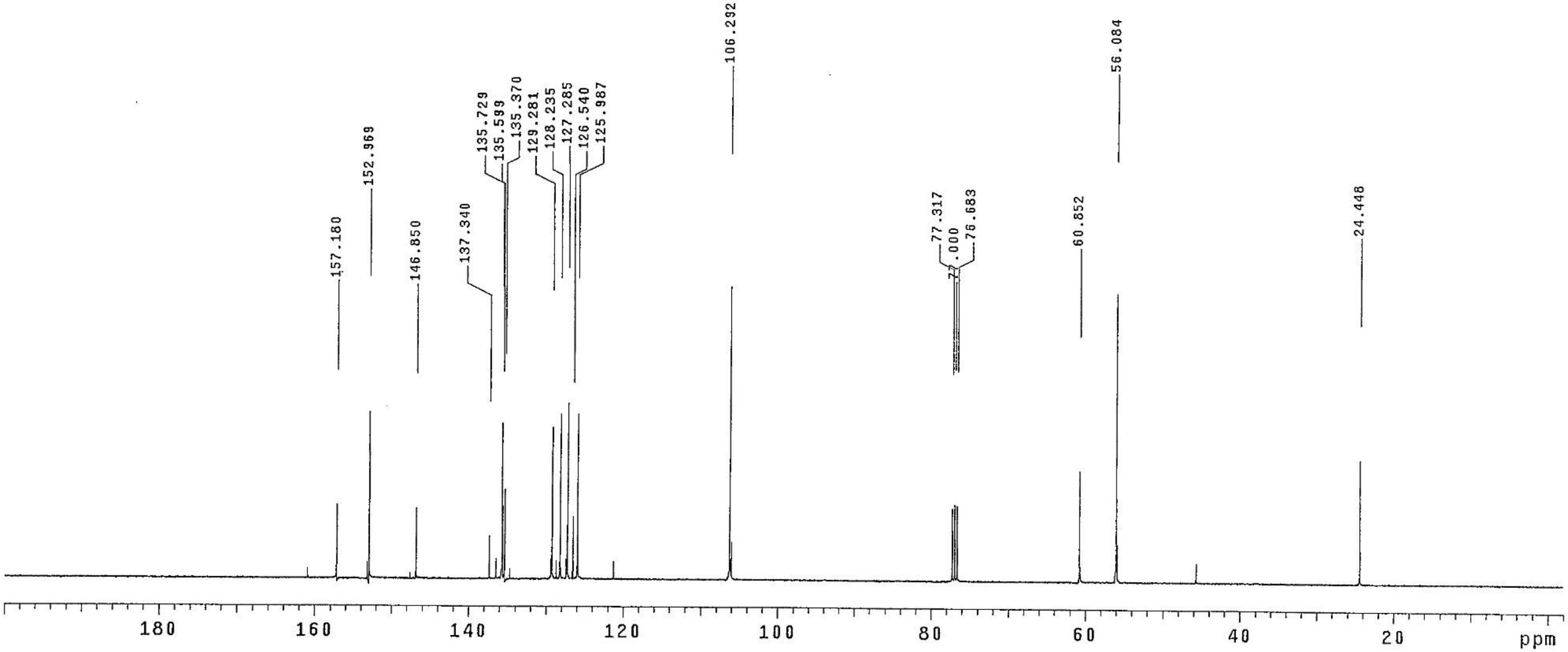

STANDARD 1H OBSERVE
3- pyri- dec1
Pulse Sequence: s2pul
INOVA-400 "unityplus400"
Date: Dec 29 2010
Solvent: CDCl3
Ambient temperature
Total 64 repetitions

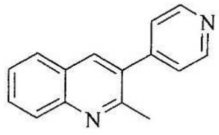

13

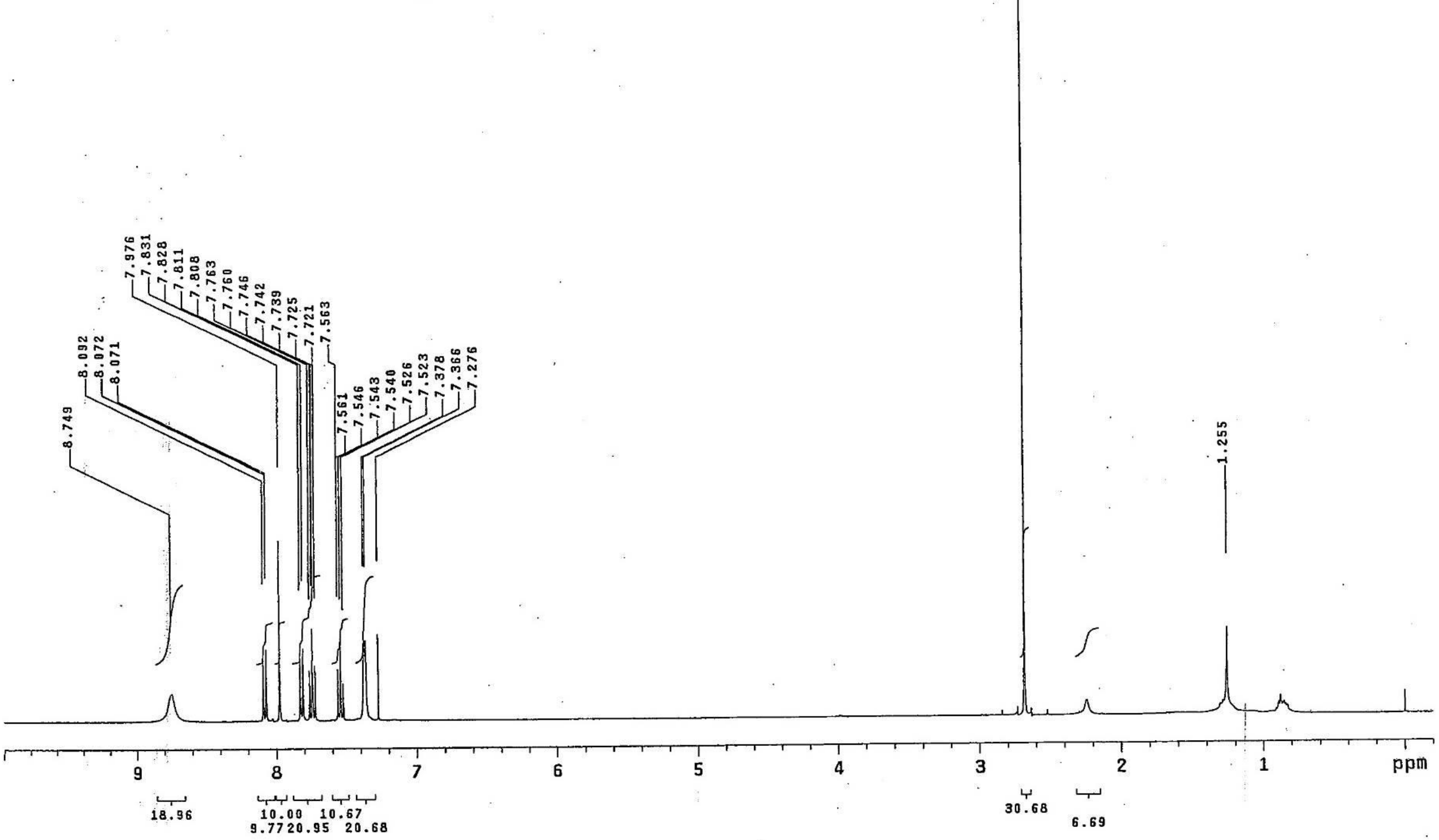

3-pyri-decl

Pulse Sequence: s2pul
INOVA-400 "unityplus400"
Date: Dec 29 2010
Solvent: CDCl3
Ambient temperature
Total 32000 repetitions

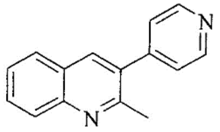

13

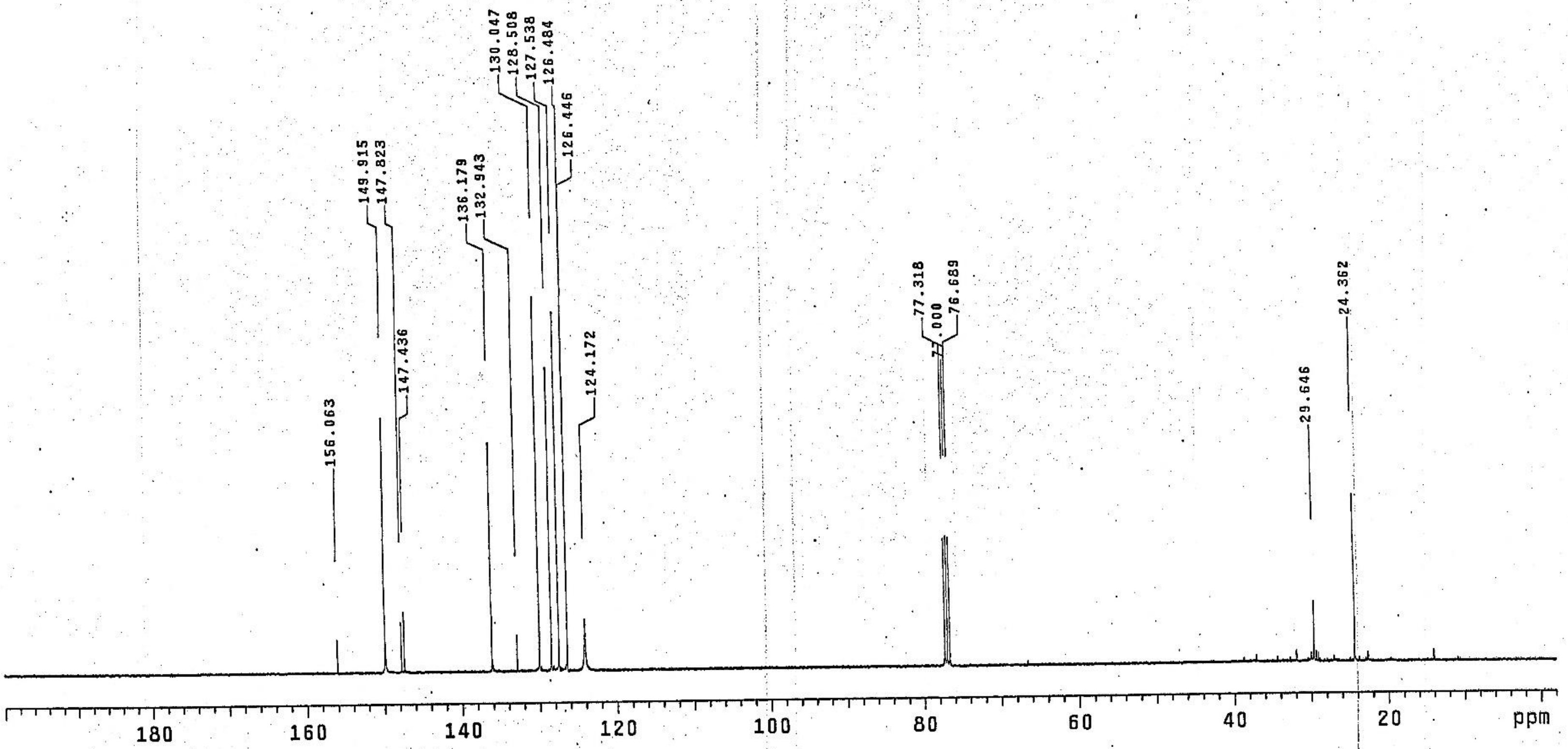

YCY-6303

Pulse Sequence: s2pul
Mercury-400BB "MerPlus400"
Date: Dec 20 2018
Solvent: cdcl3
Ambient temperature
Total 32 repetitions

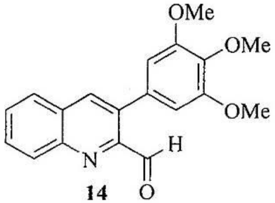

14

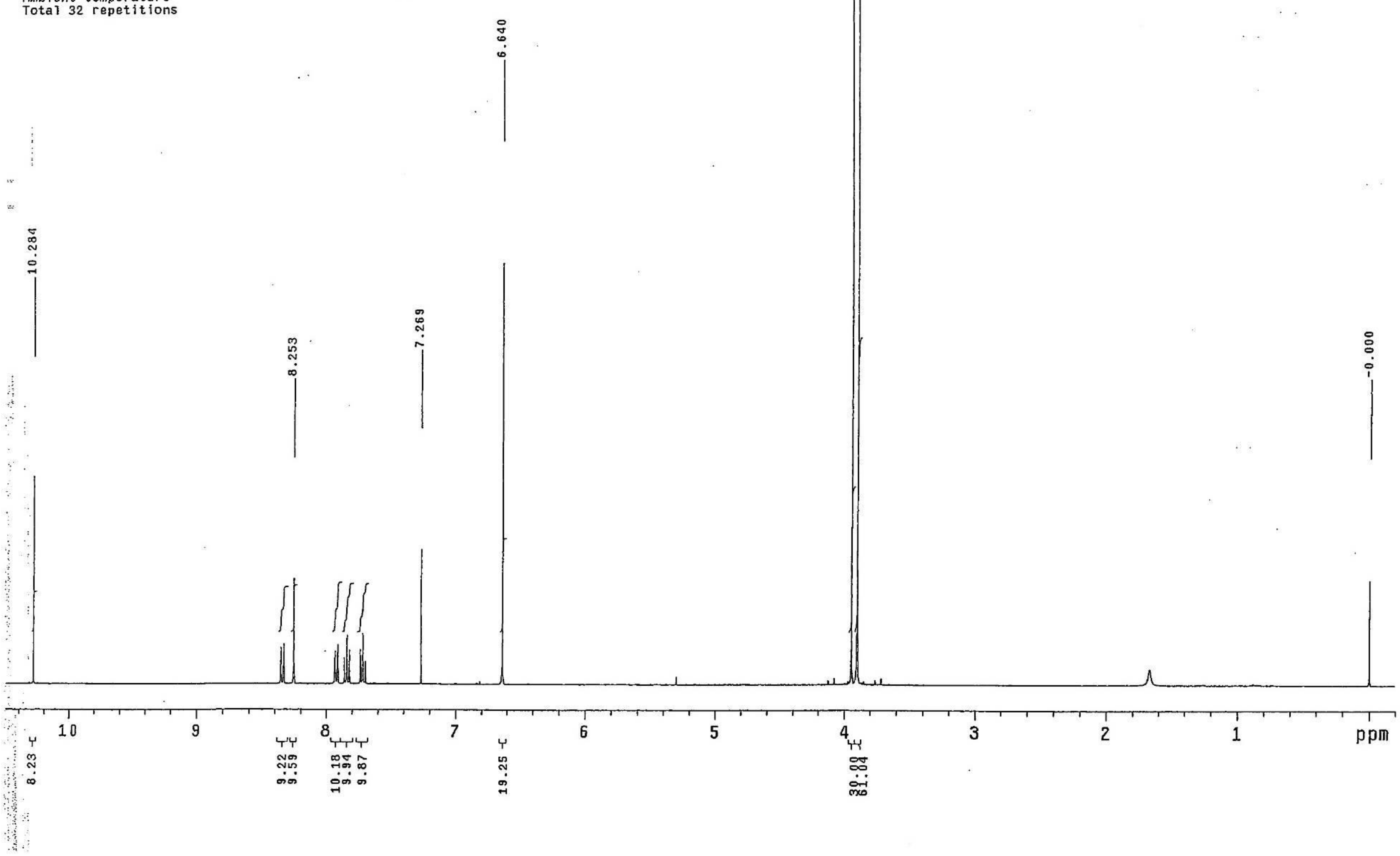

YCY-6303

Pulse Sequence: s2pul
Mercury-400BB "MerPlus400"
Date: Dec 20 2018
Solvent: cdcl3
Ambient temperature
Total 2800 repetitions

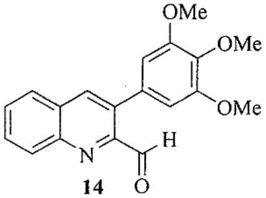

14

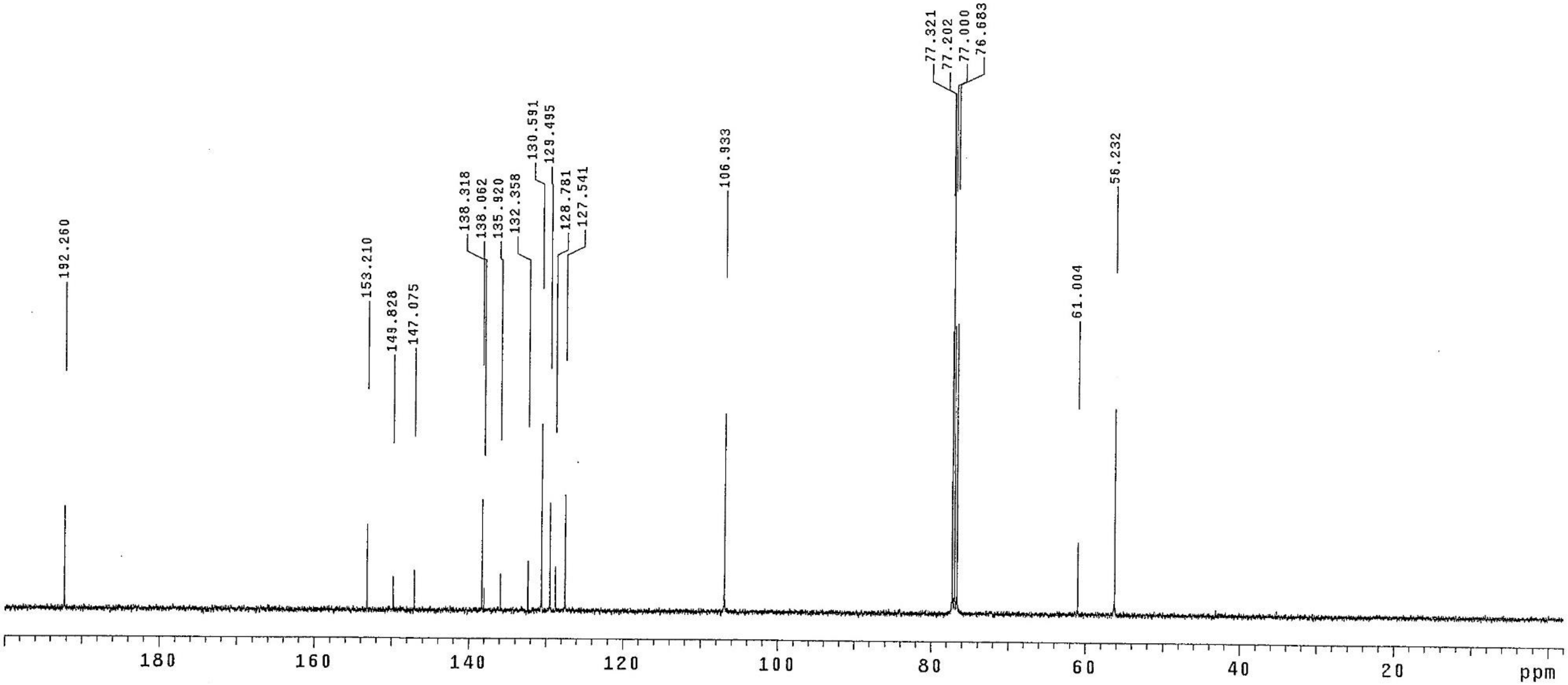

3-pyri-de-COH

Pulse Sequence: s2pul
INOVA-400 "unityplus400"
Date: Jan 12 2011
Solvent: CDCl3
Ambient temperature
Total 64 repetitions

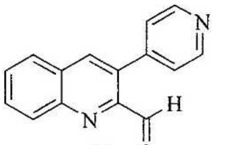

15

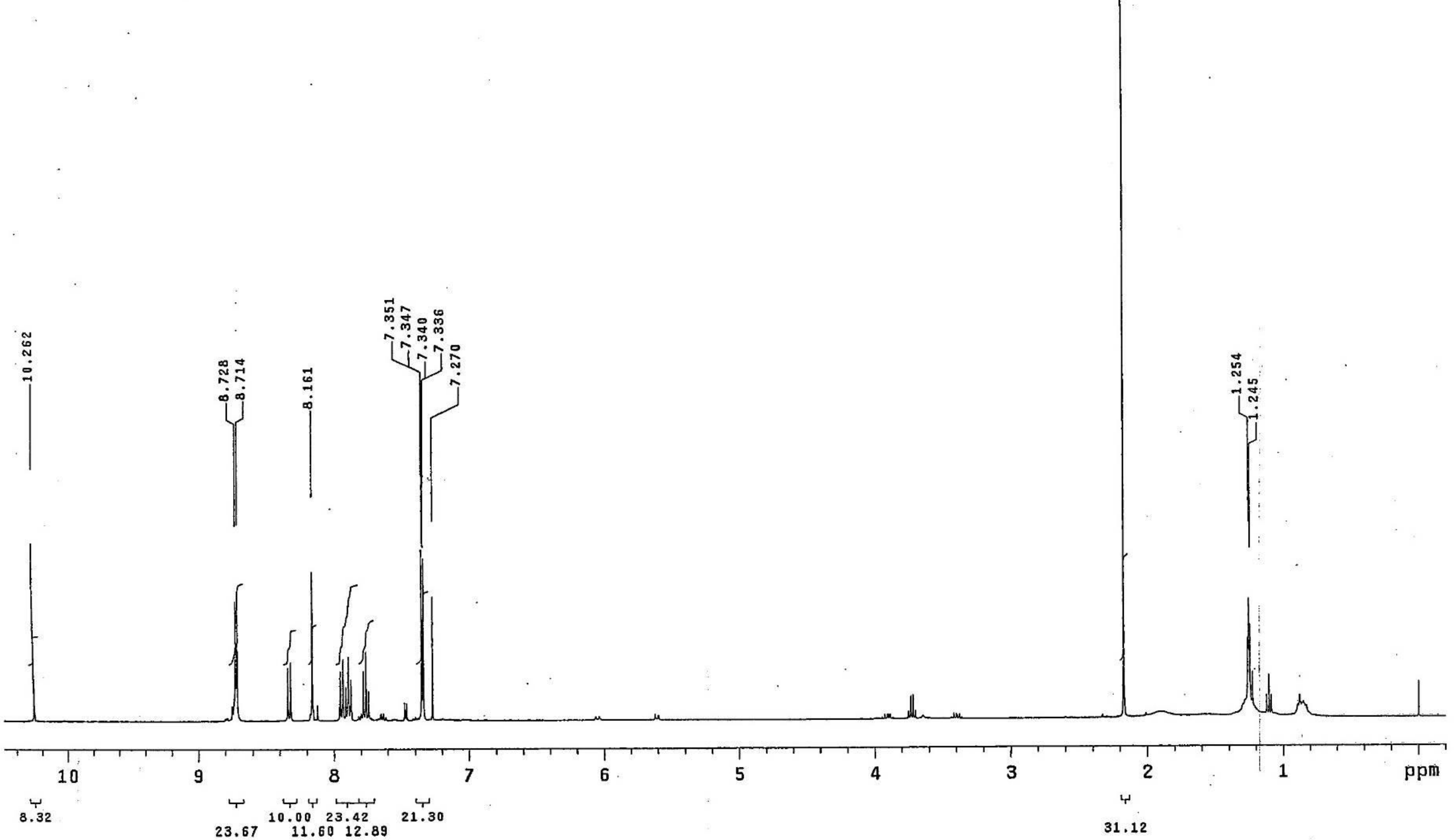

3-pyri-de-COH

Pulse Sequence: s2pul
INOVA-400 "unityplus400"
Date: Jan 12 2011
Solvent: CDCl3
Ambient temperature
Total 3392 repetitions

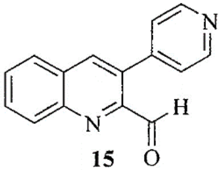

15

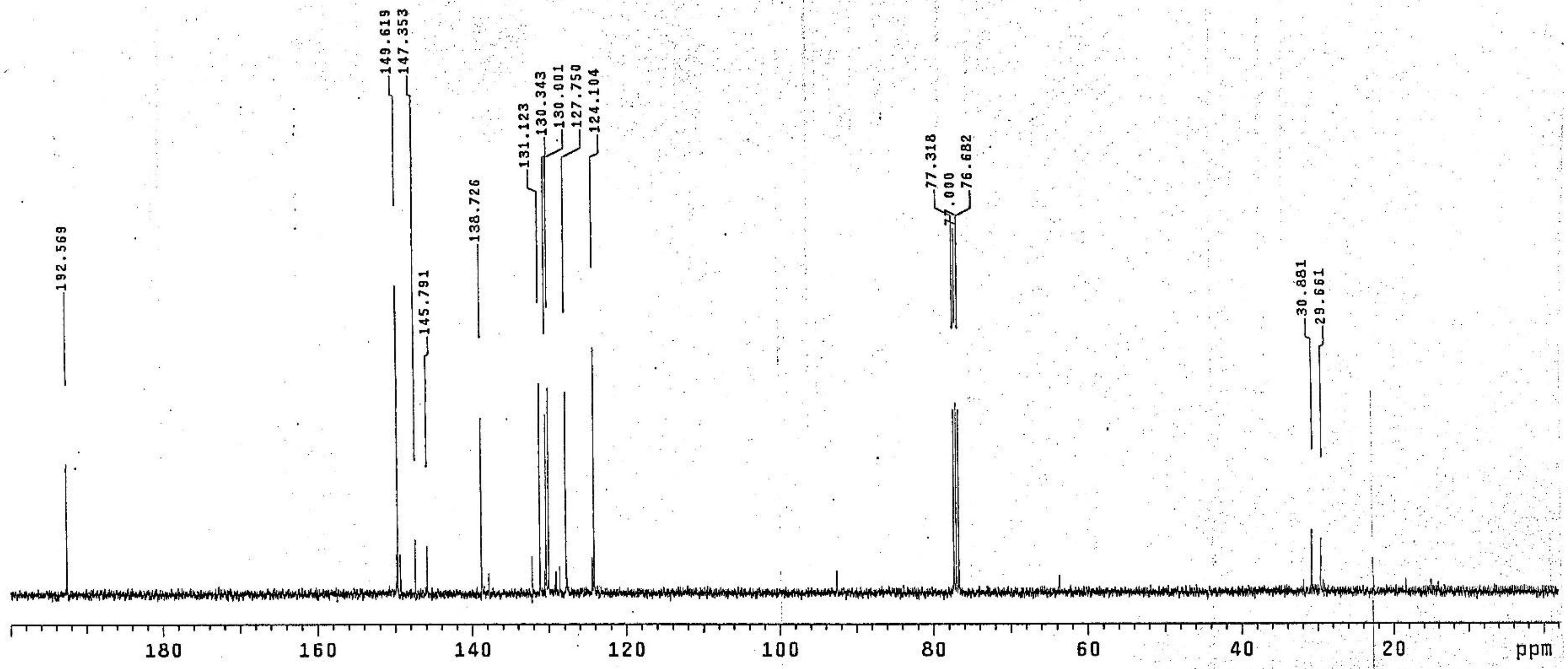

STANDARD 1H OBSERVE
YCY-[illegible]
Pulse Sequence: s2pul
UNITYplus-400 "unity400"
Date: Jul 30 2012
Solvent: CDCl3
Ambient temperature
Total 80 repetitions

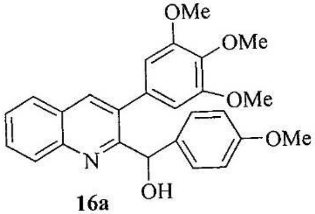

16a

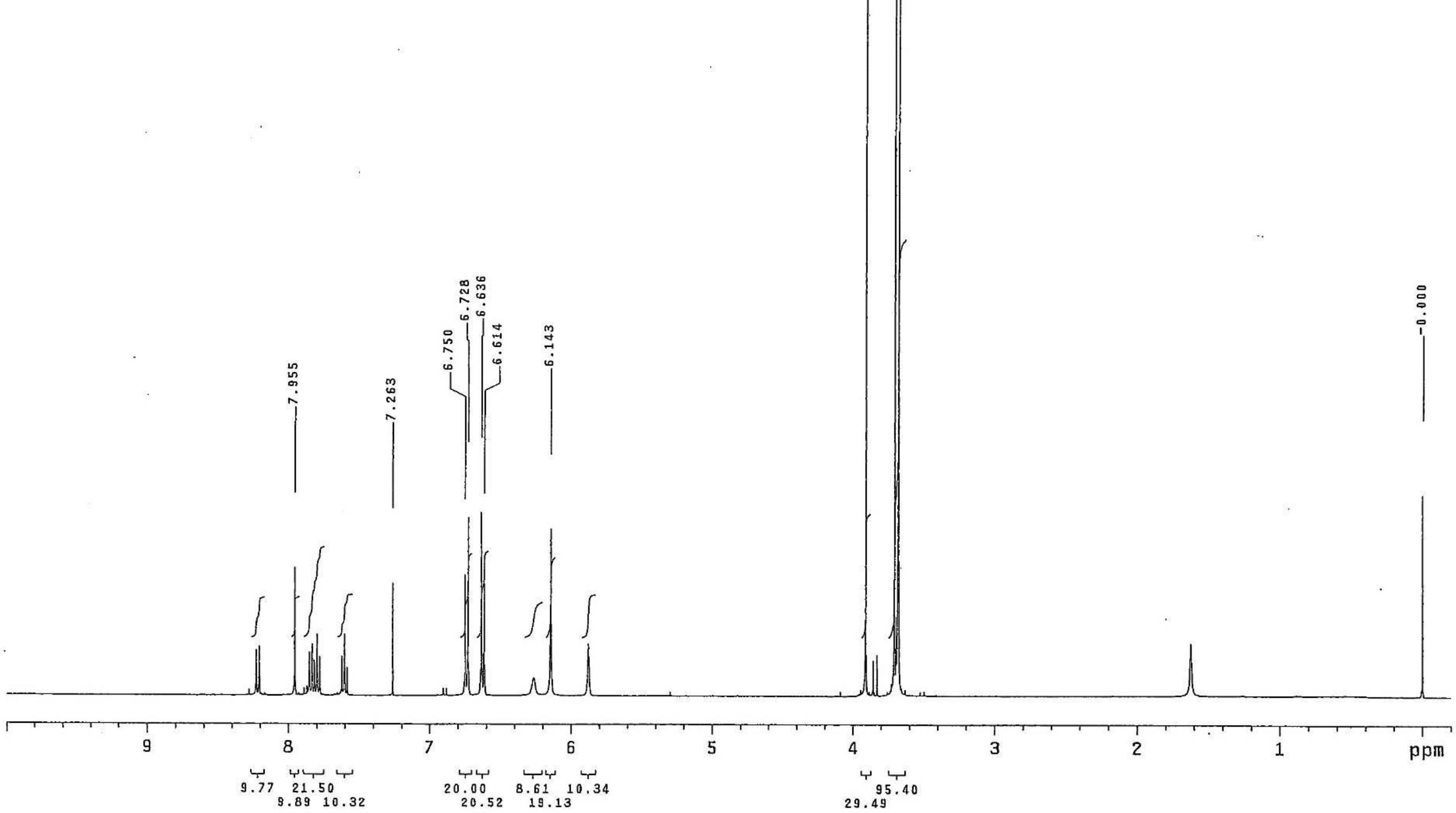

YCY-4856

Pulse Sequence: s2pul
UNITYplus-400 "unity400"
Date: Jul 30 2012
Solvent: DMSO
Ambient temperature
Total 7392 repetitions

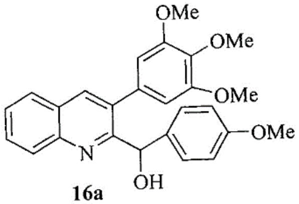

16a

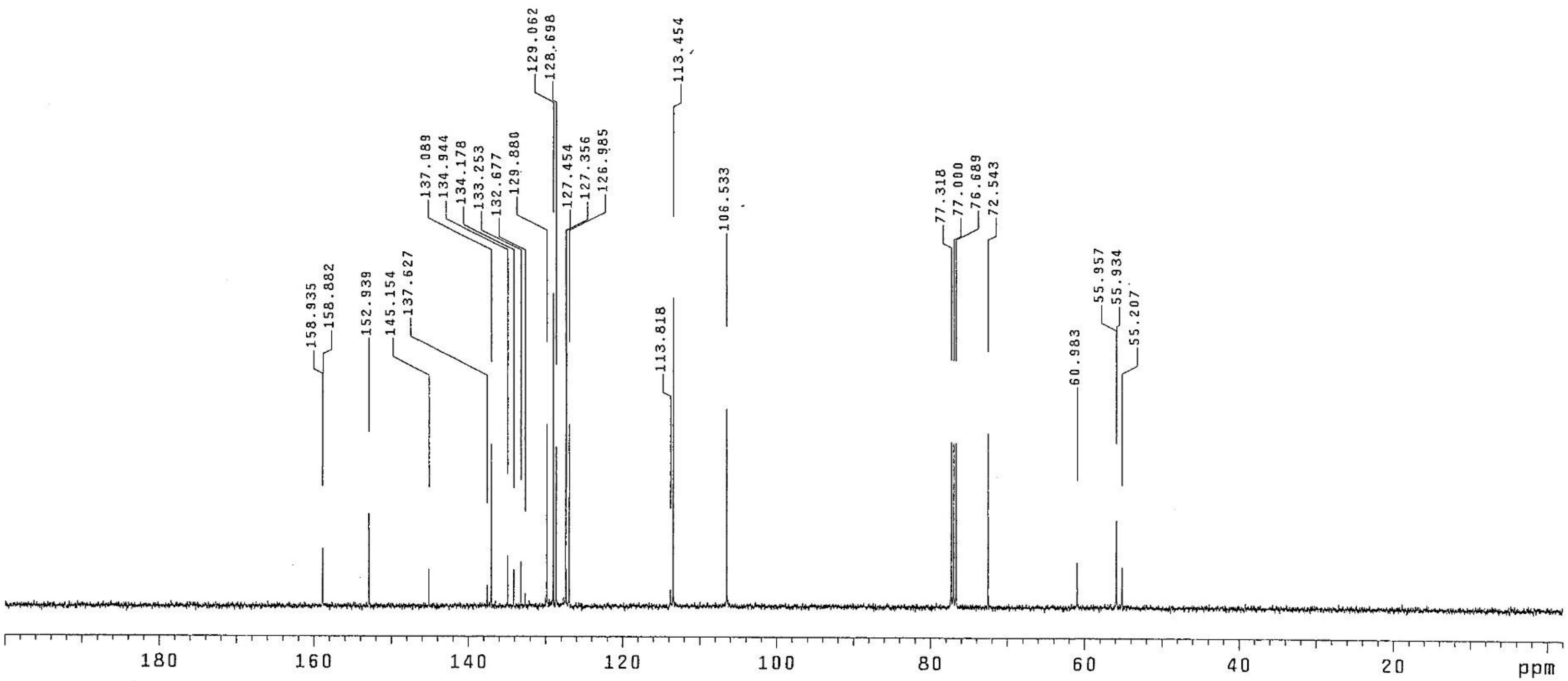

YCY-4858

Pulse Sequence: s2pul
Mercury-400BB "Mercury400"
Date: Sep 11 2012
Solvent: CDCl3
Ambient temperature
Total 32 repetitions

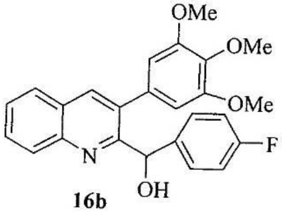

16b

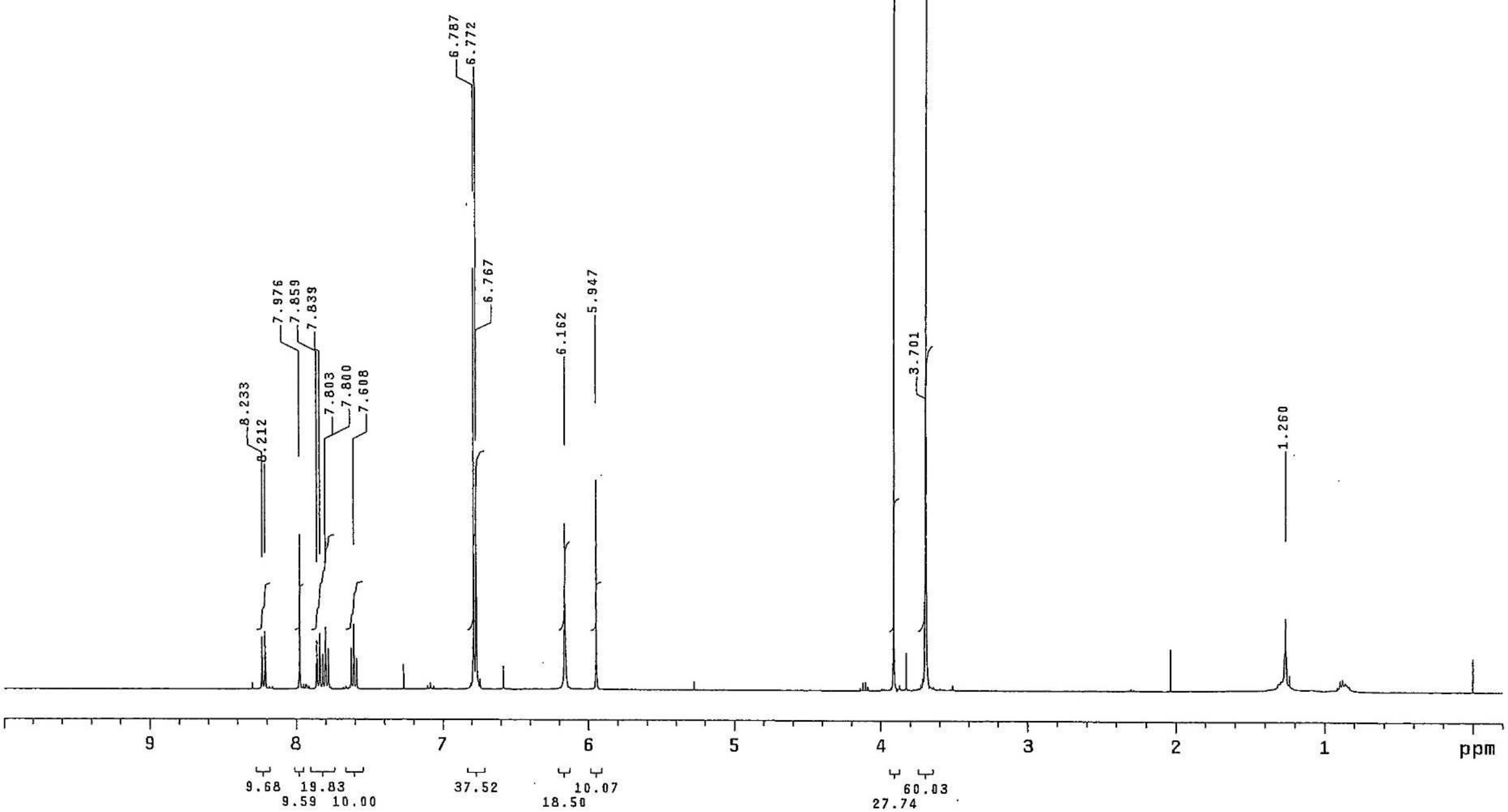

YCY-4858

Pulse Sequence: s2pul
Mercury-400BB "Mercury400"
Date: Sep 11 2012
Solvent: CDCl3
Ambient temperature
Total 608 repetitions

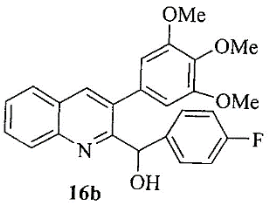

16b

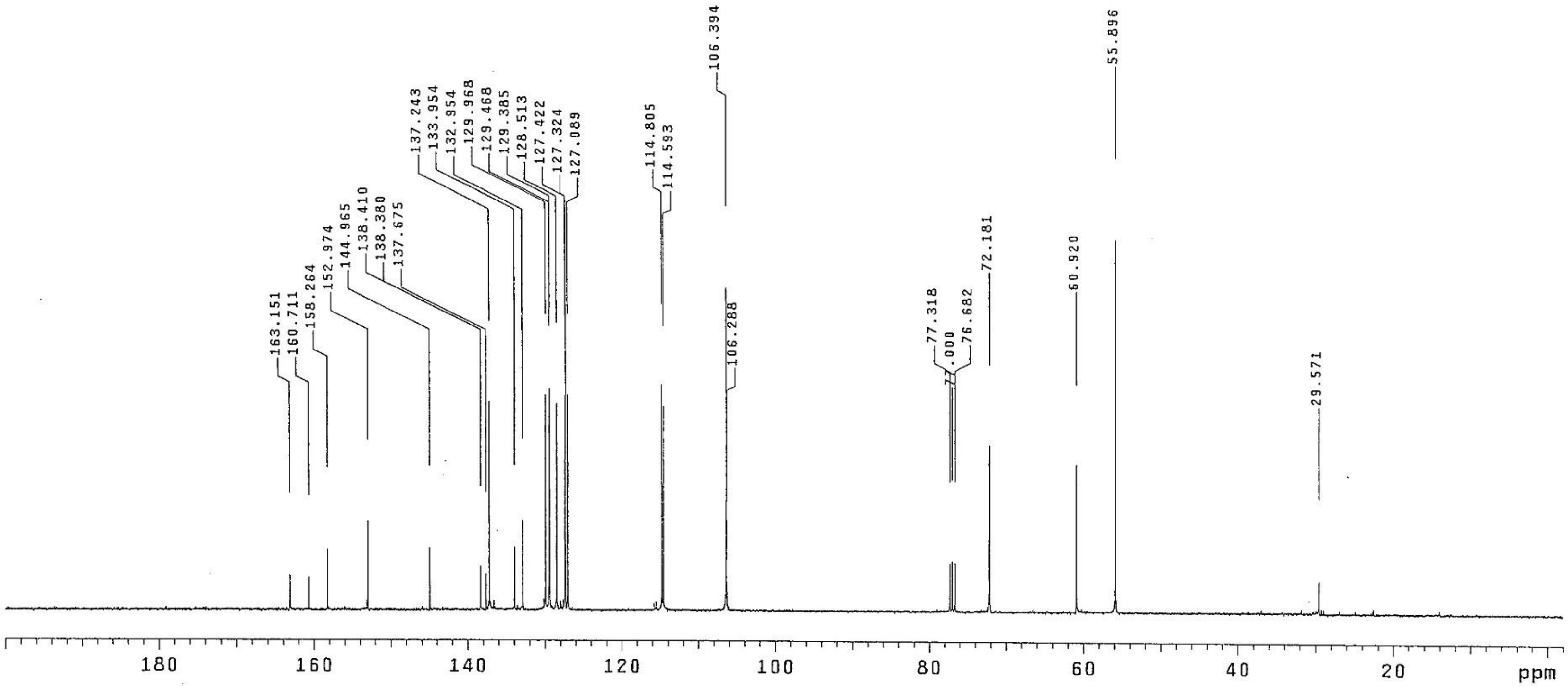

YCY-4874

Pulse Sequence: s2pul
UNITYplus-400 "unity400"
Date: Jun 14 2013
Solvent: CDCl3
Ambient temperature
Total 64 repetitions

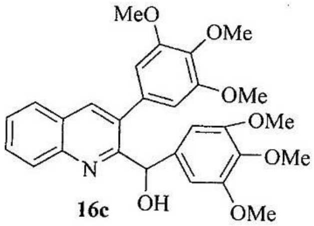

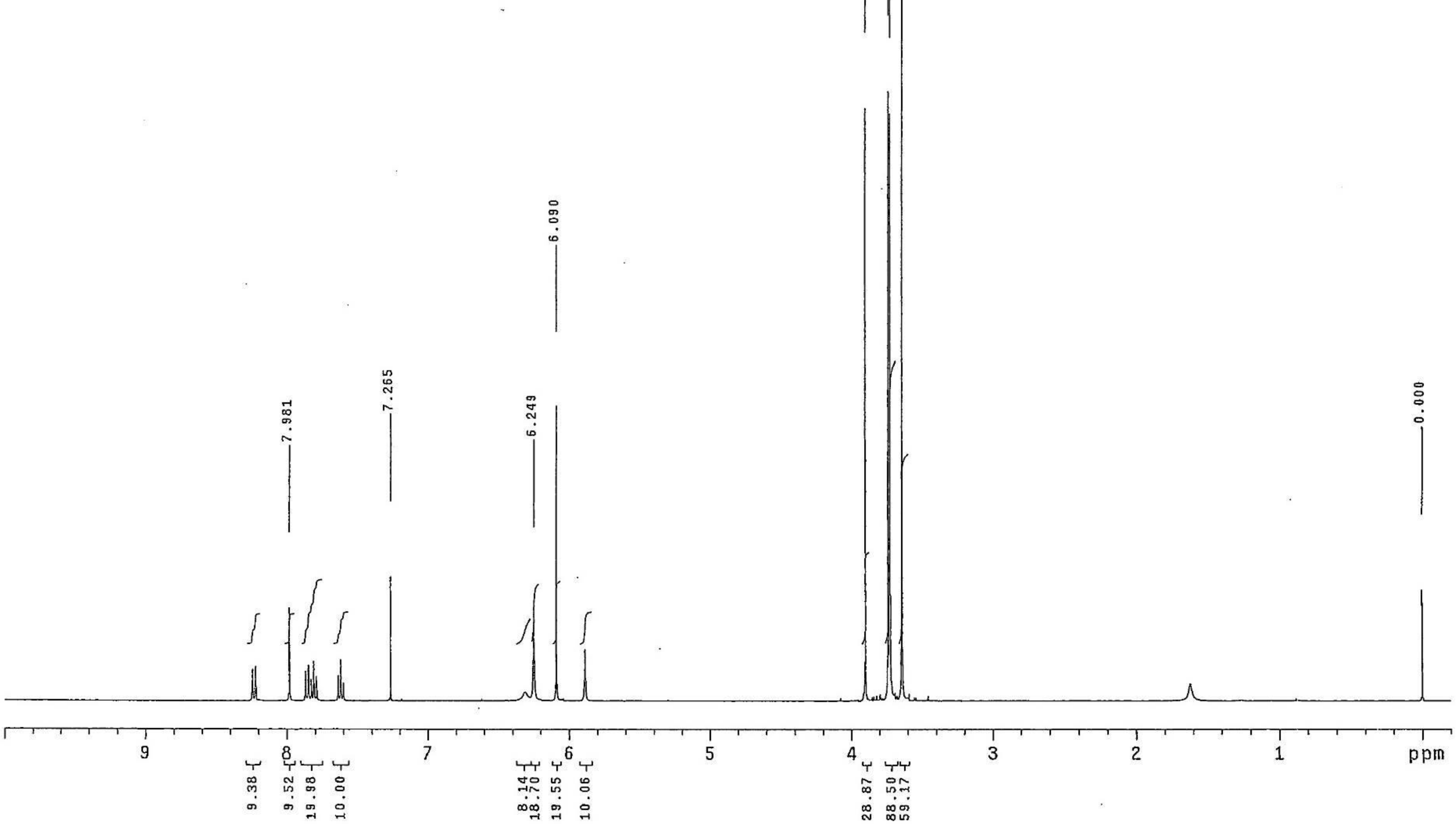

YCY-4874

Pulse Sequence: s2pul
UNITYplus-400 "unity400"
Date: Jun 14 2013
Solvent: CDCl3
Ambient temperature
Total 2048 repetitions

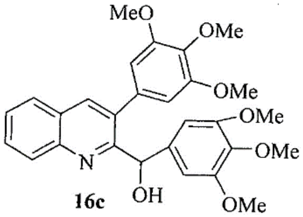

16c

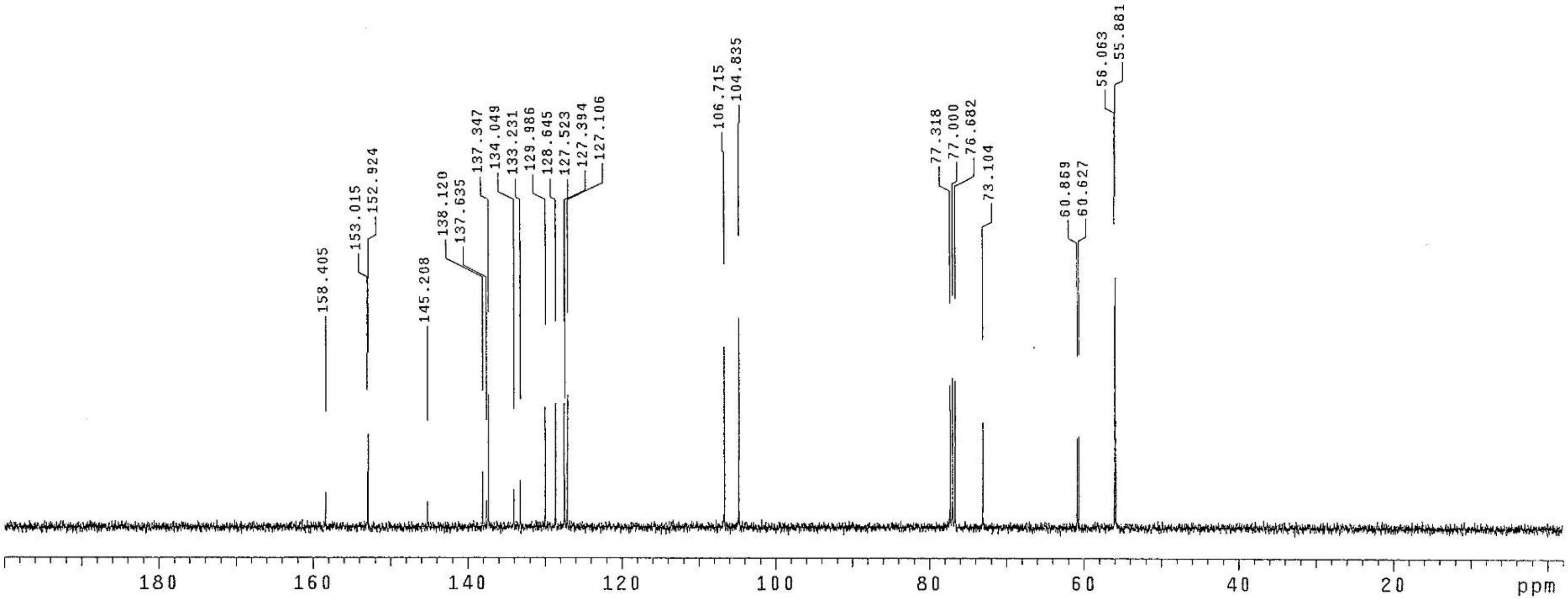

YCY-4868

Pulse Sequence: s2pul
Mercury-400BB "MercuryPlus400"
Date: Oct 29 2012
Solvent: CDCl3
Ambient temperature
Total 64 repetitions

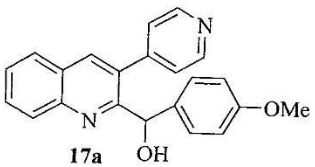

17a

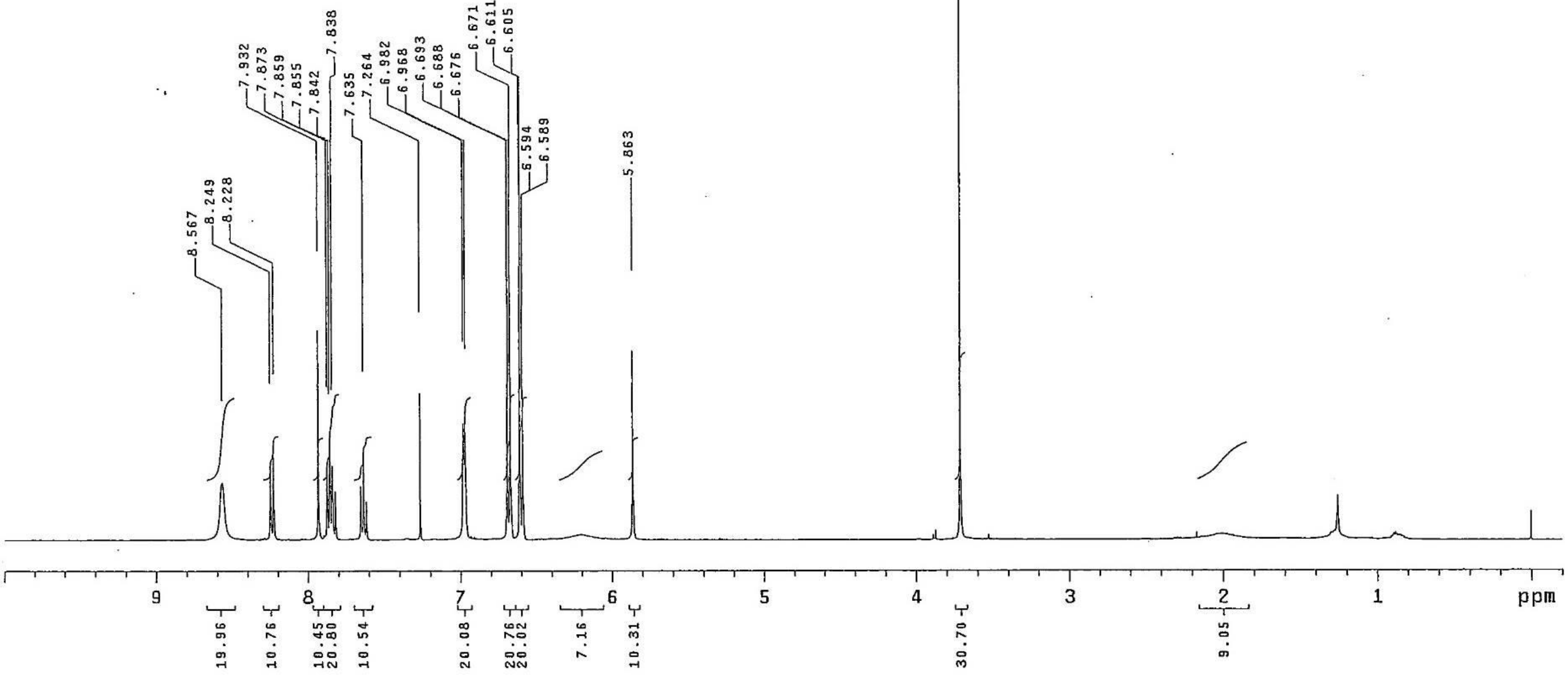

YCY-4868

Pulse Sequence: s2pul

Mercury-400BB "MercuryPlus400"
Date: Oct 29 2012
Solvent: CDCl3
Ambient temperature
Total 3808 repetitions

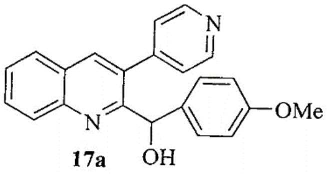

17a

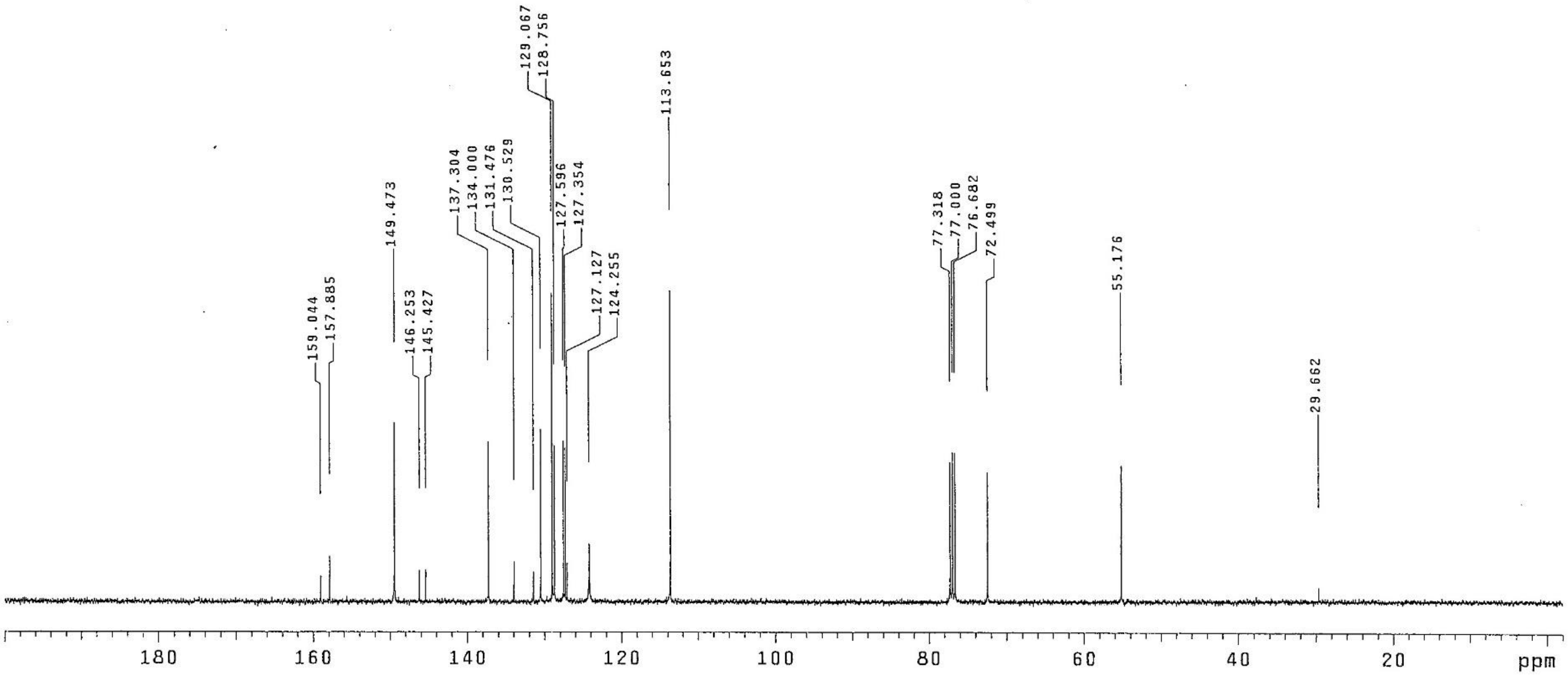

YCY-4852

Mercury-400BB "Mercuryplus400"
Date: Jun 28 2012
Solvent: CDCl3
Ambient temperature
Total 32 repetitions

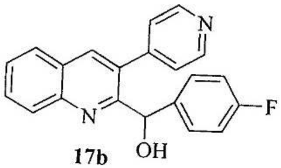

17b

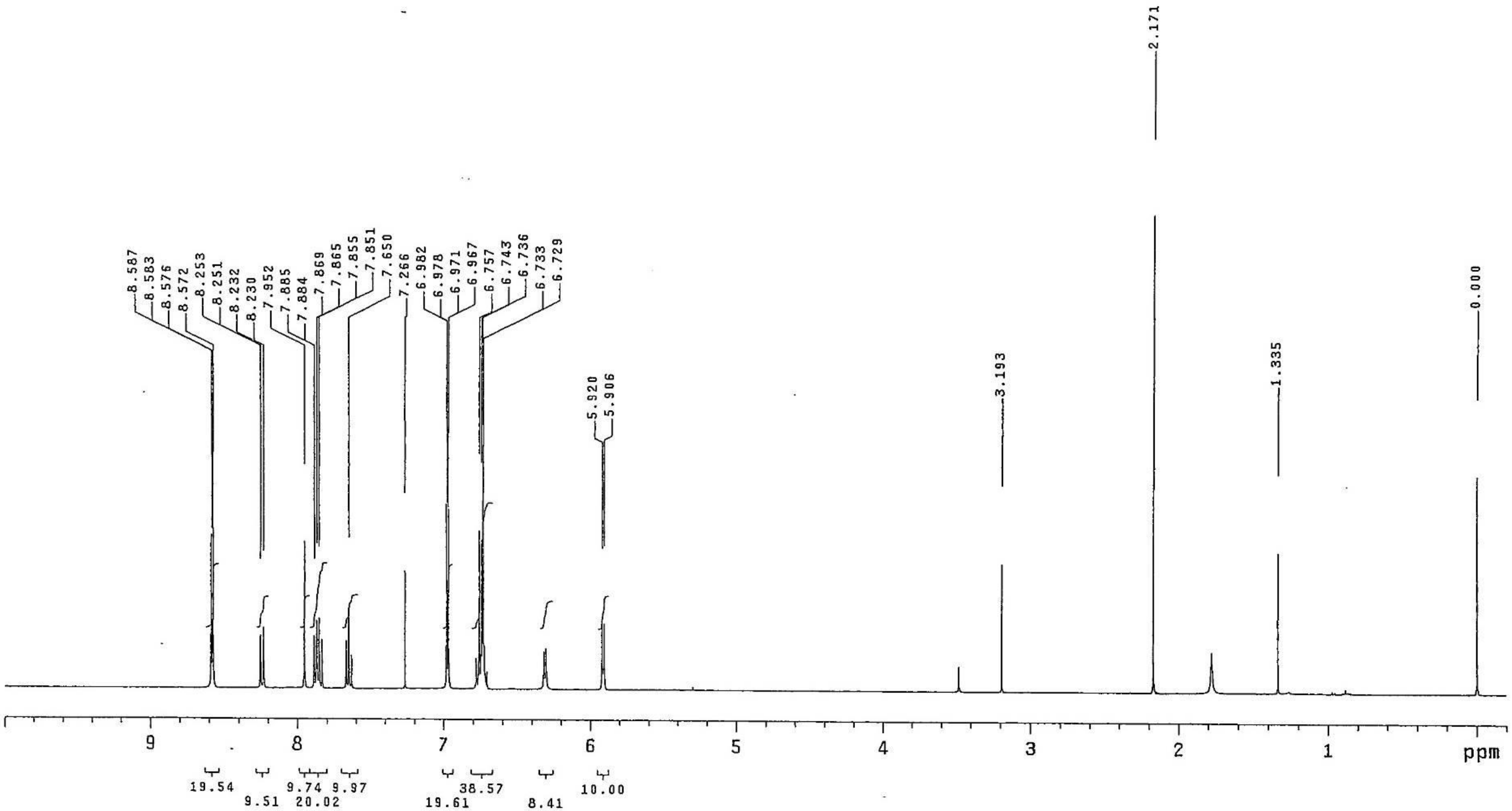

YCY-4852

Mercury-400BB "Mercuryplus400"
Date: Jun 28 2012
Solvent: CDCl3
Ambient temperature
Total 4128 repetitions

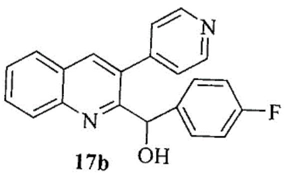

17b

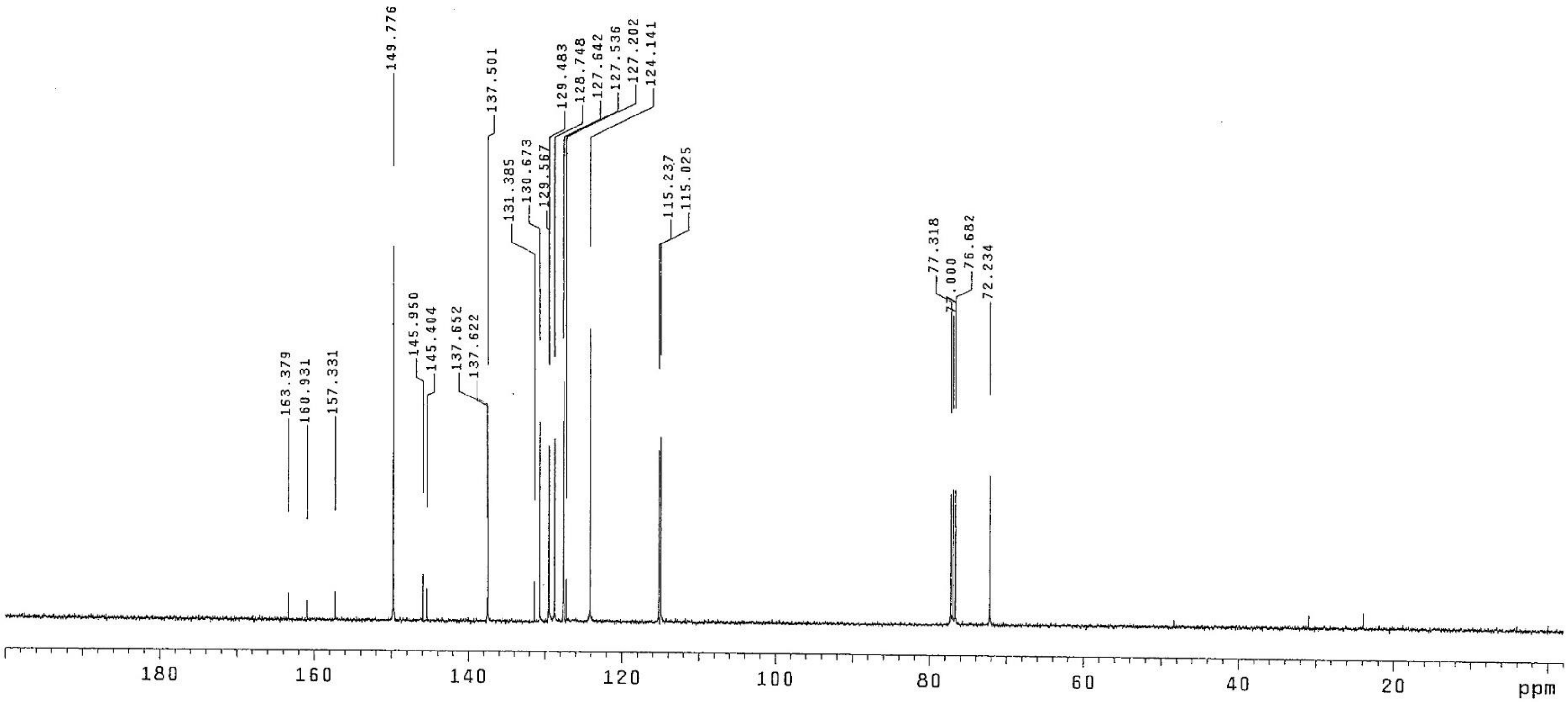

YCY-4851

Mercury-400BB "Mercuryplus400"
Date: Jun 22 2012
Solvent: CDCl3
Ambient temperature
Total 32 repetitions

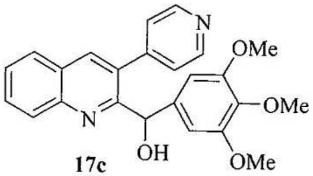

17c

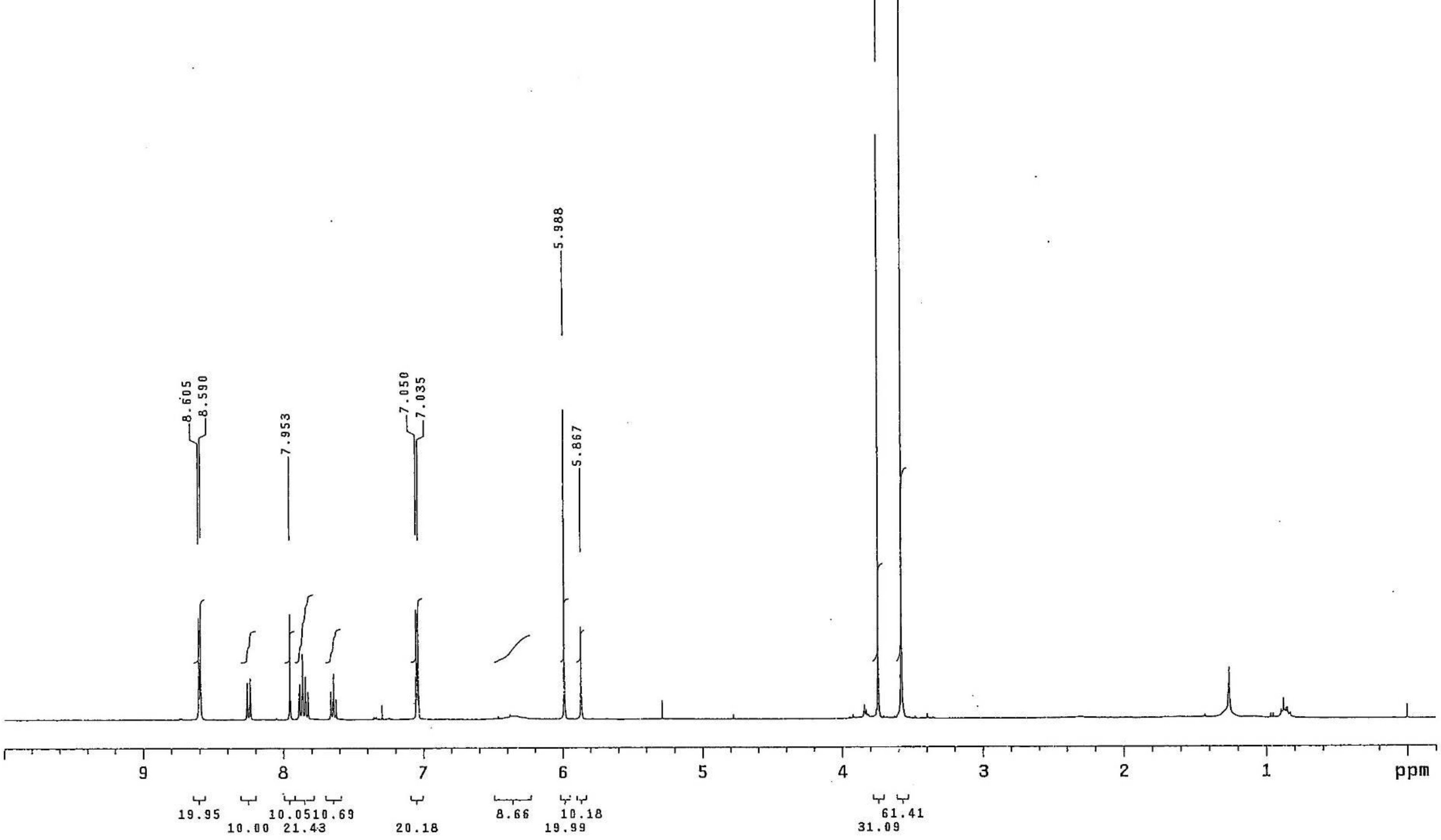

YCY-4851

Mercury-400BB "Mercuryplus400"
Date: Jun 22 2012
Solvent: CDCl3
Ambient temperature
Total 720 repetitions

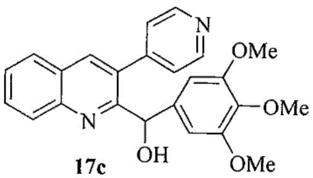

17c

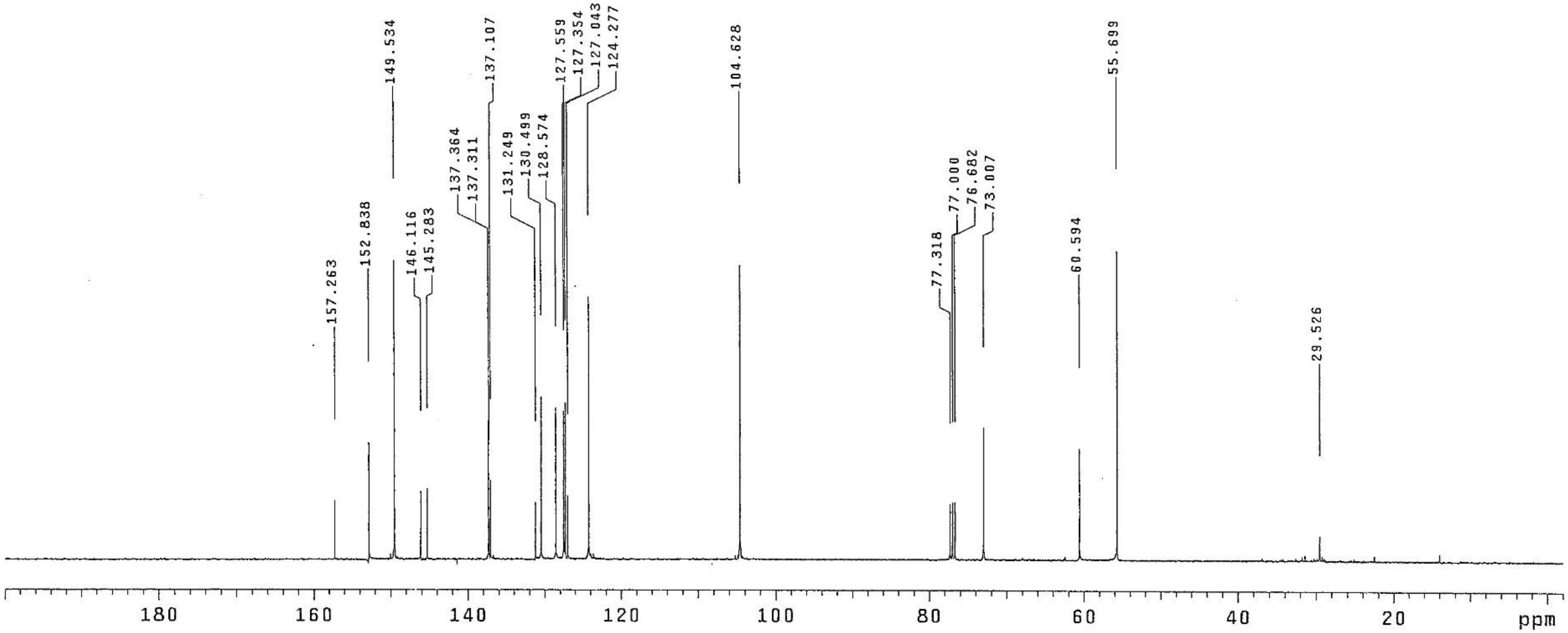

YCY-4857

Pulse Sequence: s2pul
UNITYplus-400 "unity400"
Date: Jul 30 2012
Solvent: CDCl3
Ambient temperature
Total 64 repetitions

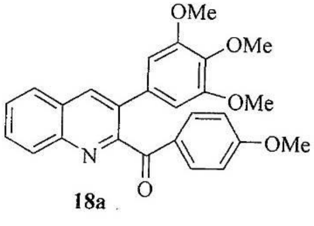

18a

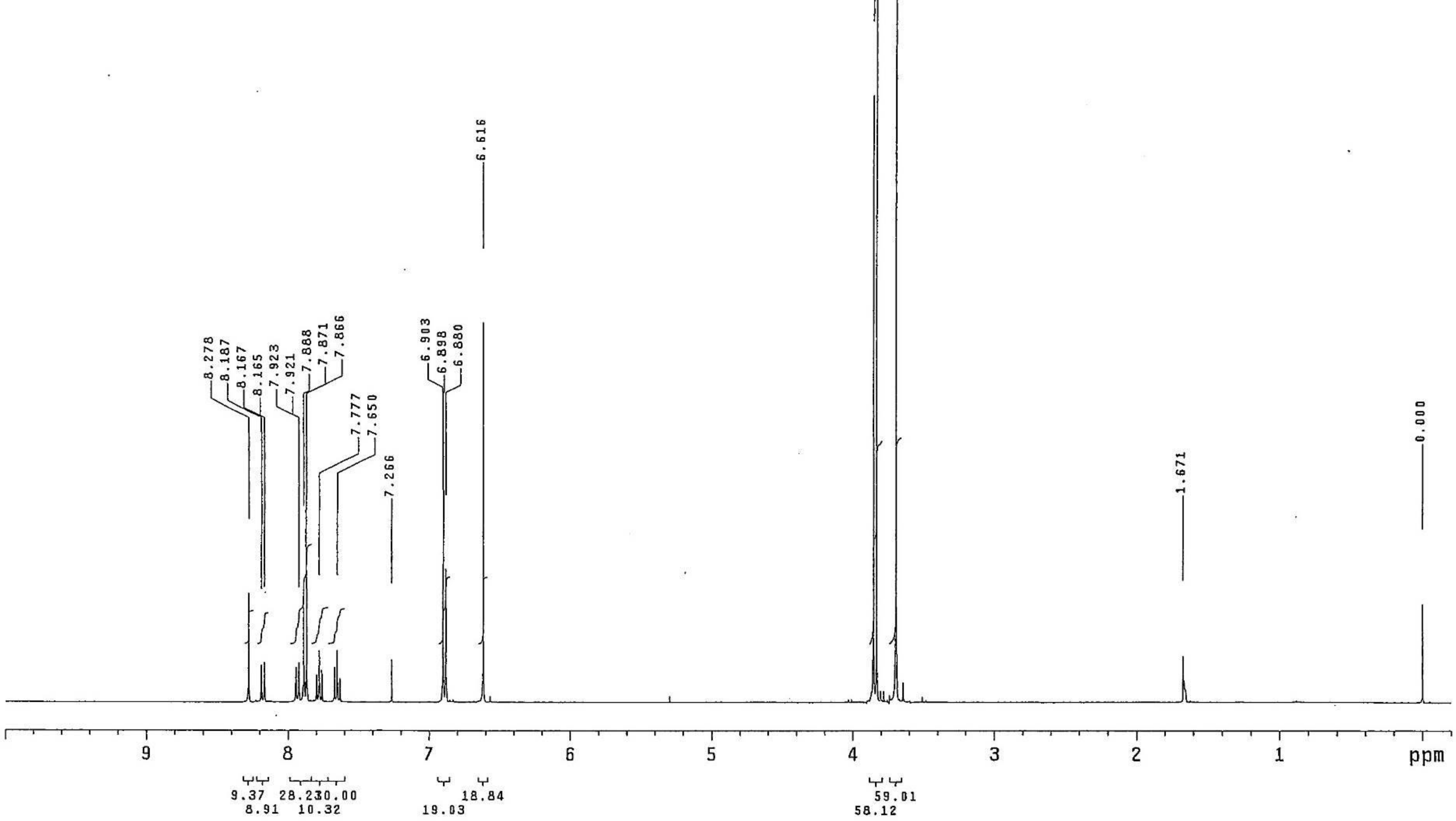

YCY-4857

Pulse Sequence: s2pul
UNITYplus-400 "unity400"
Date: Jul 30 2012
Solvent: CDCl3
Ambient temperature
Total 16000 repetitions

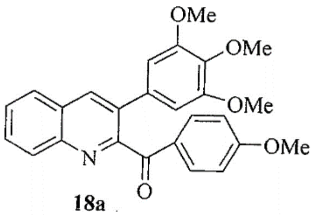

18a

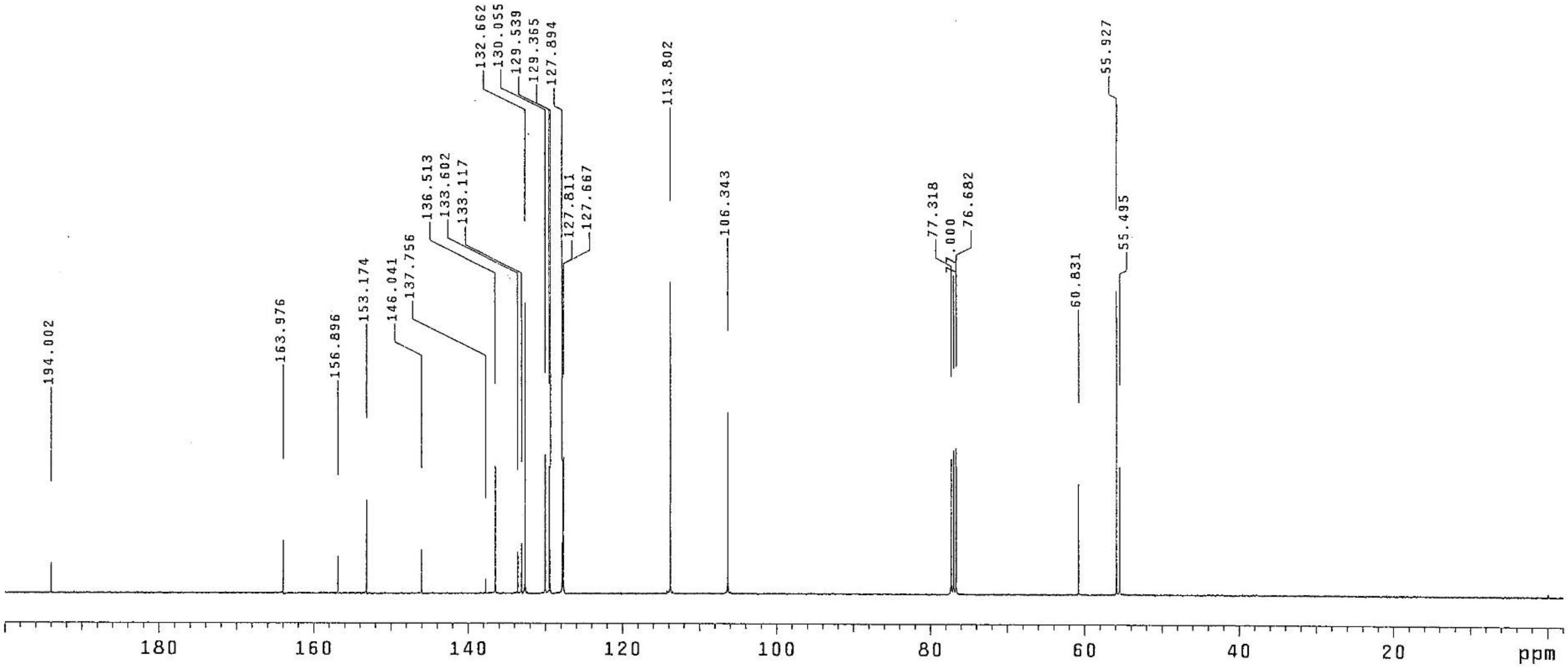

YCY-4859

Pulse Sequence: s2pul
Mercury-400BB "Mercury400"
Date: Sep 11 2012
Solvent: CDCl3
Ambient temperature
Total 72 repetitions

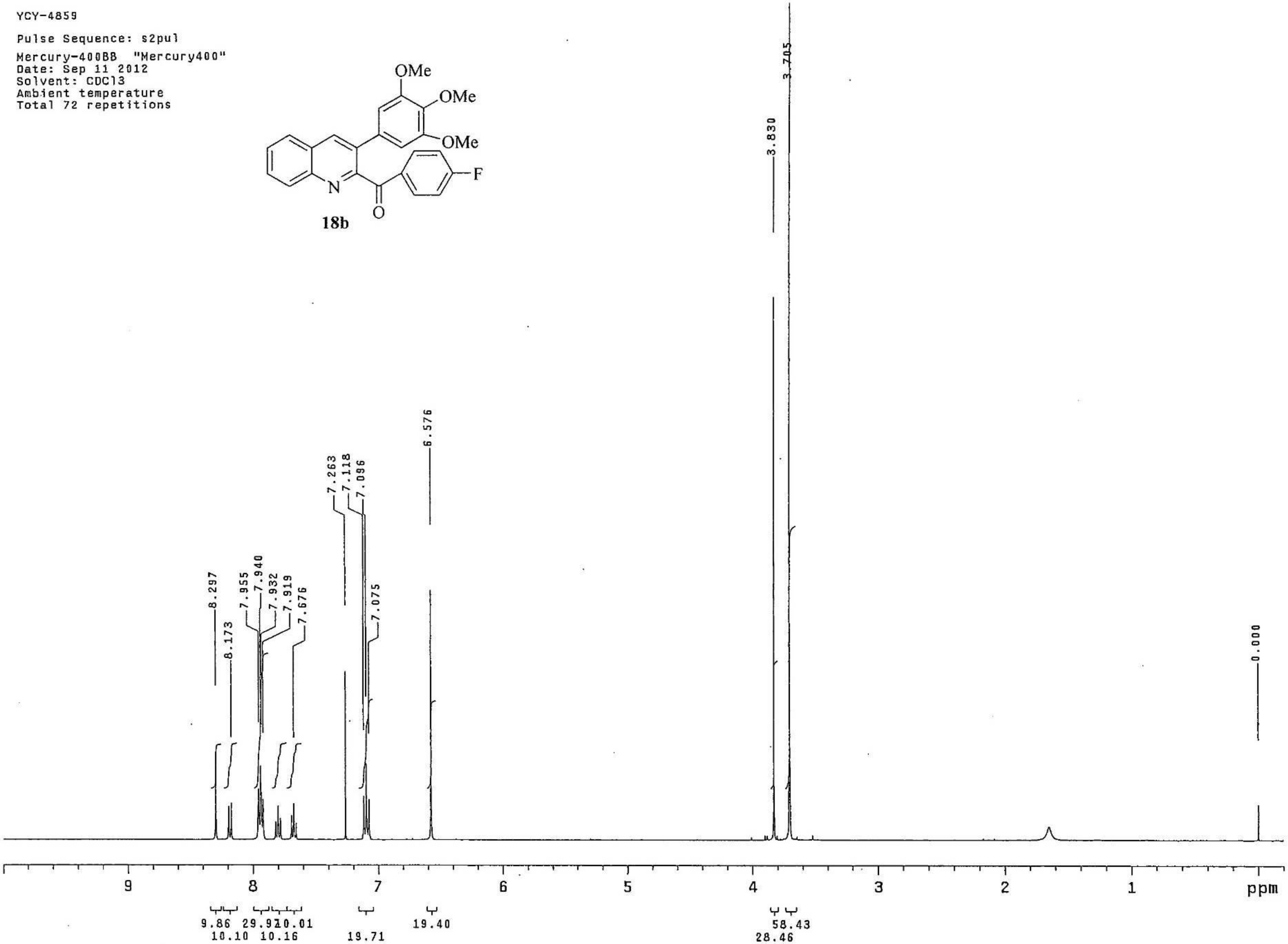

YCY-4859

Pulse Sequence: s2pul

Mercury-400BB "Mercury400"
Date: Sep 11 2012
Solvent: CDCl3
Ambient temperature
Total 4160 repetitions

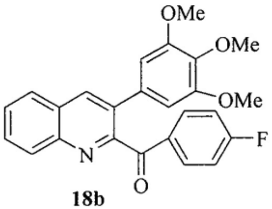

18b

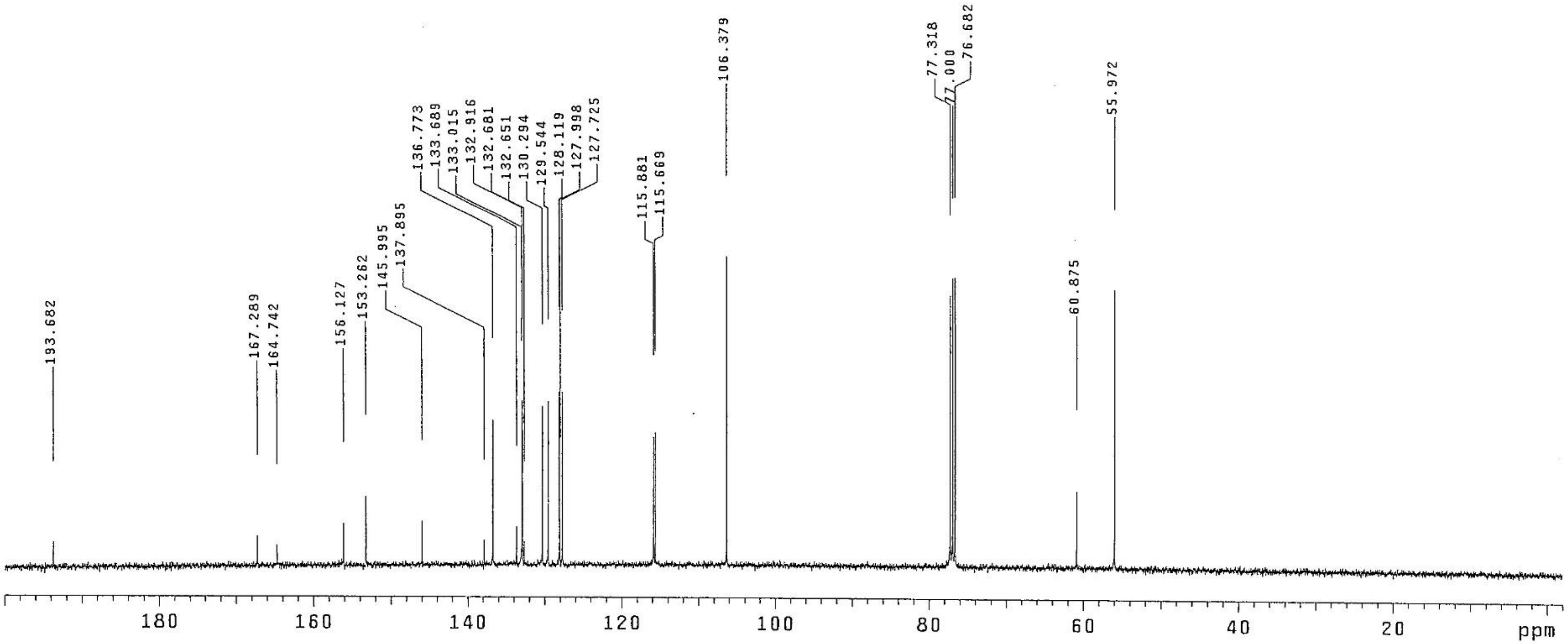

YCY-4855

Mercury-400BB "Mercuryplus400"
Date: Jul 23 2012
Solvent: CDCl3
Ambient temperature
Total 32 repetitions

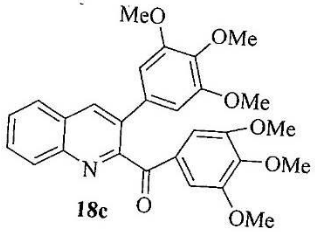

18c

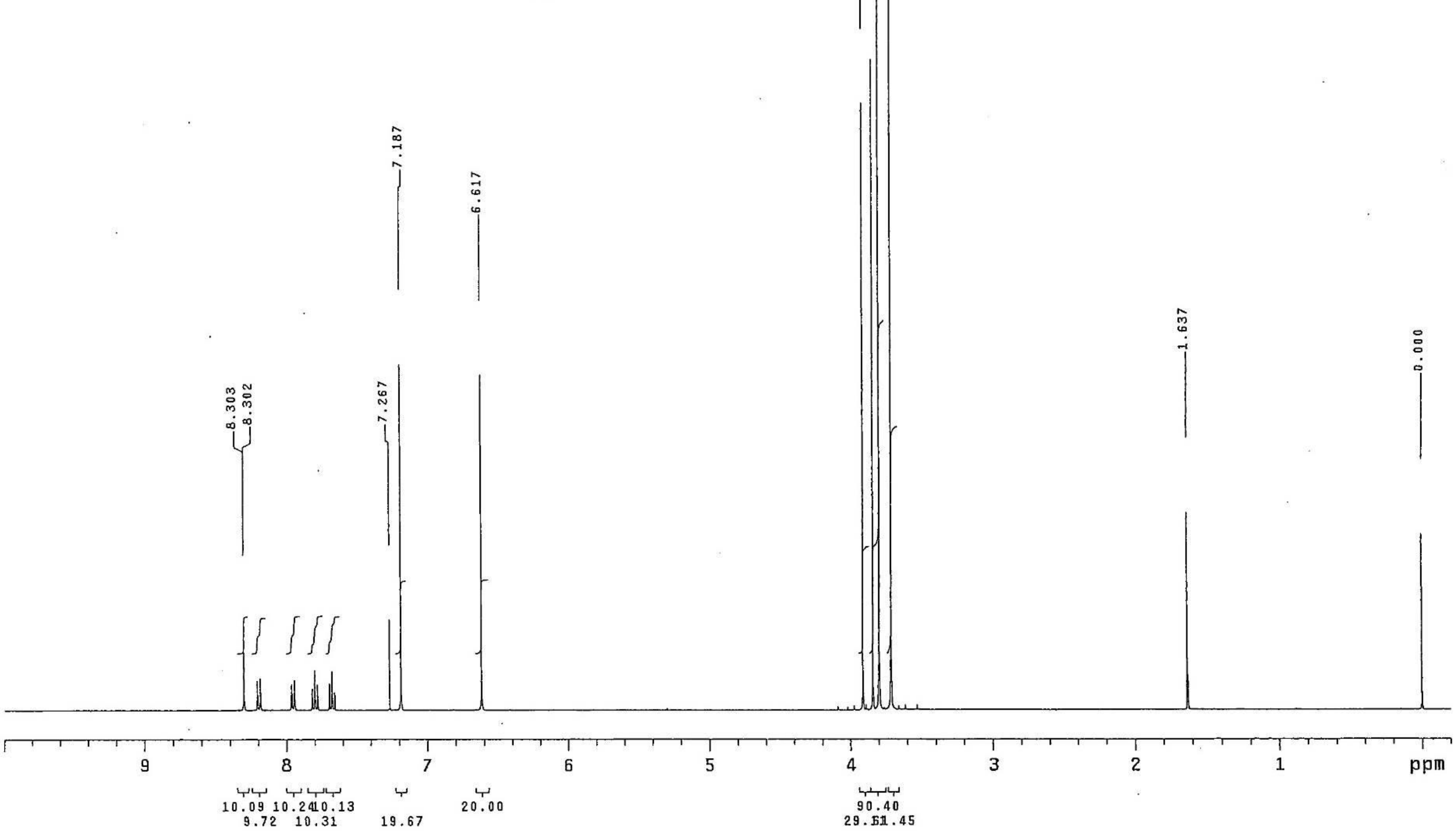

YCY-4855

Mercury-400BB "Mercuryplus400"
Date: Jul 23 2012
Solvent: CDCl3
Ambient temperature
Total 4672 repetitions

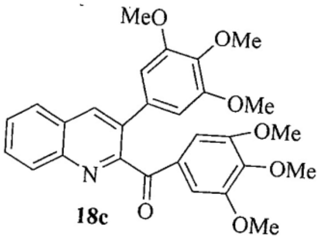

18c

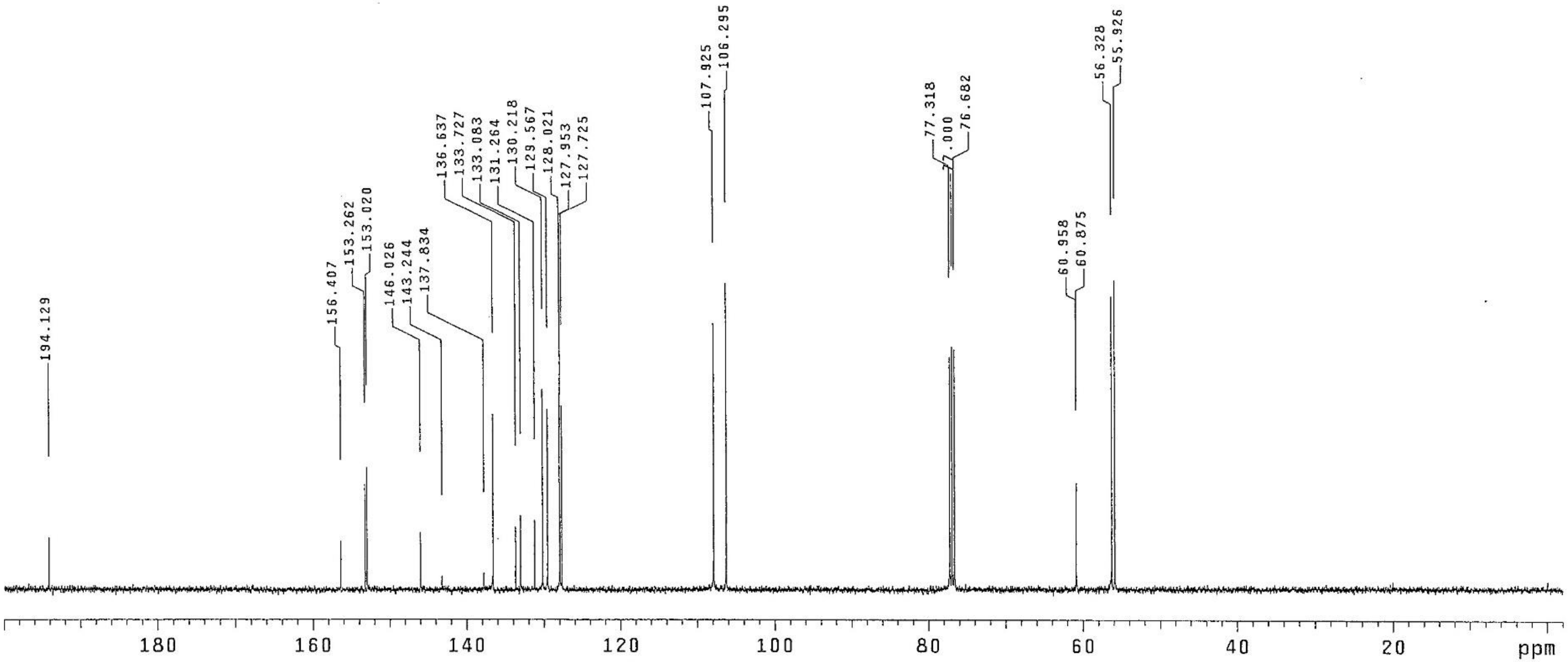

YCY-4863

Pulse Sequence: s2pul

Mercury-400BB "Mercury400"
Date: Sep 19 2012
Solvent: CDCl3
Ambient temperature
Total 64 repetitions

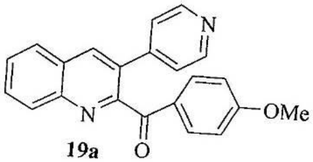

19a

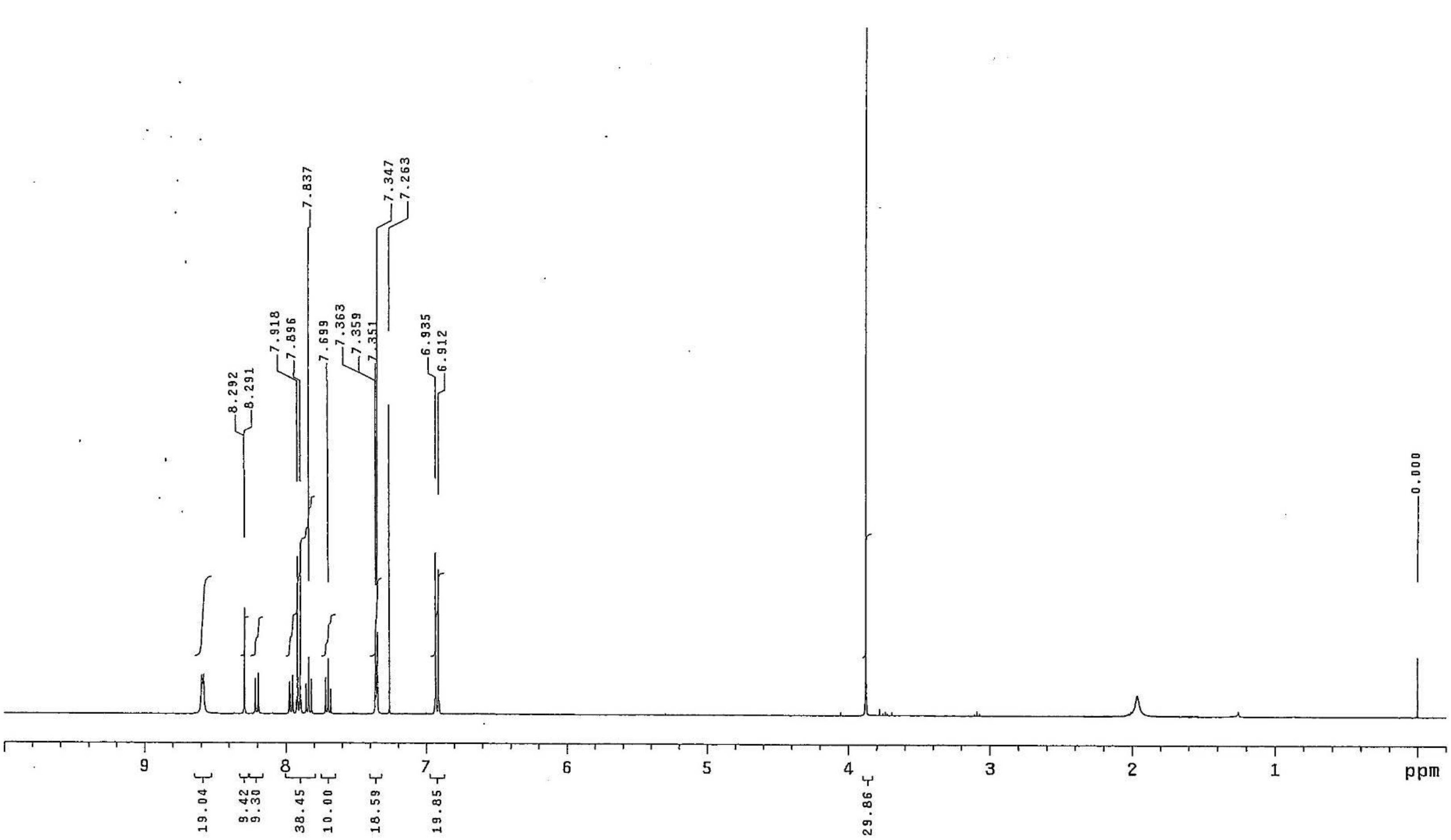

YCY-4863

Pulse Sequence: s2pul

Mercury-400BB "Mercury400"
Date: Sep 19 2012
Solvent: CDCl3
Ambient temperature
Total 6608 repetitions

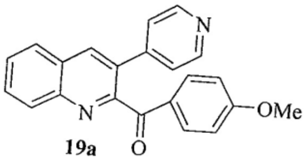

19a

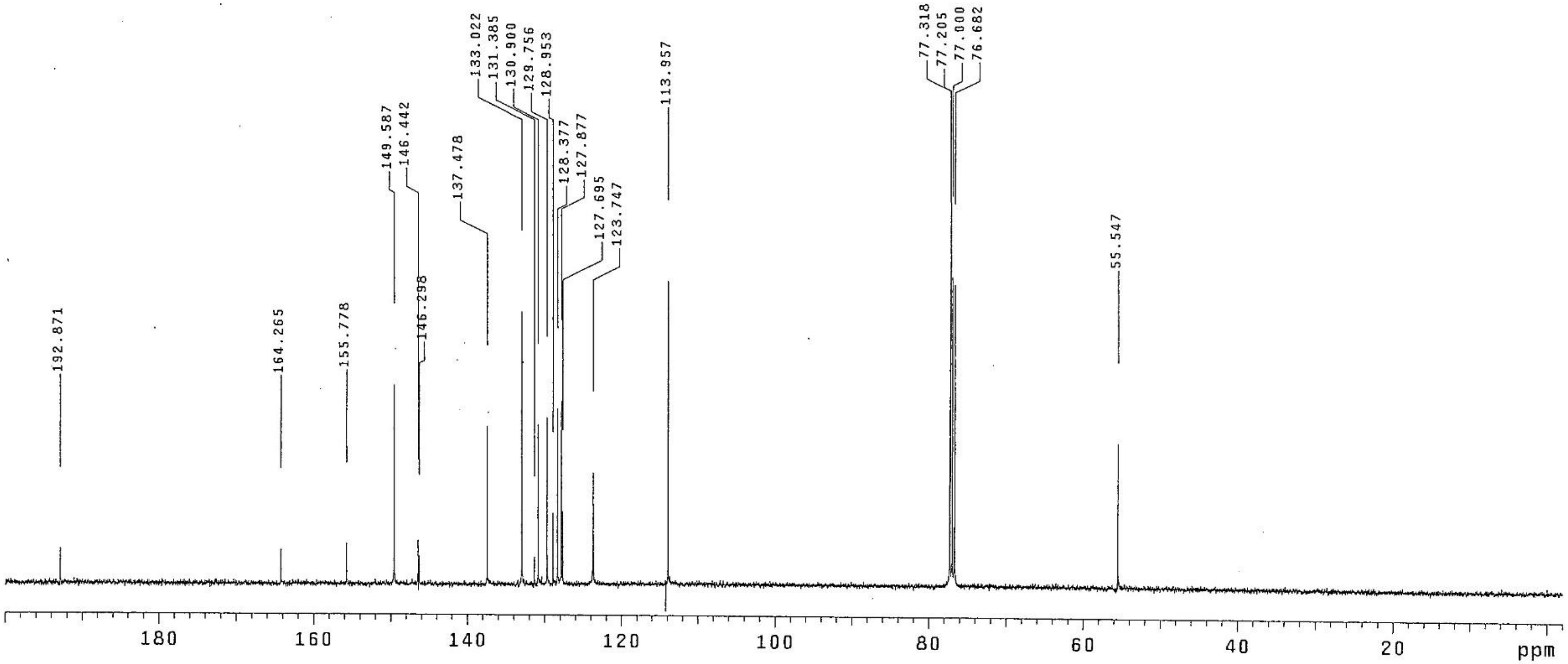

YCY-4854

Mercury-400BB "Mercuryplus400"
Date: Jul 23 2012
Solvent: CDCl3
Ambient temperature
Total 64 repetitions

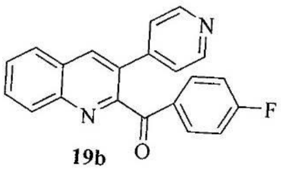

19b

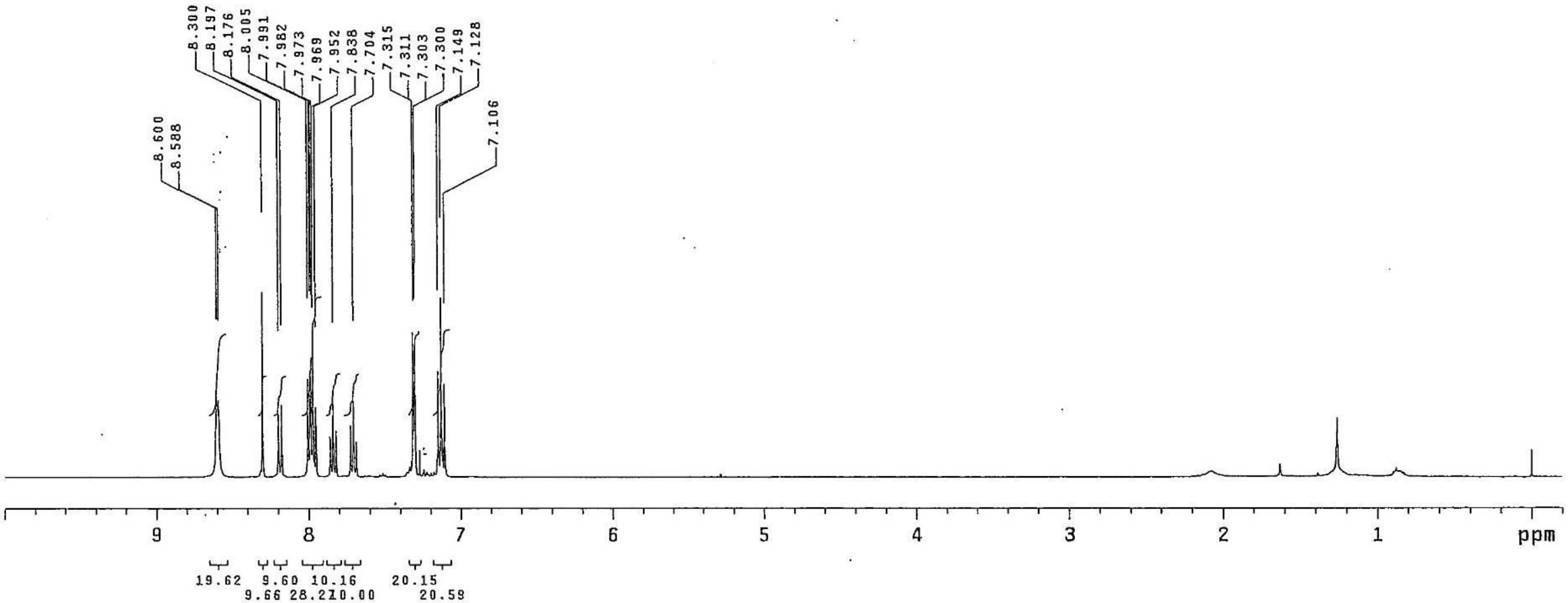

YCY-4854

Mercury-400BB "Mercuryplus400"
Date: Jul 23 2012
Solvent: CDCl3
Ambient temperature
Total 1216 repetitions

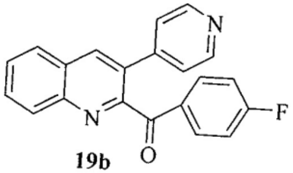

19b

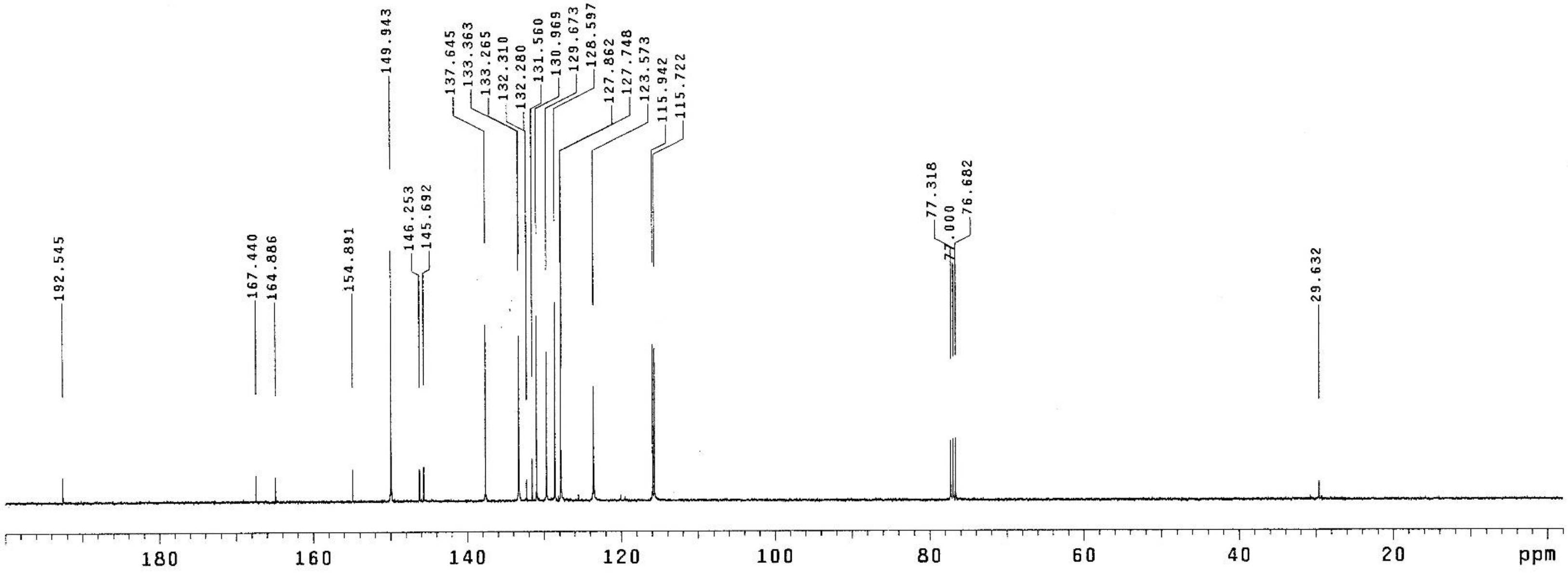

YCY-4853

Mercury-400BB "Mercuryplus400"
Date: Jul 23 2012
Solvent: CDCl3
Ambient temperature
Total 28 repetitions

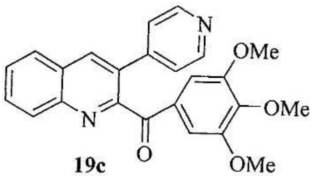

19c

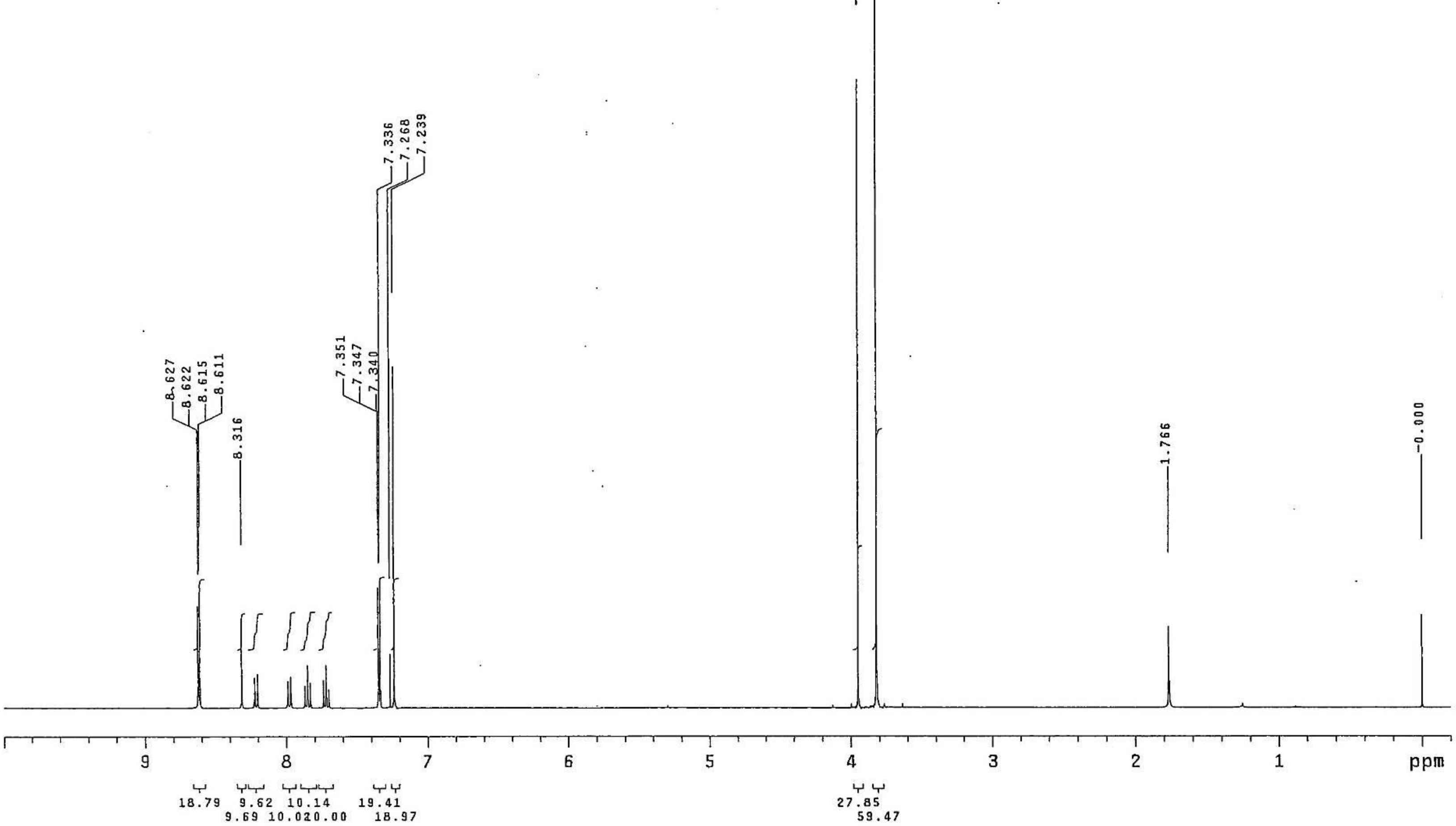

YCY-4853

Mercury-400BB "Mercuryplus400"
Date: Jul 23 2012
Solvent: CDCl3
Ambient temperature
Total 32000 repetitions

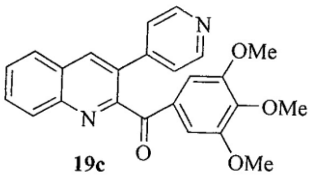

19c

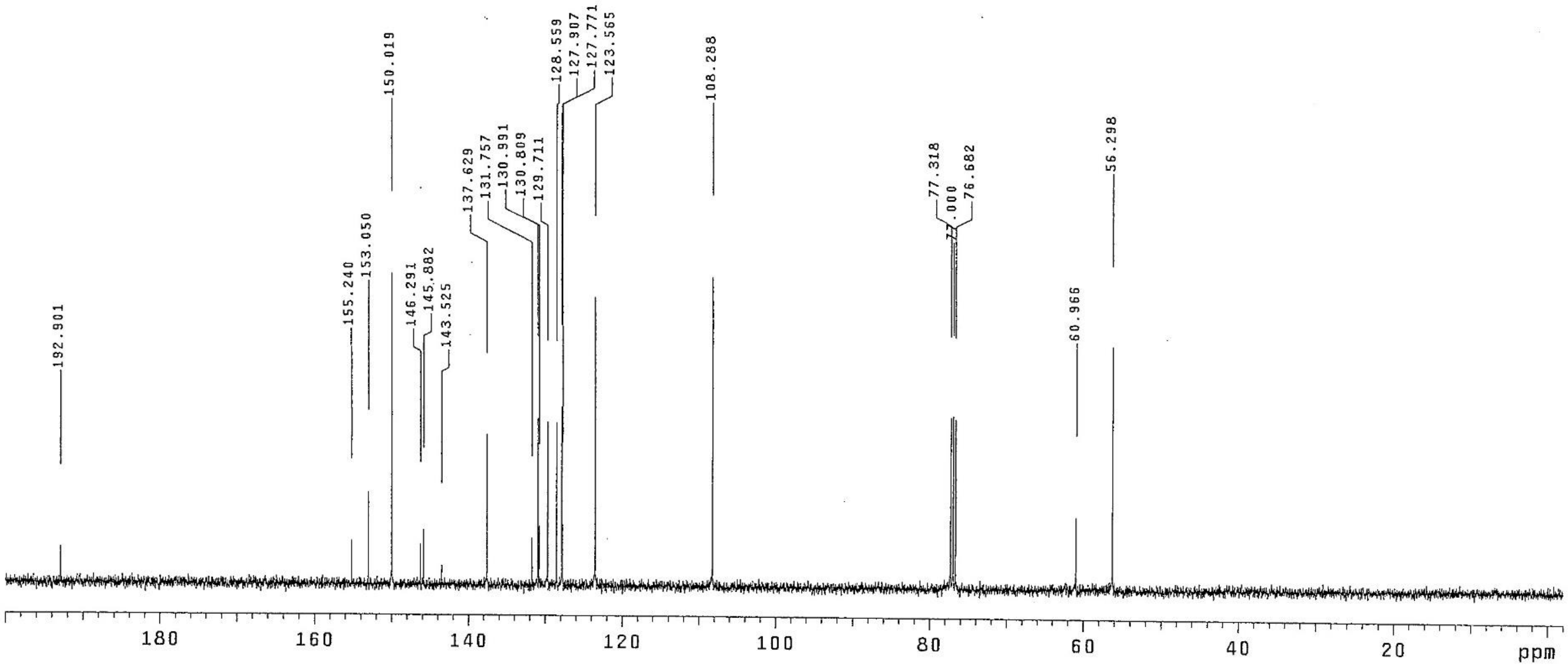

YCY-4871

Pulse Sequence: s2pul

UNITYplus-400 "unity400"
Date: Nov 22 2012
Solvent: CDCl3
Ambient temperature
Total 32 repetitions

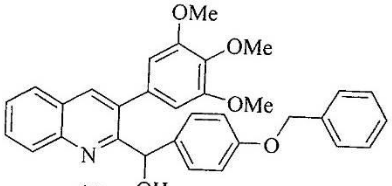

20

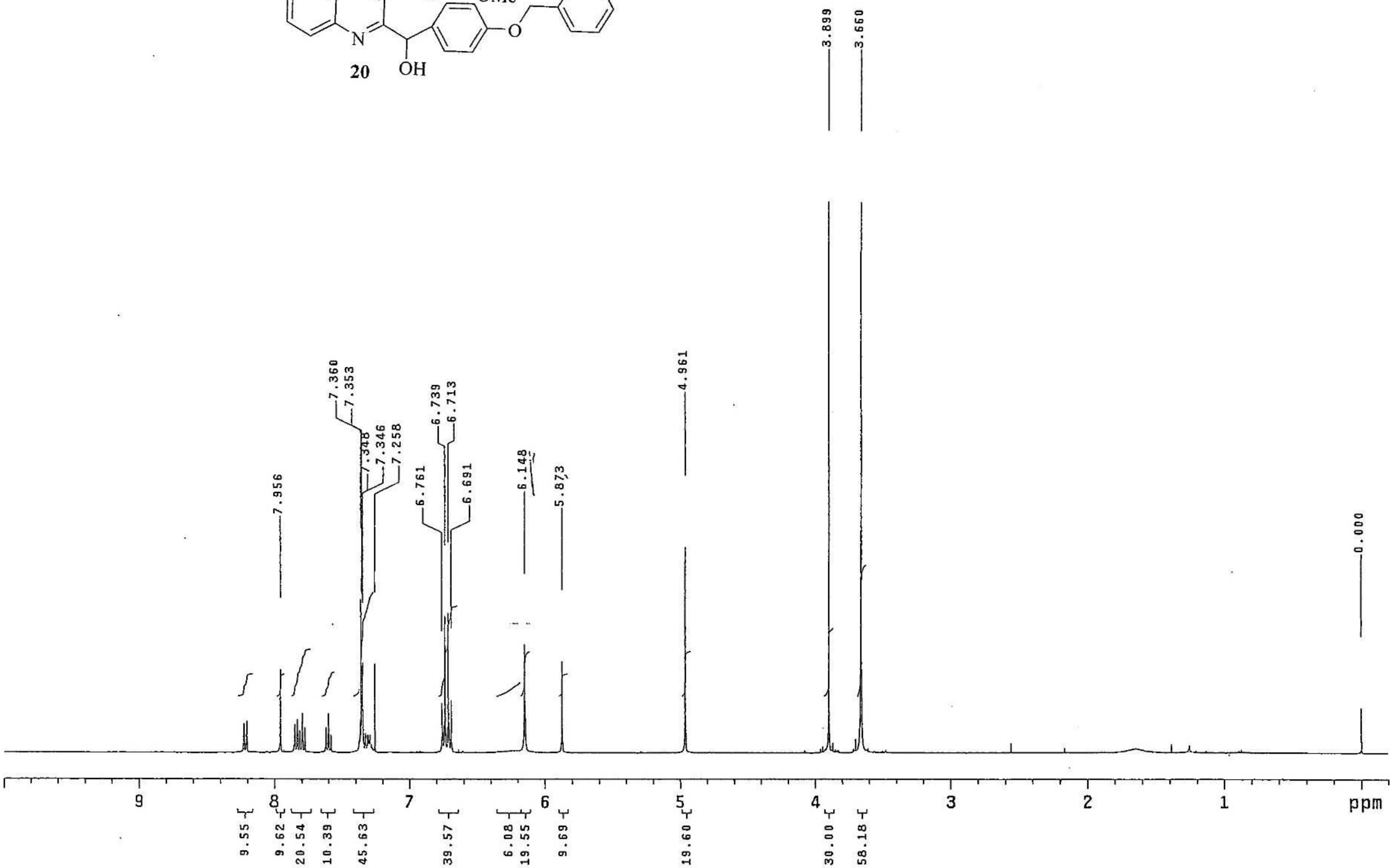

YCY-4871

Pulse Sequence: s2pul

UNITYplus-400 "unity400"
Date: Nov 22 2012
Solvent: CDCl3
Ambient temperature
Total 3168 repetitions

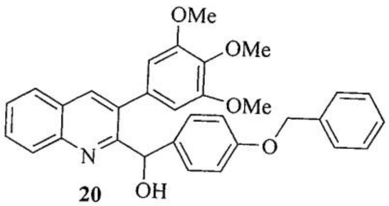

20

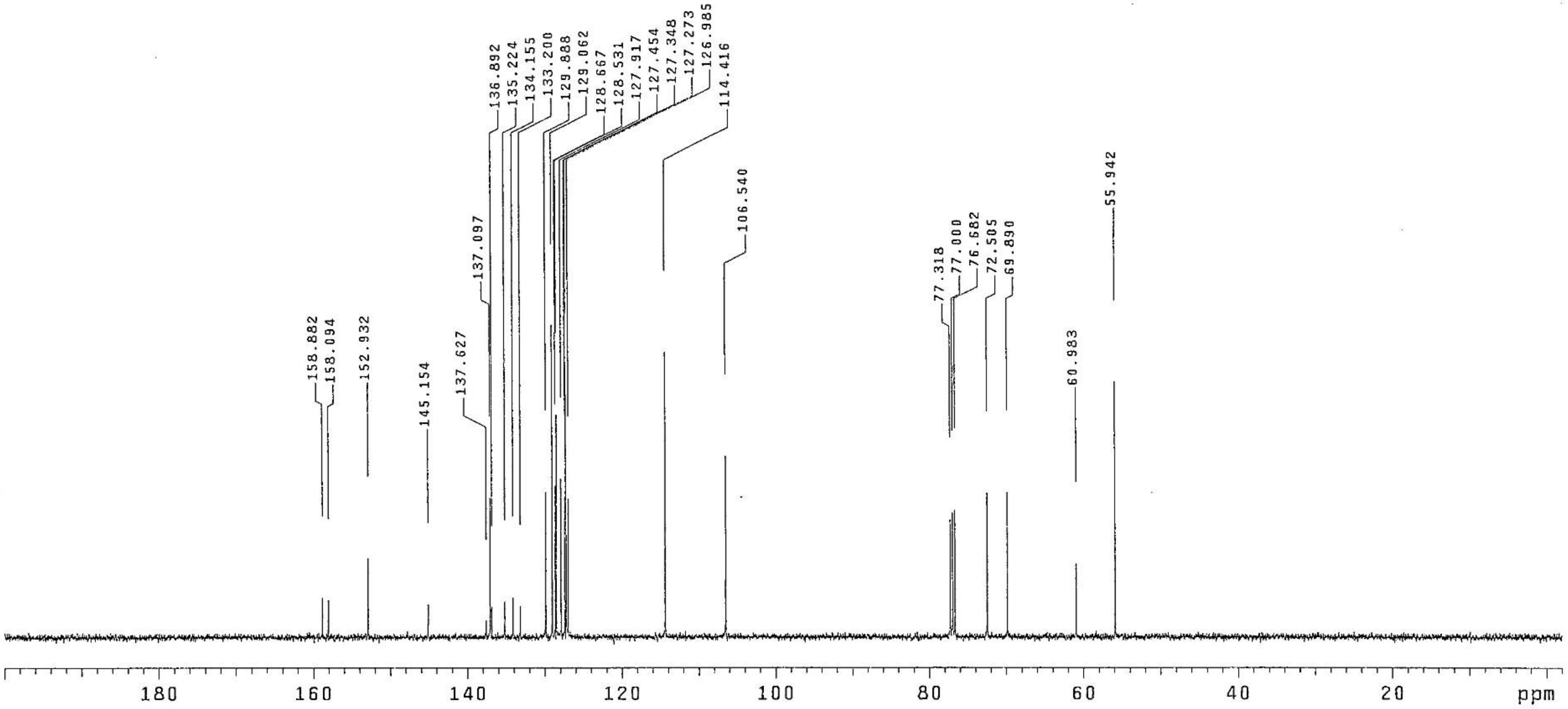

YCY-4873

Pulse Sequence: s2pul
UNITYplus-400 "unity400"
Date: Nov 22 2012
Solvent: CDCl3
Ambient temperature
Total 32 repetitions

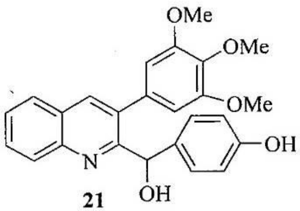

21

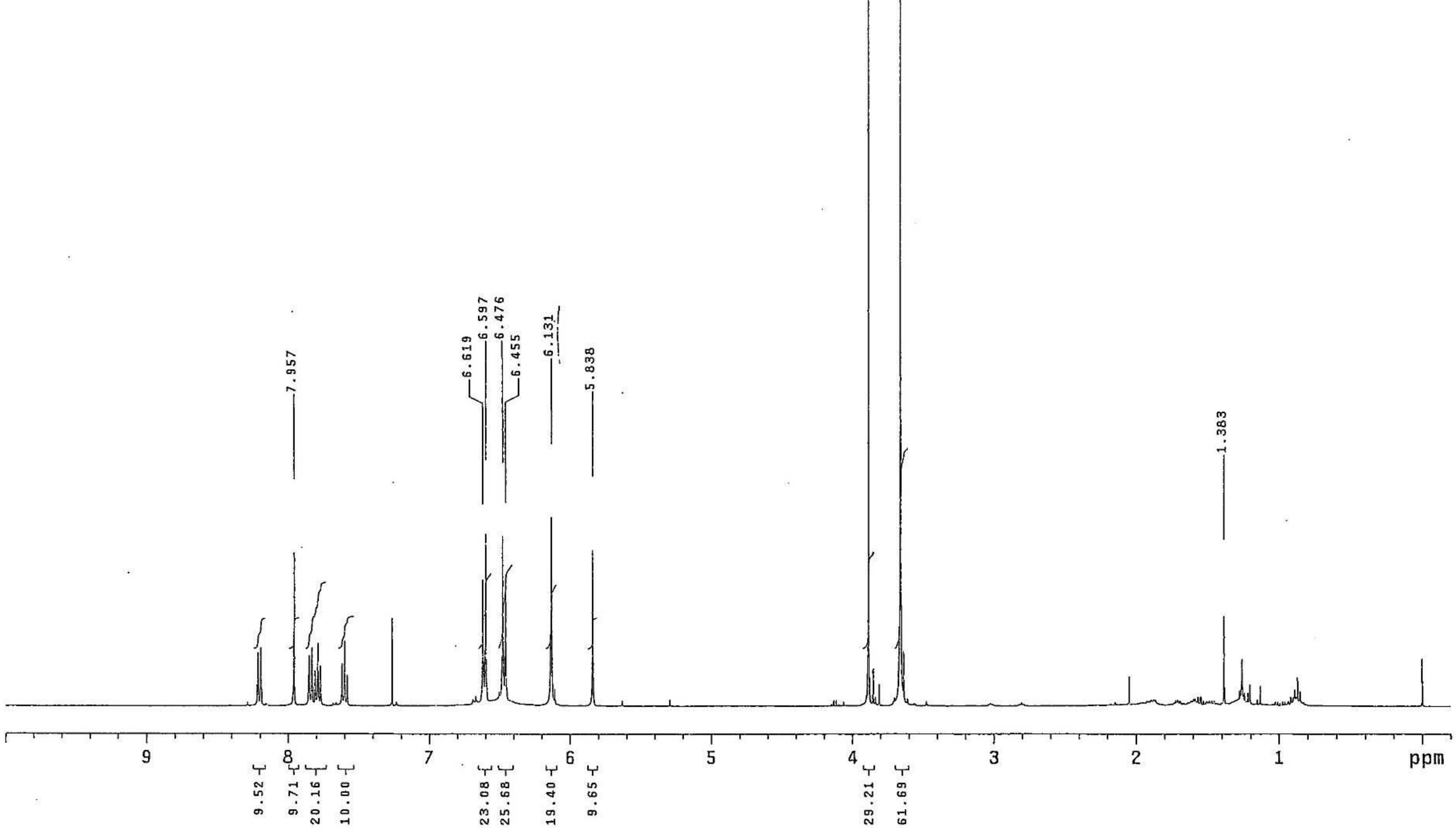

YCY-4873

Pulse Sequence: s2pul
UNITYplus-400 "unity400"
Date: Nov 22 2012
Solvent: CDCl3
Ambient temperature
Total 2192 repetitions

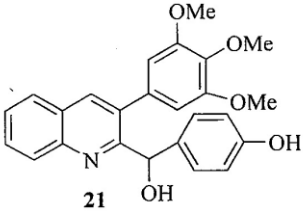

21

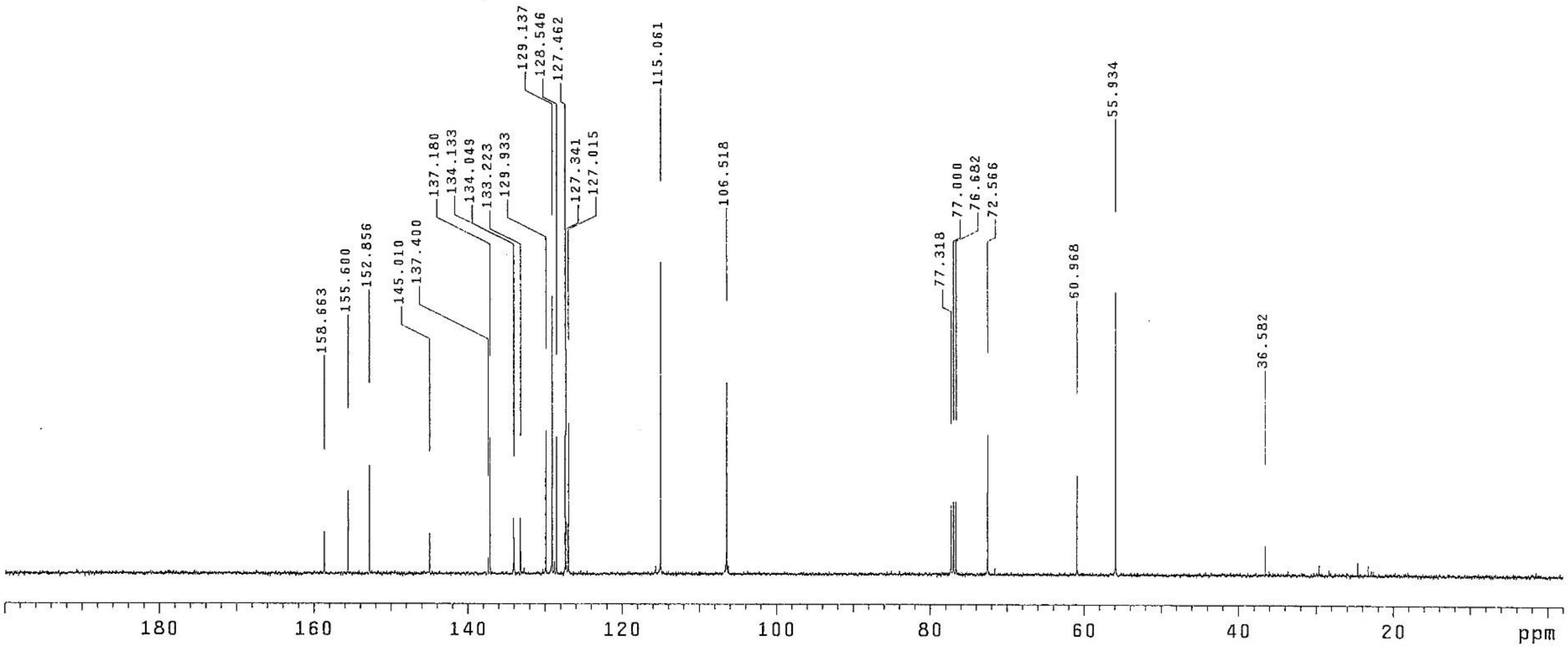

YCY-4870

Pulse Sequence: s2pul

Solvent: DMSO
Ambient temperature
Mercury-400BB "MercuryPlus400"

Pulse 48.1 degrees
Acq. time 4.002 sec
Width 5995.2 Hz
80 repetitions
OBSERVE H1, 400.3997885 MHz
DATA PROCESSING
FT size 65536
Total time 6 min, 23 sec

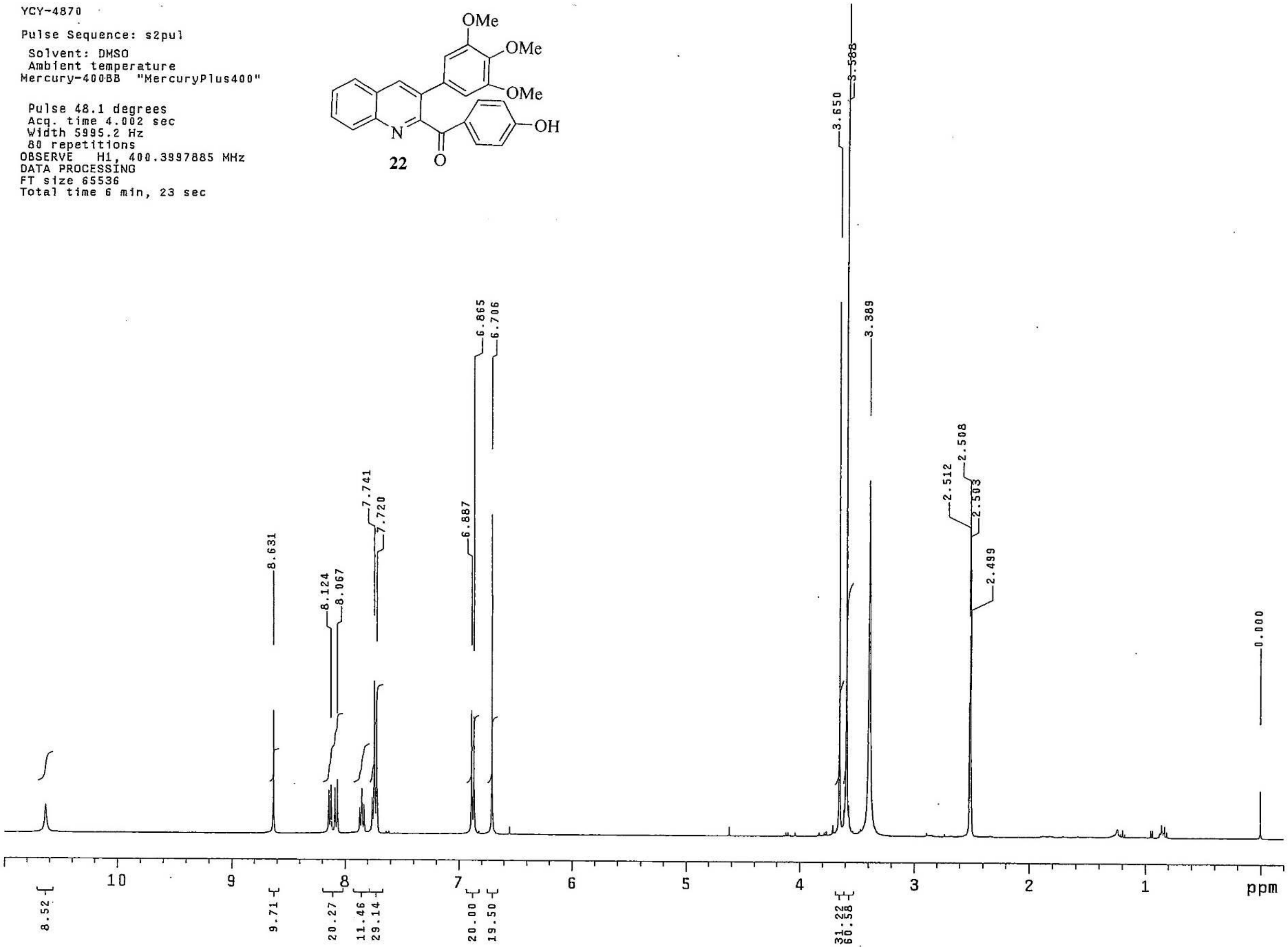

YCY-4870

Pulse Sequence: s2pul

Solvent: DMSO
Ambient temperature
Mercury-400BB "MercuryPlus400"

Pulse 68.7 degrees
Acq. time 1.000 sec
Width 25000.0 Hz
4528 repetitions
OBSERVE C13, 100.6806537 MHz
DECOUPLE H1, 400.4018591 MHz
Power 38 dB
continuously on
WALTZ-16 modulated
DATA PROCESSING
Line broadening 1.0 Hz
FT size 65536
Total time 27 hr, 19 min, 27 sec

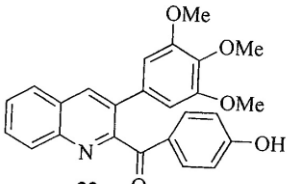

22

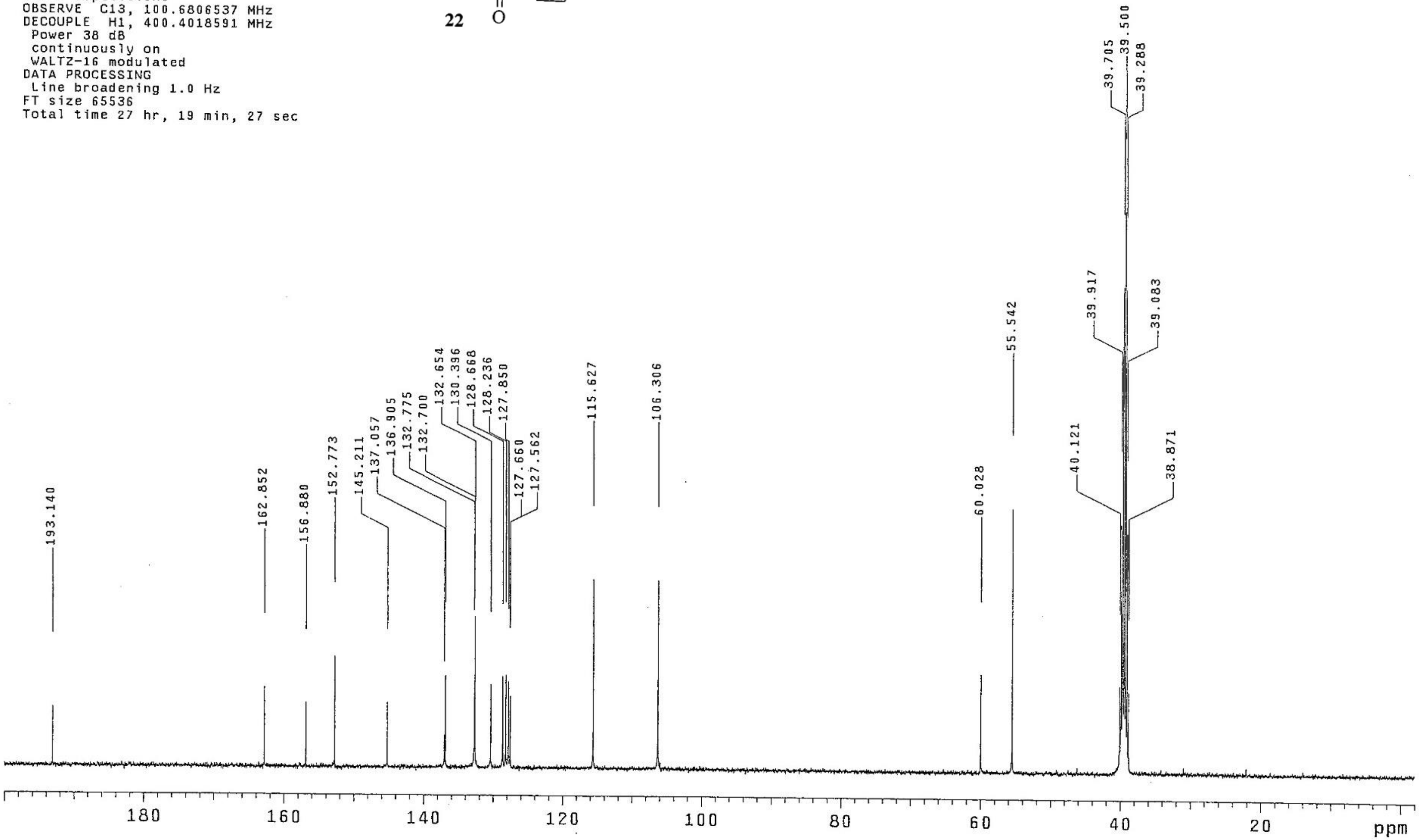

YCY-4864

Pulse Sequence: s2pul
UNITYplus-400 "unity400"
Date: Oct 3 2012
Solvent: DMSO
Ambient temperature
Total 64 repetitions

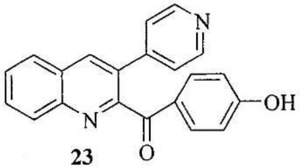

23

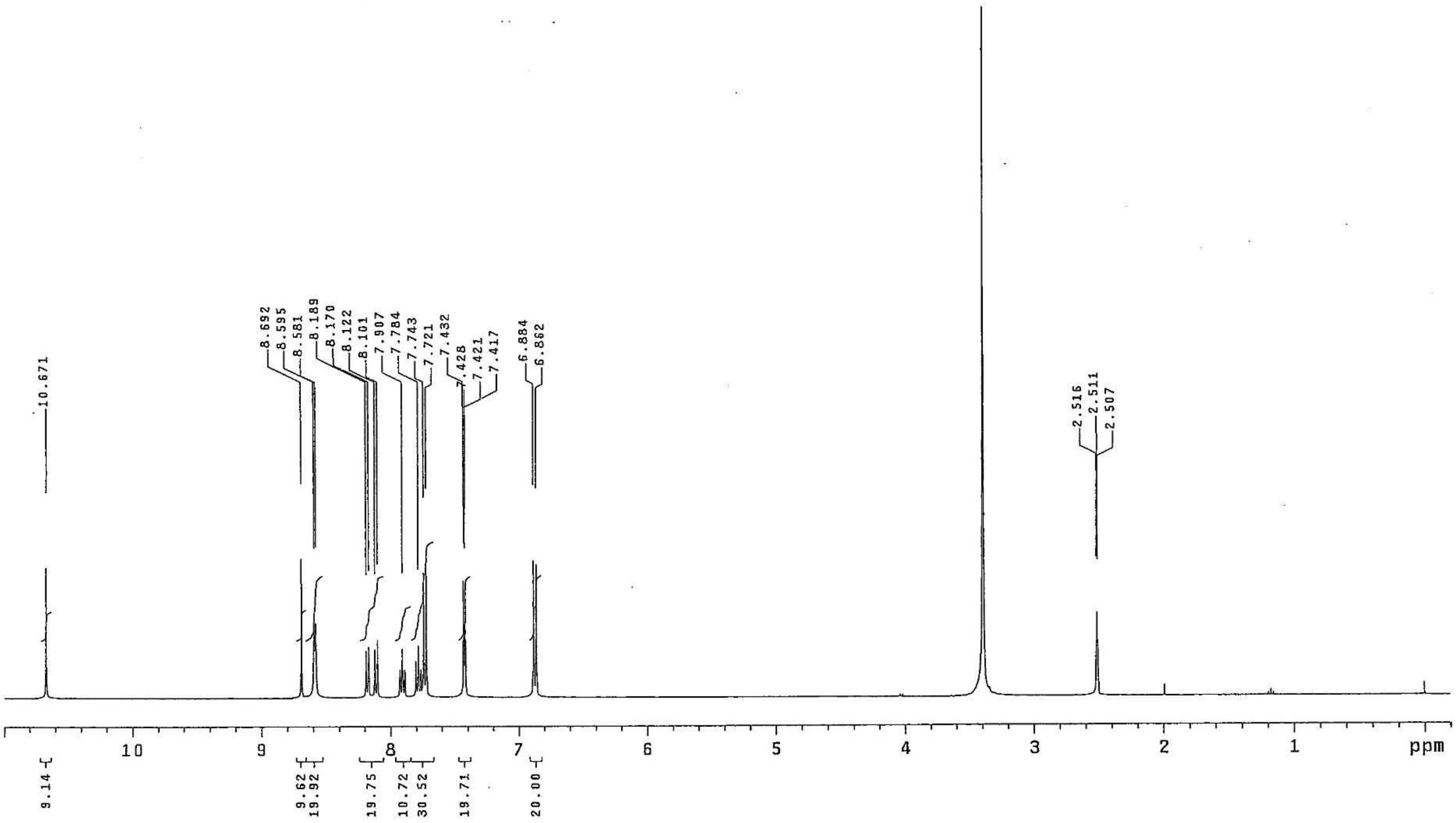

YCY-4864

Pulse Sequence: s2pul
UNITYplus-400 "unity400"
Date: Oct 3 2012
Solvent: DMSO
Ambient temperature
Total 1888 repetitions

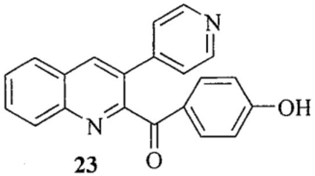

23

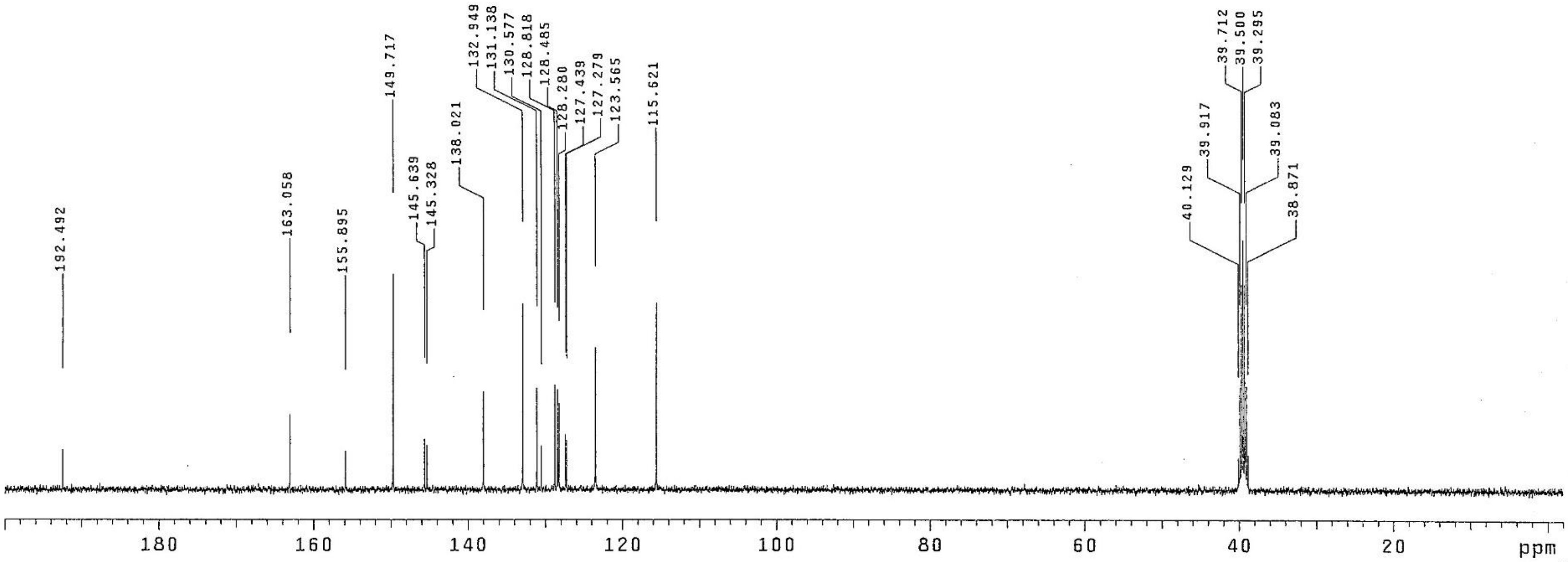

Supplement: Supplementary file 1 [file molecules-24-01162-s001.pdf]
